# Genome re-sequencing reveals the history of apple and supports a two-stage model for fruit enlargement

**DOI:** 10.1038/s41467-017-00336-7

**Published:** 2017-08-15

**Authors:** Naibin Duan, Yang Bai, Honghe Sun, Nan Wang, Yumin Ma, Mingjun Li, Xin Wang, Chen Jiao, Noah Legall, Linyong Mao, Sibao Wan, Kun Wang, Tianming He, Shouqian Feng, Zongying Zhang, Zhiquan Mao, Xiang Shen, Xiaoliu Chen, Yuanmao Jiang, Shujing Wu, Chengmiao Yin, Shunfeng Ge, Long Yang, Shenghui Jiang, Haifeng Xu, Jingxuan Liu, Deyun Wang, Changzhi Qu, Yicheng Wang, Weifang Zuo, Li Xiang, Chang Liu, Daoyuan Zhang, Yuan Gao, Yimin Xu, Kenong Xu, Thomas Chao, Gennaro Fazio, Huairui Shu, Gan-Yuan Zhong, Lailiang Cheng, Zhangjun Fei, Xuesen Chen

**Affiliations:** 1 State Key Laboratory of Crop Biology, Shandong Agricultural University, Tai’an, Shandong 271000 People’s Republic of China; 2 Shandong Centre of Crop Germplasm Resources, Shandong Academy of Agricultural Sciences, Jinan, Shandong 250100 People’s Republic of China; 3 Boyce Thompson Institute, Cornell University, Ithaca, NY 14853 USA; 4 State Key Laboratory of Crop Stress Biology in Arid Areas, College of Horticulture, Northwest A&F University, Yangling, Shaanxi 712100 People’s Republic of China; 5 Section of Horticulture, School of Integrative Plant Science, Cornell University, Ithaca, NY 14853 USA; 6 The Institute of Pomology, Chinese Academy of Agricultural Sciences, Xingcheng, Liaoning 125100 People’s Republic of China; 7 College of Forestry and Horticulture, Research Centre of Specialty Fruits, Xinjiang Agricultural University, Urumqi, Xinjiang 830000 People’s Republic of China; 8 Mudanjiang Branch of Heilongjiang Academy of Agricultural Science, Mudanjiang, Heilongjiang 157500 People’s Republic of China; 9 Key Laboratory of Biogeography and Bioresource in Arid Land, Xinjiang Institute of Ecology and Geography, Chinese Academy of Sciences, Urumqi, Xinjiang 830011 People’s Republic of China; 10 USDA-Agricultural Research Service, Plant Genetic Resources Unit, Geneva, NY 14456 USA; 11 USDA-Agricultural Research Service, Robert W. Holley Center for Plant and Health, Ithaca, NY 14853 USA

## Abstract

Human selection has reshaped crop genomes. Here we report an apple genome variation map generated through genome sequencing of 117 diverse accessions. A comprehensive model of apple speciation and domestication along the Silk Road is proposed based on evidence from diverse genomic analyses. Cultivated apples likely originate from *Malus sieversii* in Kazakhstan, followed by intensive introgressions from *M. sylvestris*. *M. sieversii* in Xinjiang of China turns out to be an “ancient” isolated ecotype not directly contributing to apple domestication. We have identified selective sweeps underlying quantitative trait loci/genes of important fruit quality traits including fruit texture and flavor, and provide evidences supporting a model of apple fruit size evolution comprising two major events with one occurring prior to domestication and the other during domestication. This study outlines the genetic basis of apple domestication and evolution, and provides valuable information for facilitating marker-assisted breeding and apple improvement.

## Abstract (web summary)

Apple is one of the most important fruit crops. Here, the authors perform deep genome resequencing of 117 diverse accessions and reveal comprehensive models of apple origin, speciation, domestication, and fruit size evolution as well as candidate genes associated with important agronomic traits.

## Introduction

Crop domestication is an artificial selection process, fostering co-dependence between human and crop plants. This process often includes an initial phase of unconscious selection by farmers for several thousand years, followed by a subsequent breeding phase of intentional selection of favorable traits by breeders^1^. As a result, cultivated crops diverge from their wild progenitors morphologically and genetically in response to human selection. Recently, rapid advances in sequencing technologies have allowed researchers to sequence entire crop genomes efficiently and the resultant information can help elucidate how human-involved evolutionary processes have shaped modern crop genomes, as demonstrated in peach^2^, date palm^3^, tomato^4^, maize^5^, soybean^6^, and rice^7^. These studies provide valuable information for facilitating future crop improvement and thus securing global food production.

Cultivated apple (*Malus domestica* Borkh.), one of the most widely produced and economically important fruit crops in temperate regions, has been domesticated from *M. sieversii* in the Tian Shan Mountains for 4000–10,000 years, and dispersed from Central Asia to West Europe along the Silk Road, allowing hybridization and introgression of wild crabapples from Siberia (*M. baccata* (L.) Borkh.), Caucasus (*M. orientalis* Uglitz.), and Europe (*M. sylvestris* Mill.)^8^. It has been reported that *M. sieversii* is the primary progenitor and *M. sylvestris* is a major secondary contributor of cultivated apples^9^. Centuries of human exploitation and selection have produced thousands of apple cultivars with diverse fruit sizes and flavors^10^. In addition, apples are characterized with lengthy juvenile phases, self-incompatibility, and vegetative propagation^8, 11^. These characteristics together with its unique domestication history indicate that the impact of domestication on the genomes of apples would be much different from that on the genomes of seed-propagated annual crops as well as self-compatible tree fruits such as peach. However, very limited information is available on how the apple genome has been reshaped by human selections and how small and sour wild ancestors have evolved into modern large and sweet apples. In this study, we performed deep genome sequencing of 117 diverse *Malus* accessions. The large amount of genomic variation resources has not only allowed us to gain new insights into apple evolution and domestication, but also provided invaluable information for accelerating the usage of wild species in apple breeding and improvement.

## Results

### Genome resequencing and variation calling

A total of 117 *Malus* accessions from 24 species were selected for genome sequencing, including 35 *M. domestica* (24 scion and 11 rootstock cultivars), 10 *M. sylvestris*, 29 *M. sieversii*, 9 *M. robusta*, 6 *M. baccata*, 4 *M. asiatica*, 4 *M. hupehensis*, and 20 in the remaining 17 wild species with one or two accessions per species (Supplementary Data 1). Among the 29 *M. sieversii* accessions, 15 originated in Kazakhstan, on the west side of the Tian Shan Mountains, and 14 were collected from natural forests in Xinjiang of China, on the east side of the Tian Shan Mountains. Six out of the 24 species are native to China, four native to North America, and two to Europe, while some *Malus* species are considered as intrageneric hybrids, such as *M. robusta* and *M. asiatica* (Supplementary Data 2). Chinese soft apples, such as “Pinpo”, “Xiangguo”, *M. asiatica* and *M. prunifolia* have been cultivated as dessert apples for more than 2000 years in China (Supplementary Note 1). Analysis of the phenotypical data recorded in the USDA-GRIN database (https://npgsweb.ars-grin.gov/) indicated that domesticated apples are significantly bigger, firmer, and sweeter than *M. sieversii* in Kazakhstan, while less acidulated and much bigger than *M. sylvestris* (Supplementary Fig. 1).

Resequencing of the 117 apple genomes generated a total of 1060 Gb high-quality cleaned sequences, with an average of 9.06 Gb per accession that represented ~12.2× of the apple genome (Supplementary Data 1). After aligning the reads to the pseudo-haplotype apple genome^12^ (v1.0p), we identified a final set of 7,218,060 single nucleotide polymorphisms (SNPs) (Supplementary Note 2 and Supplementary Tables 1 and 2). Furthermore, we identified 431,597 small insertions and deletions (indels). Polymerase chain reaction (PCR) amplification and Sanger sequencing on genomic regions containing 958 randomly selected SNP loci in six apple accessions indicated a high accuracy rate (98.1%) for our genotype calling.

### An apple evolutionary map

**Fig. 1:**
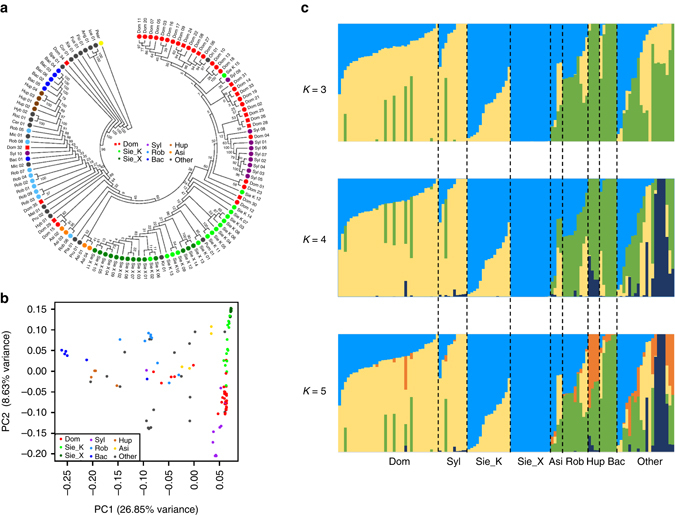
Population structure of 117 domesticated and wild apples. **a** Neighbor-joining phylogenetic tree constructed using SNPs at fourfold degenerate sites. Each species group is color coded, with *red squares* representing rootstocks and *red dots* scions. **b** Principal component analysis (*PCA*) of the 117 apple accessions. **c** Bayesian model-based clustering of the 117 apple accessions with the number of ancestry kinship (*K*) from 3 to 5. Each *vertical bar* represents one apple accession and the *x* axis shows different apple accessions. Each *color* represents one putative ancestral background and the *y* axis quantifies ancestry membership. Asi *M. asiatica*, Bac *M. baccata*, Dom *M. domestica*, Hup *M. hupehensis*, *Other* other wild species, Rob *M. robusta*, Sie_K *M. sieversii* in Kazakhstan, Sie_X *M. sieversii* in Xinjiang, Syl *M. sylvestris*

We first examined the phylogeny among wild and cultivated apples by constructing a neighbor-joining phylogenetic tree with pear as the outgroup using SNPs at fourfold degenerate sites (4D SNPs). The tree showed that accessions of *M. domestica* and its introgression contributor *M. sylvestris* formed a subclade within a large mixed clade comprising *M. sieversii* accessions that are considered as progenitors of cultivated apples^8, 9^, while accessions of other wild species position outside this domestication-related clade (Fig. 1a). Wild species native to North America (*M. ioensis*, *M. angustifolia*, *M. fusca*, and *M. coronaria*) are the closest to pear, followed by Asian wild species *M. baccata* and *M. hupehensis*. Notably, the recent introgression from *M. sylvestris* into *M. domestica* has been so intensive that the cultivated apples now appear to be closer to European crabapple *M. sylvestris* than to their progenitor *M. sieversii*, which is consistent with a previous report^9^. A principal component analysis (PCA) illustrated a similar pattern to the phylogenetic tree in that *M. domestica*, *M. sieversii*, and *M. sylvestris* accessions formed closely related clusters that were clearly separated from the dispersed accessions of other wild species (Fig. 1b).

**Fig. 2:**
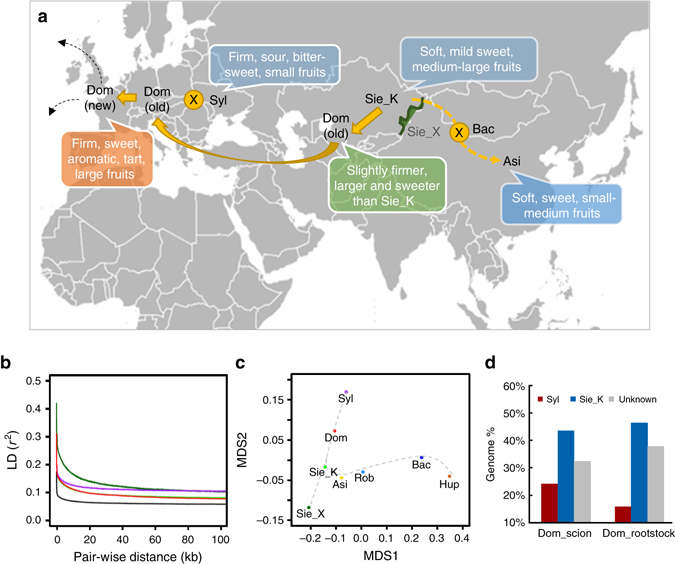
Apple evolutionary map. **a** Apple evolutionary map along the west and east bounds of the Silk Route with center of origin at Kazakhstan in central Asia. **b** Decay of linkage disequilibrium (*LD*) measured as the squared correlation coefficient (*r* ^2^) by pairwise physical distance in *M. domestica*, *M. sieversii* in Kazakhstan, *M. sieversii* in Xinjiang, *M. sylvestris*, and other wild species. **c** Multidimensional scaling (*MDS*) plot for the pairwise *F*_ST_ matrix. The Euclidean distances between each pair of groups significantly represent the corresponding *F*_ST_ values (Spearman rank-sum correlation *ρ* = 0.95; *p* < 10^−14^). **d** Major alleles of scion and rootstock cultivars derived from *M. sieversii* in Kazakhstan and *M. sylvestris*. Asi *M. asiatica*, Bac *M. baccata*, Dom *M. domestica*, Hup *M. hupehensis*, Rob *M. robusta*, Sie_K *M. sieversii* in Kazakhstan, Sie_X *M. sieversii* in Xinjiang, Syl *M. sylvestris*

To further understand the evolutionary history of apple, we used a Bayesian clustering algorithm with admixed models^13^ to estimate ancestry proportions for each accession (Fig. 1c). Our Δ*K* analysis revealed that five populations (*K* = 5) represent the best model for these 117 accessions (Supplementary Fig. 2 **)**. For *K* = 3, *M. domestica* and its wild relatives, *M. sieversii* and *M. sylvestris*, were clearly separated from other wild species, supporting the evolutionary history of domesticated apple^8, 9, 14^. With *K* from 4 to 5, two new subpopulations arose from the wild species other than *M. sieversii* and *M. sylvestris*, indicating their high diversity and further distance from domesticated apples. *M. sieversii* accessions from the two sides of the Tian Shan Mountains segregated into two different subpopulations reflecting their geographical distributions. *M. sieversii* accessions in Kazakhstan showed admixed ancestry possibly from hybridizations with wild apples such as *M. orientalis* along the Silk Road, and/or domesticated apples cultivated nearby, while Xinjiang accessions kept their homogeneous genetic background probably due to their geographical isolation that blocks interspecific hybridization. In addition to *M. sieversii* in Xinjiang, six other species in distinct habitats, such as *M. ioensis* and *M. angustifolia* in North America and *M. baccata* in East Asia, were also identified with homogeneous genetic background, which gave rise to other hybrid species and possess tremendous value in apple breeding practices (Supplementary Fig. 3). The structure of several hybrid species was consistent with their known pedigrees recorded in the USDA-GRIN database, including *M. asiatica*, *M. prunifolia*, *M. robusta*, and several rootstocks, while that of *M. platycarpa* did not agree with its pedigree in the database (Supplementary Figs. 4 and 5 and Supplementary Note 3). Together, these findings prompt us to propose a comprehensive apple evolutionary map across Eurasia continent, illustrating the initial domestication from *M. sieversii* in Kazakhstan, the hybridization between *M. sylvestris* and the ancient domesticated apples spread from Central Asia to Europe via the Silk Road westward, and the rise of orient hybrid species from crosses between *M. baccata* and *M. sieversii* in Kazakhstan distributed and cultivated along the Silk Road eastward (Fig. 2a and Supplementary Note 4). During the domestication process, cultivated apples retained the large fruit size from *M. sieversii*, gained the firm texture and appetizing flavor from the hybridization with *M. sylvestris* and continued to be bred into larger and firmer fruit with better flavor and aroma.

We then evaluated the genetic diversity of different apple subpopulations. The genome-wide nucleotide diversity (*π*) of *M. domestica* (2.20 × 10^−3^) was lower than that of *M. sieversii* in Kazakhstan (2.35 × 10^−3^), *M. sylvestris* (2.55 × 10^−3^), and other wild species (4.26 × 10^−3^), while *M. sieversii* in Xinjiang exhibited the lowest diversity level (1.30 × 10^−3^). Compared to other domesticated perennial crops, the nucleotide diversity in cultivated apple is higher than that of peach^2^ (1.5 × 10^−3^), but lower than that of cassava^15^ (2.6 × 10^−3^) and date palm^3^ (9.2 × 10^−3^). The similar level of nucleotide diversity between *M. domestica* and its progenitor *M. sieversii* in Kazakhstan indicated a very weak bottleneck, if any, during apple domestication, consistent with findings in previous studies^8, 9^. Linkage disequilibrium (LD) analyses for each group further supported a very weak and nearly undetectable domestication bottleneck (Fig. 2b and Supplementary Note 5). The rapid LD decay of domesticated apple suggested that a large set of markers densely covering the genome is preferred for a high-resolution population genetic analysis, as indicated in a recent study^16^. Here we also demonstrated the power of our high-density SNPs in enhancing the resolution of genome-wide association studies (GWAS) (Supplementary Note 6, Supplementary Fig. 6, Supplementary Table 3, and Supplementary Data 3 and 4).

To further investigate population divergence among different species groups, we computed pairwise *F*_ST_ values, demonstrating consistent relationships among the subpopulations with our proposed evolutionary scenario (Fig. 2c and Supplementary Fig. 7). Genome-wide inference of major allele origins in *M. domestica* from *M. sieversii* in Kazakhstan or *M. sylvestris* revealed that 46% of the *M. domestica* genome was probably derived from its progenitor *M. sieversii* in Kazakhstan and 21% from its secondary contributor *M. sylvestris*, while the origin of the remaining 33% was uncertain. The genomic introgression from *M. sylvestris* to scion cultivars is about 10% higher than that to rootstock cultivars, raising the possibility that *M. sylvestris* may have contributed important alleles for fruit quality and production traits to dessert apple cultivars (Fig. 2d and Supplementary Fig. 8).

### Differential selection of domesticated apples

**Fig. 3:**
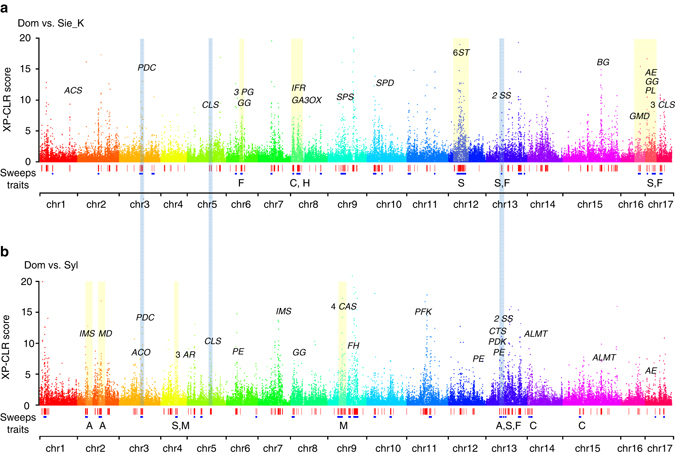
Genome-wide distribution of selective sweeps in *M. domestica*. **a** Selective sweeps in *M. domestica* compared with *M. sieversii* in Kazakhstan. **b** Selective sweeps in *M. domestica* compared with *M. sylvestris*. XP-CLR scores are plotted across the 17 chromosomes in the apple genome with key functional enzyme genes labeled above the dot peaks. *Red vertical boxes* illustrate selective sweeps, and *blue boxes* represent local GO enriched regions with associated traits labeled below. Selective regions shared by both comparisons are shaded with *light blue bars*, while interesting regions only identified in one of the two comparisons are shaded with *light yellow bars*. Traits include fruit acidity (A), color (C), firmness (F), hormone (H), soluble sugar (S), and secondary metabolites (M). Gene abbreviations: *ALMT* aluminum-activated malate transporter, *ACO* 1-aminocyclopropane-1-carboxylate oxidase, *ACS* 1-aminocyclopropane-1-carboxylate synthase, *AE* aldose 1-epimerase, *AR* aldose reductase, *BG* beta-galactosidase, *CAS* cycloartenol synthase, *CTS* citrate synthase, *CLS* cellulose synthase, *FH* flavanone 3-hydroxylase, *GA3OX* gibberellin 3-beta-dioxygenase, *GG* glucan endo-1,3-b-glucosidase, *GMD* GDP-mannose 4,6-dehydratase, *IFR* isoflavone reductase, *IMS*, 2-isopropylmalate synthase, *MD* malate dehydrogenase, *PDC* pyruvate decarboxylase, *PDK* pyruvate dehydrogenase kinase, *PE* pectin esterase, *PFK* 6-phospho-fructokinase, *PG* polygalacturonase, *PL* pectate lyase, *SPD* sorbitol 6-phosphate dehydrogenase, *SPS* sucrose phosphate synthase, *SS* sucrose synthase, *ST* sugar transporter

Considering the remarkable role of *M. sylvestris* in shaping modern domesticated apples, genomic regions dramatically affected by selection during apple domestication were identified in two contrasts: *M. domestica* vs. *M. sieversii* in Kazakhstan for initial domestication (*Dom_SieK*) and *M. domestica* vs. *M. sylvestris* for secondary introgression (*Dom_Syl*) (Fig. 3 and Supplementary Figs. 9 and 10). The selected regions of *Dom_SieK* and *Dom_Syl* had a mean size of 33.1 and 42.8 kb, covered a total length of 13.9 Mb (3.7 % of genome) and 17.2 Mb (4.6%), and harbored 840 and 1089 genes, respectively, among which 246 (29.3%) and 336 (30.9%) showed differential expression during apple fruit development (Supplementary Data 5–8). The identified selective sweeps are enriched with genes associated with fruit sugar content, firmness, color, hormone, and secondary metabolism in both *Dom_SieK* and *Dom_Syl* contrasts, while genes related to fruit acidity were only enriched in *Dom_Syl* (Fig. 3 and Supplementary Data 9). Notably, the enriched genes included those encoding six sugar transporters, several key enzymes in the glycolysis/gluconeogenesis pathway, two sucrose synthases and three cellulose synthases in *Dom_SieK*, two aluminum-activated malate transporters, a malate dehydrogenase, and a citrate synthase in *Dom_Syl*, and two sucrose synthases, one pyruvate decarboxylase, and one cellulose synthase in both *Dom_SieK* and *Dom_Syl*, highlighting the constant selection of sweet and firm fruits in the history of apple domestication (Fig. 3). Together, these candidate domestication-related genes are indicative of different selective forces for improving different agronomic traits from the two wild contributors during domestication.

We further scanned SNPs that were highly divergent between *M. domestica* and different wild species groups using the top 1% *F*_ST_ values (Supplementary Data 10). A large number of disease resistance (R) genes and genes involved in various different abiotic stresses were found to contain non-synonymous SNPs highly divergent between *M. domestica* and other wild species groups, suggesting their adaptations to different growth environments. Within *M. domestica* accessions, genes underlying dwarf quantitative trait loci (QTLs) (*Dw1* and *Dw2*)^17, 18^, R genes and receptor kinase genes were identified to have non-synonymous SNPs highly divergent between rootstock and scion cultivars, which could facilitate the study of important apple rootstock traits, such as dwarfing, precocity, and disease resistance (Supplementary Data 11). The highly divergent SNPs and the associated genes discovered in this study provided ample information for broadening our understanding of apple speciation, differentiation, and evolution.

### Increase of fruit size prior to and during apple domestication

**Fig. 4:**
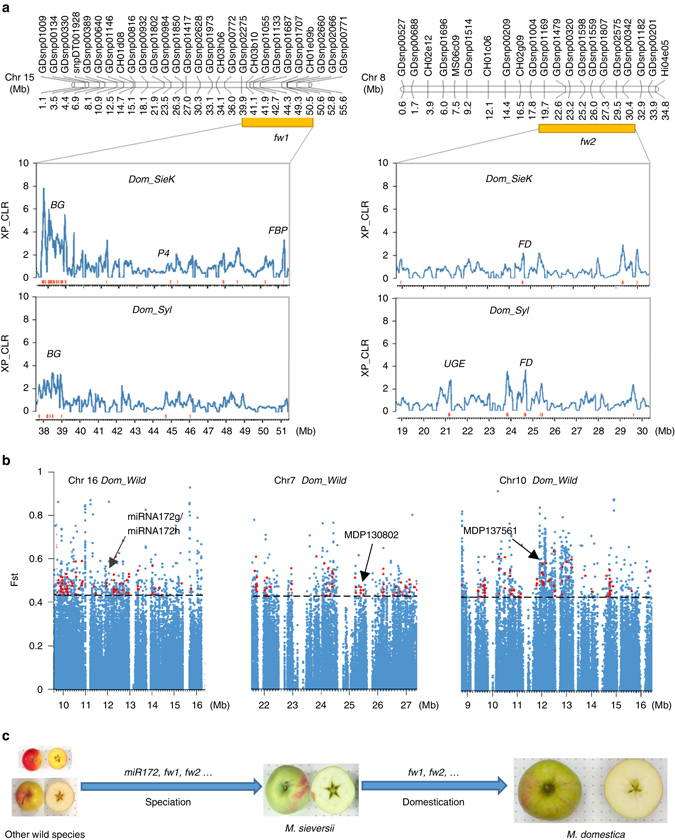
Evolution of fruit size during speciation and domestication in apple. **a** Domestication sweeps underlying apple fruit size QTLs. Within the physical intervals (*orange boxes*) of the two fruit weight QTLs *fw1* and *fw2*, distributions of XP-CLR scores are shown. Selective sweeps are marked with *red bars* and interesting genes are labeled above peaks. **b** *MiRNA172g/miRNA172h* and the two target genes that contain highly divergent SNPs (pointed by *arrows*) between *M. domestica* with large fruits and other wild species bearing very small fruits. **c** Schematic diagram of the two-step evolution of apple fruit size. *BG* beta-galactosidase, *FBP* fructose-1,6-bisphosphatase, *FD* ferredoxin, *PPD* pyruvate phosphate dikinase, *P4* patellin-4, *UGE* UDP-glucose 4-epimerase

One essential aspect of domestication process for most crop species is to increase fruit and/or seed size, which is also referred to as “domestication syndrome”^19^. Fruit sizes of *M. sylvestris* are significantly smaller than those of both *M. domestica* and *M. sieversii*, while domesticated apples have larger fruits than *M. sieversii* (Supplementary Fig. 1d, e). Two previously reported fruit weight QTLs^20^ (designated as *fw1* on chromosome 15 and *fw2* on chromosome 8) were found to be co-located with selective sweeps (Fig. 4a). QTLs *fw1* and *fw2* harbor 11 and 7 genes, respectively, in selective regions of *M. domestica* from *M. sieversii*, and 8 and 21 genes, respectively, in sweeps from *M. sylvestris* (Supplementary Data 12). Genes encoding regulators of cell division, such as *fw2.2* in tomato^21^ and *CNR1* in maize^22^, have been reported to control organ size, and recently a β-galactosidase gene was found to be involved in the regulation of fruit weight and size in strawberry, besides fruit softening^23^. We found that one cell division regulatory gene (MDP0000223854) and two β-galactosidase genes (*MDP0000921848* and *MDP0000179821*) in *fw1* were under human selection. Interestingly, the β-galactosidase gene, *MDP0000179821*, was in selected regions of both *Dom_SieK* and *Dom_Syl* and showed differential expression during apple fruit development with highest expression at the active cell division stage (Supplementary Data 12). Furthermore, several cell division regulatory genes (e.g., *MDP0000555176*, *MDP0000802780*, *MDP0000846861*, and *MDP0000681201*) and a gene (*MDP0000241347*) homologous to rice *GS3* that controls grain size^24^ were found in several other selected regions, and they all showed highest expression at the active cell division stage of fruit development (Supplementary Data 6 and 8), suggesting their potential contribution to the increase of fruit size during apple domestication.

*MiRNA172p* was recently reported to regulate apple fruit size by targeting AP2 transcription factors^25^. Using their precursor sequences, the 16 miRNA172 genes identified in apple^26^ were clustered into four clades (Supplementary Fig. 11 **)**. We detected a highly differentiated SNP only between domesticated and other wild apples in the precursor sequences of *miRNA172g* and *miRNA172h*, two miRNAs sharing the same precursor sequences (Fig. 4b). No differentiated SNPs were detected in the 16 miRNA172 genes between *M. domestica* and *M. sieversii*. Furthermore, we found that five out of the eight target AP2 genes of *miRNA172g* and *miRNA172h* carried highly differentiated SNPs in the comparison between *M. domestica* and other wild species, two of which comprised non-synonymous SNPs (Fig. 4b and Supplementary Fig. 11 **)**. Therefore, besides the previously discovered *miRNA172p* ^25^, we identified two additional miRNA172 genes (*miRNA172g* and *miRNA172h*) that might have contributed to the increase of fruit size during *Malus* speciation prior to domestication.

Taken together, we propose a two-step evolution model for fruit size enlargement in apple to characterize its unique evolution process (Fig. 4c). Unlike modern maize and tomato whose domestication process started with ancestral species bearing very small seeds or fruits^4, 5^, apple domestication was initiated from *M. sieversii* whose fruits are larger than all other wild apples^8^. As fruit size is one of the most desirable traits for crop domestication and improvement, apple domestication started with a great advantage and much lower evolutionary pressure than other crops. The finding of fruit weight QTLs, and *miRNA172s*, and their target genes from comparisons between small-fruited wild apples and large-fruited cultivars helped explain why a weak selection in a highly heterozygous perennial crop can still yield favorable large fruits. The mild increase in fruit size during several thousand years of domestication after speciation, partially contributed by QTLs *fw1* and *fw2*, suggests that apple fruit size has great potential to be increased in future breeding practices, considering that the modern tomato fruit is approximately 100 times larger than its direct wild progenitor.

### Enhancement of fruit firmness during apple domestication

**Fig. 5:**
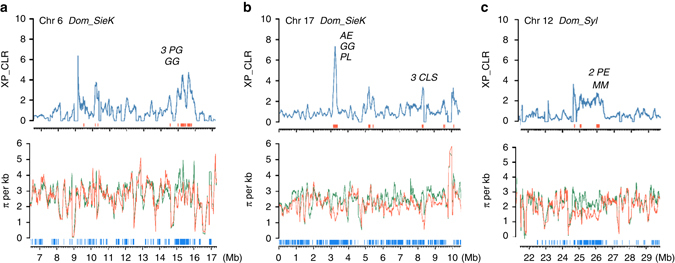
Domestication sweeps underlying apple fruit firmness. Distributions of XP-CLR scores and nucleotide diversity (π) in selective regions on chromosomes 6 (**a**) and 17 (**b**) in *M. domestica* from *M. sieversii* in Kazakhstan (*Dom_SieK*) and chromosome 12 (**c**) from *M. sylvestris* (*Dom_syl*), which harbor genes known to be associated with fruit firmness. Distributions of XP-CLR scores are shown in *top panels* with selective sweeps marked with *red bars* and interesting genes labeled above peaks. Distributions of π are shown in *bottom panels* with *M. domestica* in *orange lines*, and *M. sieversii* and *M. sylvestris* in *green lines*. *AE* aldose 1-epimerase, *CLS* cellulose synthase, *GG* glucan endo-1,3-b-glucosidase, *MM* endo-beta-1,4-mannase, *PE* pectinesterase, *PG* polygalacturonase, *PL* pectate lyase

Besides large fruits, humans also have been selecting firm flesh texture, not only for crispy taste, but also for a longer shelf life, better post-harvest disease resistance, and reduced bruising during harvest and transportation. Fruit firmness is directly linked to enzyme-mediated cell wall modification^27^. To decipher the genetic mechanism underlying the selection for firm apples, we mined selective sweeps for genes that were potentially involved in regulating fruit firmness. We found a region on chromosome 16 was under intensive human selection in the *Dom_SieK* contrast, harboring genes encoding key cell wall modifying enzymes including three polygalacturonases (*MDP0000512850*, *MDP0000939625*, and *MDP0000873268*), and one glucan endo-1,3-beta-glucosidase (*MDP0000295938*) (Fig. 5a). Furthermore, several other selective regions were also found to contain genes related to cell wall modifications. For example, one selective sweep on chromosome 17 from the *Dom_SieK* comparison comprised three cellulose synthase genes (*MDP0000190520*, *MDP0000289339*, and *MDP0000184309*), and another sweep from the same chromosome harbored one pectate lyase (*MDP0000301545*), one glucan endo-1,3-beta-glucosidase (*MDP0000206670*), and one aldose 1-epimerase (*MDP0000737131*) (Fig. 5b). Similarly, another sweep on chromosome 12 from the *Dom_Syl* contrast harbored one endo-beta-1,4-mannase (*MDP0000832632*) and two pectinesterase genes (*MDP0000278119* and *MDP0000278118*) (Fig. 5c). A number of these cell wall-related genes were differentially regulated during fruit development (Supplementary Data 6 and 8). Therefore, the evolution of these genes might have contributed to the firm fruit texture of domesticated apples.

### Enrichment of fruit flavor during apple domestication

**Fig. 6:**
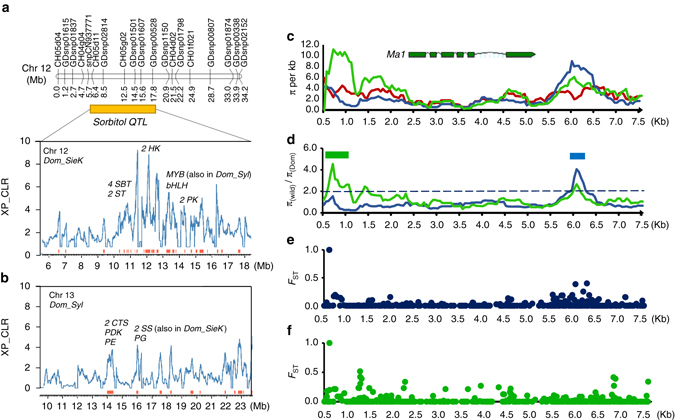
Domestication of fruit sweetness and acidity in apples. **a** Selective sweeps from *M. sieversii* in Kazakhstan that co-localize with a sorbitol QTL. **b** Domestication sweeps from *M. sylvestris* that contain key genes for sugar metabolism. Distributions of XP-CLR scores are shown. Selective sweeps are marked with *red bars* and interesting genes are labeled above peaks. **c**–**f** Domestication of the *Ma1* gene that regulates apple fruit acidity. **c** Distributions of nucleotide diversity (*π*) of *M. domestica* (*red*), *M. sieversii* in Kazakhstan (*blue*), and *M. sylvestris* (*green*) in the *Ma1* genome region. **d** *Ma1* selective sweeps during domestication from *M. sieversii* in Kazakhstan (*blue*) and *M. sylvestris* (*green*). Sweep regions are marked with *filled boxes*. **e** Distribution of *F*_ST_ between *M. domestica* and *M. sieversii* in Kazakhstan in the *Ma1* genome region. **f** Distribution of *F*_ST_ between *M. domestica* and *M. sylvestris* in the *Ma1* genome region. *bHLH* transcription factor, *CTS* citrate synthase, *HK* hexokinase, *MYB* transcription factor, *PDK* pyruvate dehydrogenase kinase, *PE* pectin esterase, *PG* polygalacturonase, *PK* pyruvate kinase, *SBT* sorbitol transporter, *SS* sucrose synthase, *ST* sugar transporter

Fruit flavor, mainly a balance between sugars and acids, is another important trait under human selection. Although it has been reported that fruit acidity rather than sweetness is likely to undergo artificial selection^28^, we found that both fruit sugar content and acidity have been altered during apple domestication using historical phenotype data in the USDA-GRIN database (Supplementary Fig. 1). During the domestication from *M. sieversii* in Kazakhstan, a region on chromosome 12 has undergone intensive selection and co-localizes with a sorbitol QTL^29^. Interestingly, genes encoding four sorbitol transporters and two sugar transporters cluster within this sorbitol QTL under selection, and all four sorbitol transporter genes were differentially expressed during apple fruit development (Fig. 6a and Supplementary Data 13). Similarly, another genomic region on chromosome 13 enriched with genes encoding key enzymes of sugar metabolism is under intensive selection from *M. sylvestris* (Fig. 6b). Notably, the two sucrose synthase genes in this region are also in selective sweeps from *M. sieversii* in Kazakhstan.

To study fruit acidity during apple domestication, we investigated the nucleotide diversity of gene *Ma1*, which underlies the major fruit acidity QTL *Ma* and encodes an aluminum-activated malate transporter^30^. We identified a region around 1.5 kb upstream of *Ma1* coding sequence (CDS) with substantially reduced nucleotide diversity in *M. domestica* compared to *M. sylvestris*, and another region about 750 bp downstream of *Ma1* CDS when compared to *M. sieversii* in Kazakhstan (Fig. 6c–f), indicating that the *Ma1* might be under strong selection during apple domestication. The distributions of *F*_ST_ of SNPs surrounding the *Ma1* gene region further underpinned the selective sweep regions. In addition to *Ma1*, two other aluminum-activated malate transporter genes and a gene encoding malate dehydrogenase were identified in selection sweeps from *M. sylvestris*, assisting in explaining the significantly reduced acidity level in domesticated apples when compared to *M. sylvestris* (Fig. 3 and Supplementary Fig. 1).

## Discussion

We report here a comprehensive genome variation map of apple through deep resequencing the genomes of 117 wild and cultivated apples. The population analyses reveal that modern cultivated apples originate from *M. sieversii* in Kazakhstan with intensive recent introgressions from *M. sylvestris*, while *M. sieversii* in Xinjiang is an isolated ecotype with a homogeneous genetic background that holds great potential for future apple improvement. Chinese native species, such as *M. asiatica* and *M. prunifolia*, are very likely to be hybrids between *M. baccata* and *M. sieversii* in Kazakhstan. The similar level of genetic diversity between *M. domestica* and its direct wild progenitor *M. sieversii* in Kazakhstan is consistent with the comparably fast LD decays in these two species and an undetectable bottleneck in apple domestication. The weak selection during apple domestication might be explained by its unique physiological characteristics, such as self-incompatibility, lengthy juvenile phases, and limited sexual reproduction. However, several thousand years of selection still has accumulated a considerable amount of desirable fruit traits that make apple one of the most important fruit crops worldwide. We identified selective sweeps underlying fruit size, flesh firmness, sweetness, and acidity, as well as highly divergent SNPs between rootstock and scion cultivars that are associated with dwarfing and disease resistance. The identified large set of SNPs, highly confident candidate genes associated with important apple traits, and the insight gained regarding apple evolution and domestication provide valuable information for facilitating future apple marker assisted breeding, QTL mapping, and population-structured association studies.

## Methods

### Sample preparation and sequencing

A total of 117 apple accessions were included in this study, of which 70 were provided by USDA-ARS Plant Genetics Resources Unit (Geneva, NY, USA), 33 by Institute of Pomology of Chinese Academy of Agricultural Sciences (Xingcheng, Liaoning, China), and 14 were collected from the Tian Shan Mountains (Xinjiang, China). Genomic DNA was extracted from young leaves using the DNeasy Plant Mini Kit (Qiagen, Venlo, the Netherlands). Paired-end Illumina genomic libraries were prepared and sequenced on an Illumina Hiseq 2500 platform following the manufacturer’s instructions (Illumina, San Diego, CA).

### Mapping and variant calling

The Illumina paired-end reads from each apple accession were first processed to collapse duplicated read pairs into unique read pairs. Duplicated read pairs were defined as those having identical bases at positions of 15-90 in both left and right reads. The resulting unique reads were processed to remove adaptor and low-quality sequences using Trimmomatic^31^. The cleaned unique reads were aligned to the apple reference genome^12^ (v1.0p, https://www.rosaceae.org/species/malus/malus_x_domestica/genome_v1.0p) using BWA^32^, and only uniquely mapped reads were retained. Following mapping, genotypes were assigned to each genomic position for each accession based on the alignment mpileup files generated by SAMtools^33^. SNPs and small indels in the population of 117 apple accessions were then identified if they were supported by at least two mapped reads, and had an allele frequency ≥0.3. Furthermore, SNP sites with the nearest SNPs less than 5 bp away, containing only heterozygous SNPs, or with more than 50% missing data were excluded.

To validate the SNP calling, we randomly selected 958 homozygous SNP loci across the genome and PCR-amplified and Sanger-sequenced the corresponding genomic regions in six apple accessions (*M. domestica*, Dom_01; *M. sieversii*, Sie_K_04, Sie_X_03 and Sie_X_09; *M. sylvestris*, Syl_01; and *M. hupehensis*, Hup_01).

### Phylogenetic and population structure analyses

We selected a total of 24,326 SNPs at fourfold degenerate sites (4D SNPs) with minor allele frequency (MAF) ≥5% and missing rate per site ≤10% for phylogenetic and population structure analyses. The rationale for choosing 4D SNPs is that they do not cause amino-acid changes and thus, should be under less selective pressure and more reliably reflect population structure and demography^18^. PCA was performed using program EIGENSOFT^34^. A neighbor-joining phylogenetic tree was constructed using MEGA6^35^ in 1000 bootstrap replicates with pear “*Pyrus* × *bretschneideri*” as the outgroup. Pear genomic sequence reads^36^ were downloaded from the NCBI SRA database (accession no. SRP016889) and aligned to the apple genome v1.0p for SNP calling. Population structure was investigated using STRUCTURE^13^. To determine the most likely number of ancestral kinships (*K*) in the apple population, STRUCTURE was run 20 times on 2000 randomly selected 4D SNPs for each *K* value from 3 to 19. The statistic “*∆K*”, which indicates the change in likelihood of different numbers of clusters, was calculated, and the cluster number with the highest ∆*K* value, which indicated the most likely number of clusters in the population, was obtained. We then used the whole set of 4D SNPs to calculate the cluster membership for each accession with 10,000 iterations. According to the phylogenetic and population structure analyses, nine accessions (*M. domestica*, Dom_15, Dom_29, Dom_32, Dom_34, and Dom_35; other wild species, Kir_01, Ori_01, and Pum_01; *M. sylvestris*, Syl_10) were positioned into unexpected species groups and thus excluded from downstream population genetics analyses.

### Inferring population genetic statistics

Nucleotide diversity (*π*) is often used as a measurement of the degree of the genotype variability within a population or species^37^. Values of *π* were calculated for 10-kb non-overlapping windows across the apple genome with the final set of 7,218,060 SNPs using the BioPerl module PopGen^38^.

Population differentiation is evaluated by the fixation index *F*_ST_^39^_._ Pairwise *F*_ST_ values were calculated among the eight species groups listed in the Supplementary Data 1 for the final SNP set using a variance component approach implemented in the R package HIERFSTAT^40^. To visualize the 8 × 8 pairwise *F*_ST_ matrix, multidimensional scaling (MDS) was performed using an R function *cmdscale* to transfer *F*_ST_ values into two dimensional values for plotting. To test the accuracy of this MDS visualization, the Spearman rank-sum correlation *ρ* was calculated between Euclidean distances between all pairs of values in the MDS plot and the corresponding *F*_ST_ values. SNPs with top 1% *F*_ST_ values were identified as highly divergent variations in comparisons between *M. domestica* and each of the four wild groups, including *M. sieversii* in Kazakhstan, *M. sieversii* in Xinjiang, *M. sylvestris*, and other wild species, as well as between rootstock and scion cultivars. To better reflect selective pressure on nucleotide variants, non-synonymous SNPs were further identified from the highly divergent SNPs with top 1% *F*_ST_ values, and the corresponding genes containing these non-synonymous SNPs were determined.

### LD

Correlation coefficient (*r* ^2^) of genotypes was calculated to measure LD level in each of the five groups using Haploview^41^ with the following parameters: -nogui -minMAF 0.10 -hwcutoff 0.001 -dprime. SNPs within each group were extracted for the analysis. SNPs within 500-kb sliding windows were used to estimate average *r* ^*2*^ at various physical distance classes, and the LD decay was plotted as a function of the derived average *r* ^*2*^ and physical distances. Tag SNPs were also identified using Haploview with parameters set as follows: -nogui -minMAF 0.05 -hwcutoff 0.001 -dprime -blockoutput GAB -minGeno 0.8 -pairwiseTagging.

### Inferring-derived alleles

To evaluate whether major alleles of *M. domestica* (*Dom*) are derived from those of *M. sieversii* in Kazakhstan (*Sie_K*) or *M. sylvestris* (*Syl*), the entire genome was scanned using SNPs with MAF ≥5% and missing rate per site ≤10%. First, allele frequency per site was computed in each species group and the allele with the frequency >0.6 was assigned as the major allele. For each site, we considered the major allele in *Sie_K* as the ancestral allele in *Dom* if these two groups share consistent major alleles; similarly, *Syl* was considered as the allelic ancestor for *Dom* when they had the same major alleles. For each non-overlapping 100-kb window, if the count of ancestral alleles as *Sie_K* was greater than two times of that as *Syl* and the total SNP count was larger than five, we regarded this region as being derived from *Sie_K*; similarly, if the ancestral-allele count ratio between *Syl* and *Sie_K* was larger than two and the window contained more than five SNPs, we considered this window as originating from *Syl*.

### Identification of selective sweeps

Selective sweeps across the genome were identified using a XP-CLR method that models the allele frequency spectrum to search for signals of differentiation between two populations^42^, following previous studies^43, 44^ with modifications. Briefly, selection screening was performed in two contrasts: *M. domestica* vs. *M. sieversii* in Kazakhstan for initial domestication (*Dom_SieK*) and *M. domestica* vs. *M. sylvestris* for introgressive domestication (*Dom_Syl*). Genetic distances between adjacent SNPs were calculated according to their physical distances in a widely used genetic map reported with the apple reference genome^12^. Parameters “-w1 0.0005 100 100 -p0 0.7” were adopted to run the program for each chromosome, allowing XP-CLR scores to be calculated for each 100-bp region using unphased SNPs with correlation level at 0.7 and no more than 100 SNPs per window (in size of 0.05 cM). The XP-CLR scores per 100 bp were averaged across non-overlapping 10-kb windows on each chromosome. Adjacent 10-kb windows with an average XP-CLR score no less than 80% of the genome-wide average XP-CLR score were joined as putative selective regions, which were further merged together if two regions were separated by only one low-score 10-kb window. The maximum of window-wise average XP-CLR scores in a merged region was assigned as the region-wise XP-CLR score. Merged regions with top 10% of region-wise XP-CLR scores were considered as candidate selective sweeps. To improve the prediction accuracy, only candidate selective sweeps having top 50% of *π* ratios between the ancestral and domesticated species were kept.

Genes in the candidate selective sweeps were grouped by their physical locations into each 1-Mb non-overlapping segments throughout the genome. Adjacent segments and segments separated by no more than 1-Mb were merged together, while segments containing no more than five genes were discarded. GO term enrichment analyses were conducted for genes in each merged segment along the genome using GO::TermFinder^45^.

### Genome-wide association study

The USDA-GRIN database archives phenotypic observations for 105 different traits in 4123 apple accessions from 1995 to 2014 (https://npgsweb.ars-grin.gov/gringlobal/cropdetail.aspx?type=descriptor&id=115). The phenotypic scores of fruit flavor, weight, width, length, flesh firmness, skin color, and soluble solid content were downloaded for 66 out of the 70 accessions from USDA-ARS that are included in the USDA-GRIN database. Phenotype scores recorded in multiple years for a particular accession were screened to exclude outliers (more than 1.5 times the interquartile range below the first quartile or above the third quartile) and then averaged across the years. Tukey’s HSD mean separation tests were conducted to analyze these phenotypes in pairwise comparisons between *M. domestica*, *M. sieversii* in Kazakhstan, and *M. sylvestris*.

Phenotypic data of skin color was used for the association study. To improve phenotyping of fruit skin color, we manually examined fruit pictures in the USDA-GRIN database and assigned a score of fruit skin color for each accession using the following scoring system: 0, *green*; 1, *yellow*; 2, *light red* (*green base* with *pink-red* area and/or stripes less than half of the fruit); 3, *medium red* (*green-yellow* base with blush more than half of the fruit); 4, *red* (without *green* or *yellow base*); 5, *dark red*. Same was done on 13 accessions collected from China (Supplementary Table 3). The final set SNPs were filtered by keeping those with missing rate ≤10%, MAF ≥5%, SNP calling rate per accession ≥80%, and *P* value of Hardy-Weinberg equilibrium test ≤0.001. The resulting SNPs were used to perform GWAS for fruit skin color using a linear mixed model (LMM) implemented in the FaST-LMM program^46^. Raw *P* values were adjusted for multiple testing using the Benjamini-Hochberg procedure^47^ and a significant association was based on a threshold of false discovery rate of 0.1 (corresponding to raw *P* value of 3.03 × 10^−6^).

### RNA-seq data generation and gene expression analysis

Fruit samples were taken at five stages of fruit development from the apple cultivar “Greensleeves”: 18 days after bloom (DAB) (active cell division), 37 DAB (the end of cell division), 67 DAB (rapid cell expansion), 90 DAB (rapid cell expansion), and 132 DAB (ripening). Three biological replicates were collected for each stage. Fresh tissues were immediately frozen in liquid nitrogen and ground to fine powder. Total RNA was extracted using the QIAGEN RNeasy Plant Mini Kit (Qiagen, USA) followed by treatment with RNase-free DNase I (Promega, USA) according to the manufacturers’ protocols. Strand-specific Illumina RNA-Seq libraries were constructed following the protocol described in Zhong et al.^48^ and sequenced on the Illumina HiSeq 2000 system using single-end 51-bp mode. Around 20–50 million reads were generated for each of the 15 libraries. Raw reads were first processed to remove adapter and low-quality sequences using Trimmomatic^31^. Reads shorter than 40 bp after trimming were discarded. The resulting reads were then aligned to the ribosomal RNA database^49^ using Bowtie^50^, allowing up to three mismatches. The aligned reads were discarded and the remaining reads were aligned to the apple genome (v1.0) using HISAT^51^. Raw counts for each apple gene were derived from the read alignments and normalized to reads per kilobase of exon model per million mapped reads (RPKM). Differential expression analyses were performed using edgeR^52^. Genes with adjusted *P* values less than 0.01, expression changes of at least twofold, and expression levels of at least 1 RPKM in at least one of the five stages were identified as differentially expressed genes during fruit development.

### Data availability

Sequence reads of genome and transcriptome resequencing have been deposited into the NCBI sequence read archive under accessions SRP075497 and SRP102350, respectively. SNP data sets can be accessed at ftp://bioinfo.bti.cornell.edu/pub/Apple_SNP/.

## Electronic supplementary material

Supplementary Information

Supplementary Data 1

Supplementary Data 2

Supplementary Data 3

Supplementary Data 4

Supplementary Data 5

Supplementary Data 6

Supplementary Data 7

Supplementary Data 8

Supplementary Data 9

Supplementary Data 10

Supplementary Data 11

Supplementary Data 12

Supplementary Data 13
