# Supplementary material for: Genome re-sequencing reveals the history of apple and supports a two-stage model for fruit enlargement
Source: Nat Commun. 2017 Aug 15;8:249. doi: 10.1038/s41467-017-00336-7 (PMC5557836; doi:10.1038/s41467-017-00336-7)
Supplement: Supplementary file 1 — Supplementary Information [file 41467_2017_336_MOESM1_ESM.pdf]

File Name: Supplementary Information
Description: Supplementary Figures, Supplementary Tables, Supplementary Notes and Supplementary References

File Name: Supplementary Data 1
Description: Statistics of apple genome resequencing for each accession

File Name: Supplementary Data 2
Description: List of Malus species groups in this study with habitat and pedigree information from the USDA GRIN database

File Name: Supplementary Data 3
Description: List of SNPs significantly associated with fruit skin color in apple

File Name: Supplementary Data 4
Description: List of genes in the region highly associated with the fruit skin color

File Name: Supplementary Data 5
Description: Putative genomic regions of selective sweep in M. domestica from M. sieversii in Kazakhstan

File Name: Supplementary Data 6
Description: List of genes within putative regions of selective sweeps from M. sieversii in Kazakhstan. Differentially expressed genes during fruit development are highlighted

File Name: Supplementary Data 7
Description: Putative genomic regions of selective sweep in M. domestica from M. sylvestris

File Name: Supplementary Data 8
Description: List of genes within putative regions of selective sweeps from M. sylvestris. Differentially expressed genes during fruit development are highlighted

File Name: Supplementary Data 9
Description: Genome-wide scanning of GO enriched genes within selective sweep regions from M. sieversii in Kazakhstan and from M. sylvestris

File Name: Supplementary Data 10
Description: List of nonsynonymous SNPs highly divergent between different species groups (top 1% Fst)

File Name: Supplementary Data 11
Description: List of nonsynonymous SNPs highly divergent between rootstock and scion cultivars (top 1% Fst)

File Name: Supplementary Data 12
Description: Genes within selection sweeps from M. sieversii in Kazakhstan and M. sylvestris that underlie fruit weight QTLs fw1 and fw2

File Name: Supplementary Data 13

Description: Genes within selection sweeps from M. sieversii in Kazakhstan and M. sylvestris that underlie a sorbitol QTL

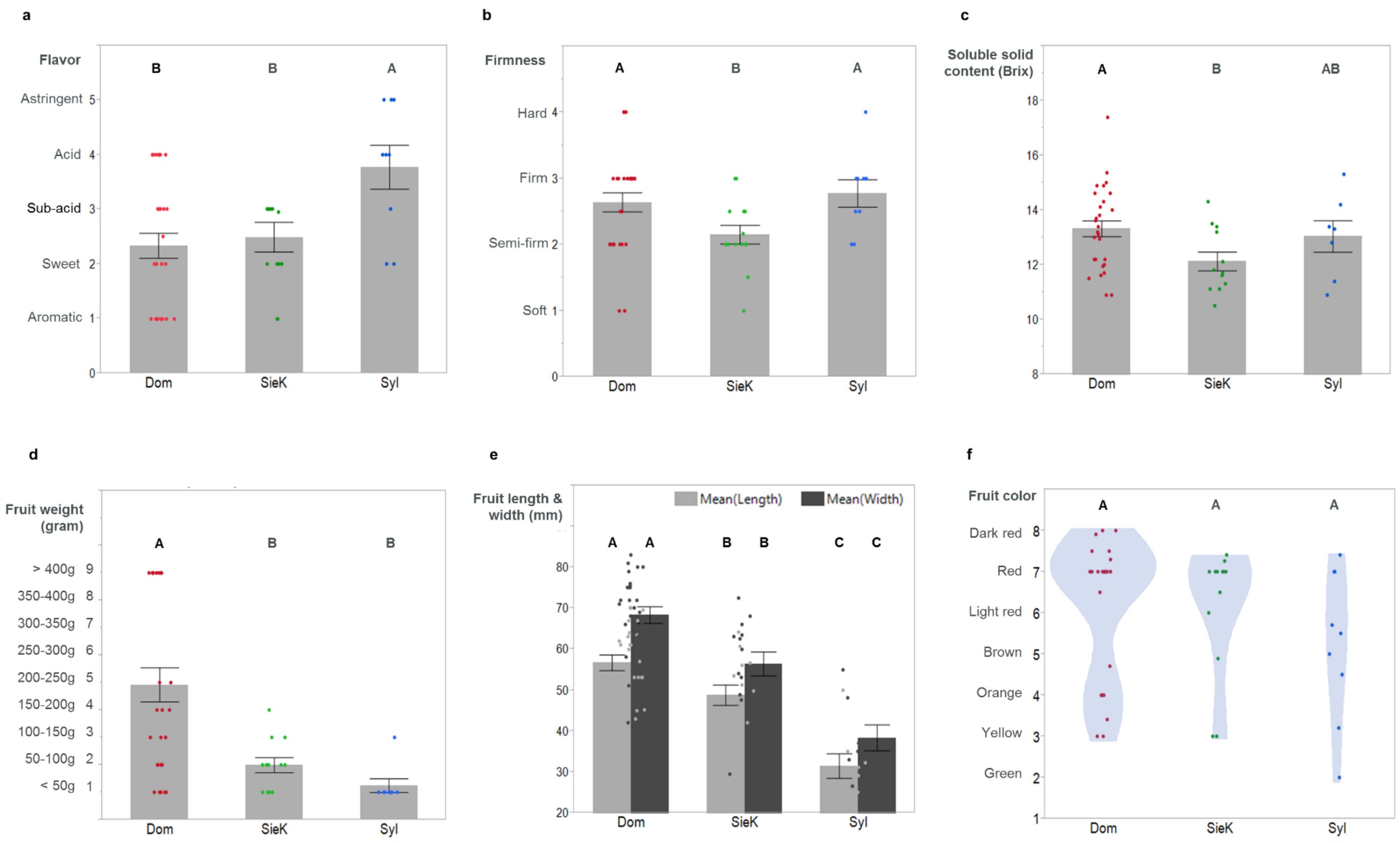

Supplementary Figure 1. Comparative phenotypic analysis of *M. domestica*, *M. sieversii* in Kazakhstan and *M. sylvestris*. Phenotypic data including fruit flavor (**a**), flesh firmness (**b**), soluble solid content (**c**), fruit weight (**d**), fruit length and width (**e**), and fruit skin color (**f**) were collected from the USDA GRIN database (https://npgsweb.ars-grin.gov/gringlobal/cropdetail.aspx?type=descriptor&id=115). Each error bar represents standard error (SE) of more than six replicates. Different letters (A, B and C) represent significant phenotypic differences between different species groups (Tukey's HSD pairwise test, P-value <0.05).

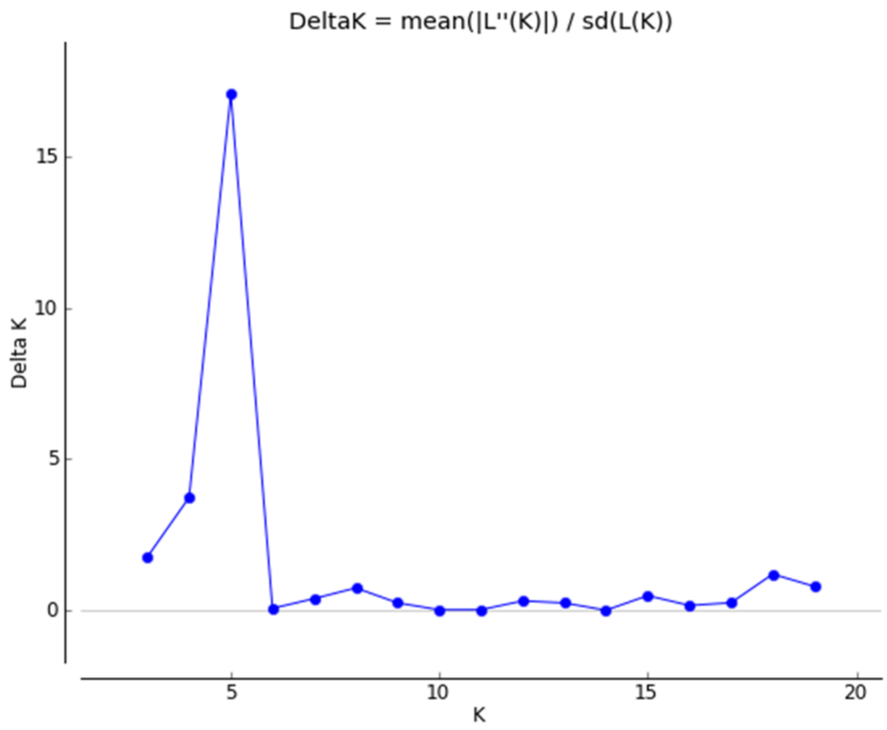

Supplementary Figure 2. Plot of ΔK values with K ranging from 3 to 19 in the population structure analysis using STRUCTURE.

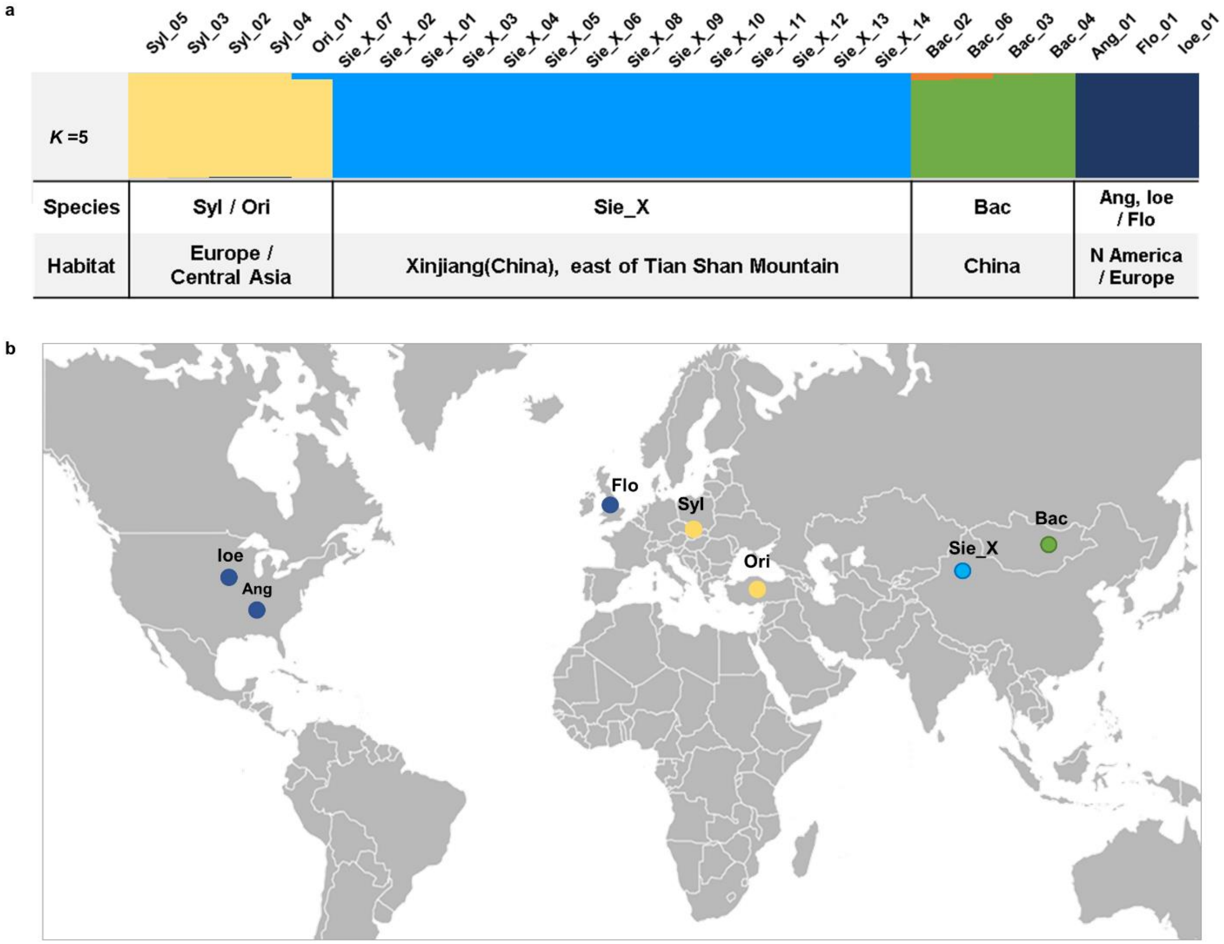

Supplementary Figure 3. Population structure and geographic distribution of species with pure ancestral background. (**a**) Bayesian model-based clustering for *Malus* species with pure ancestral background at *K*=5. Each vertical bar represents one apple accession. Each color represents one putative ancestral background and the bar length quantifies ancestry membership. Species names and their habitats are listed under the structure plot. (**b**) Global map for species with pure ancestral background. Ang, *M. angustifolia*; Bac, *M. baccata*; Flo, *M. florentina*; Hup, *M. hupehensis*; Ioe, *M. ioensis*; Ori, *M. orientalis*; Sie_X, *M. sieversii* in Xinjiang; Syl, *M. sylvestris*.

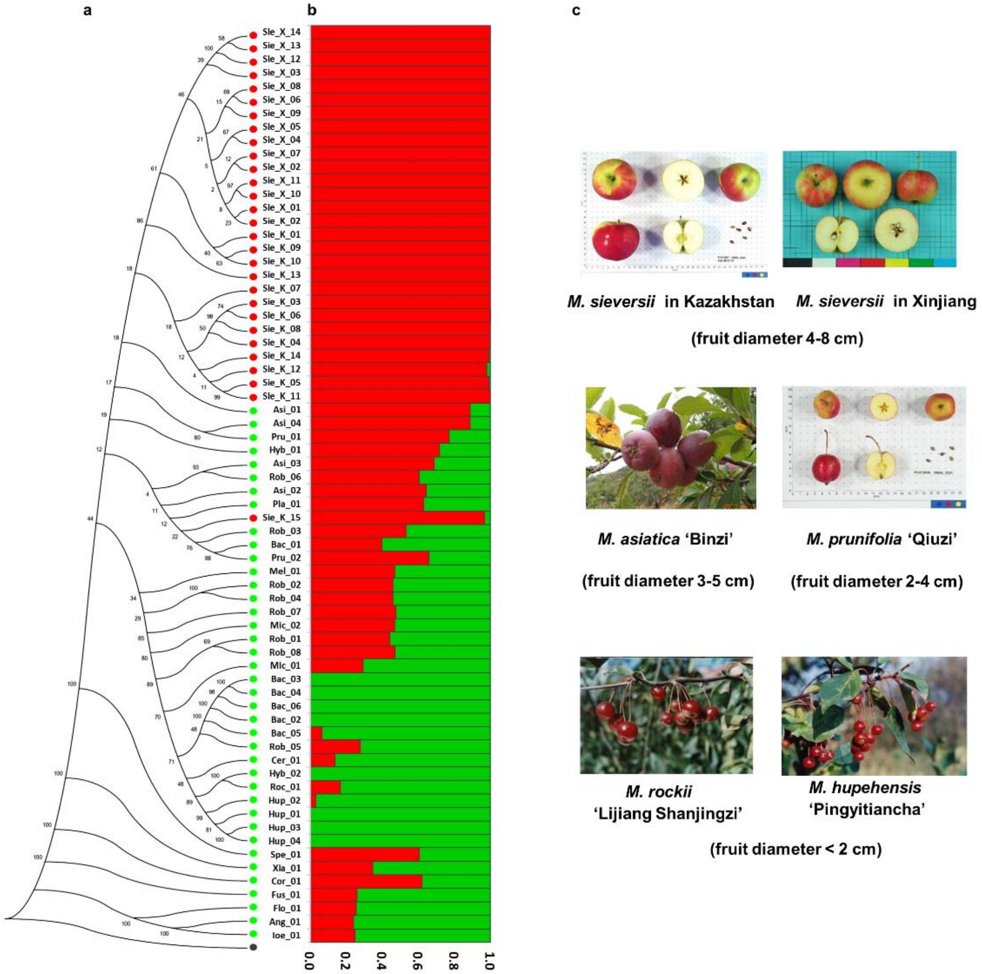

Supplementary Figure 4. Phylogenetic relationship and population structure of *M. sieversii* and other wild species accessions. (**a**) A neighbor-joining phylogenetic tree for *M. sieversii* in Kazakhstan and Xinjiang (red dots) and other wild species (green dots) with pear (black dot) as the outgroup, constructed using 4D SNPs. (**b**) Two ancestral genepool clustering of *M. sieversii* and other wild species accessions. Each row represents one apple accession. Each color represents one sub-population and the bar length in each row quantifies sub-population membership. (**c**) Representative apple fruit pictures with approximated fruit sizes from different phylogenetic clades.

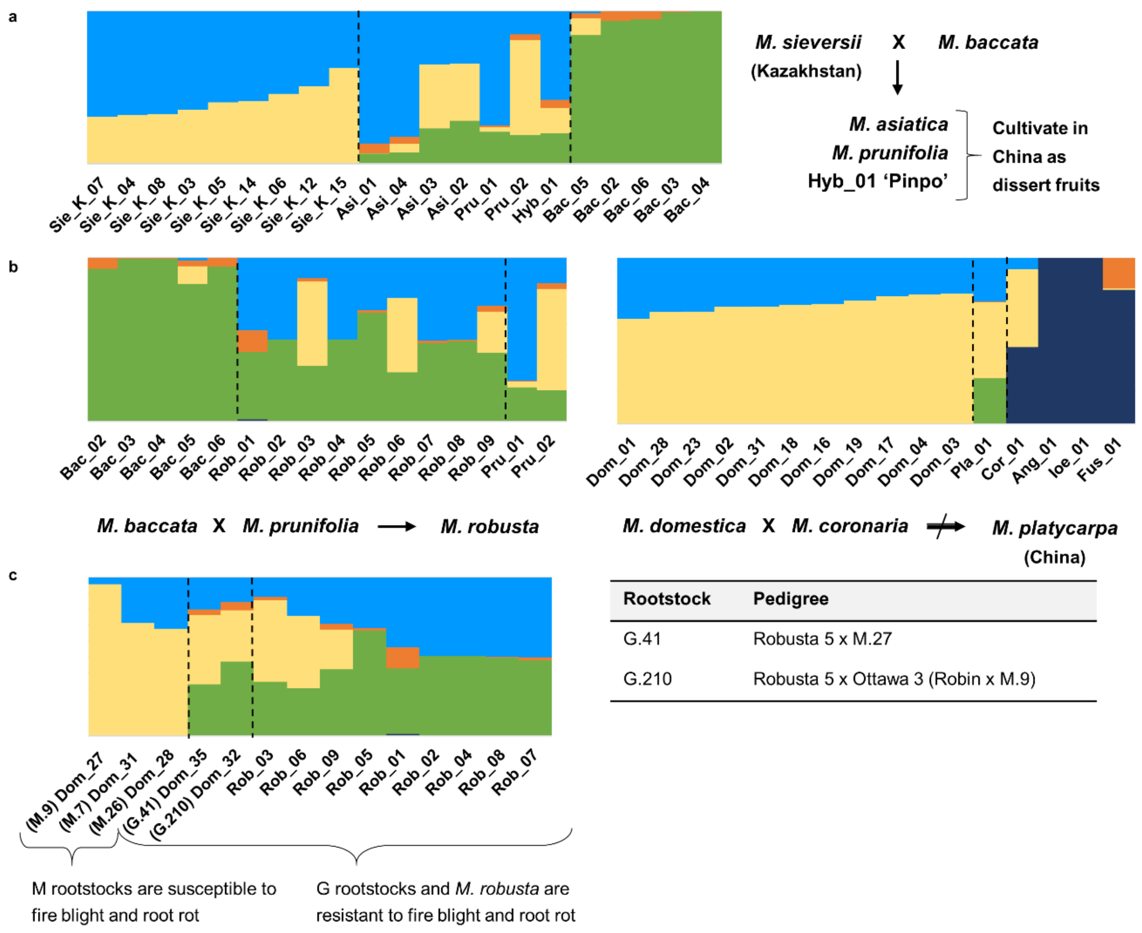

Rootstock	Pedigree
G.41	Robusta 5 x M.27
G.210	Robusta 5 x Ottawa 3 (Robin x M.9)

Supplementary Figure 5. Population structure of hybrid species with admixed ancestral background. Bayesian model-based clustering for *Malus* species with admixed ancestral background at *K*=5. (**a**) Structure analyses support that *M. asiatica*, *M. prunifolia and M. hybrid* are from crosses between *M. sieversii* in Kazakhstan and *M. baccata*. (**b**) Structure analyses support that the hybridization between *M. baccata* and *M. prunifolia* generates *M. robusta*, but do not support the known pedigree of *M. platycarpa*. (**c**) Structure analyses support the known pedigrees of G rootstocks. Each vertical bar represents one apple accession. Each color represents one putative ancestral background and the bar length quantifies ancestry membership.

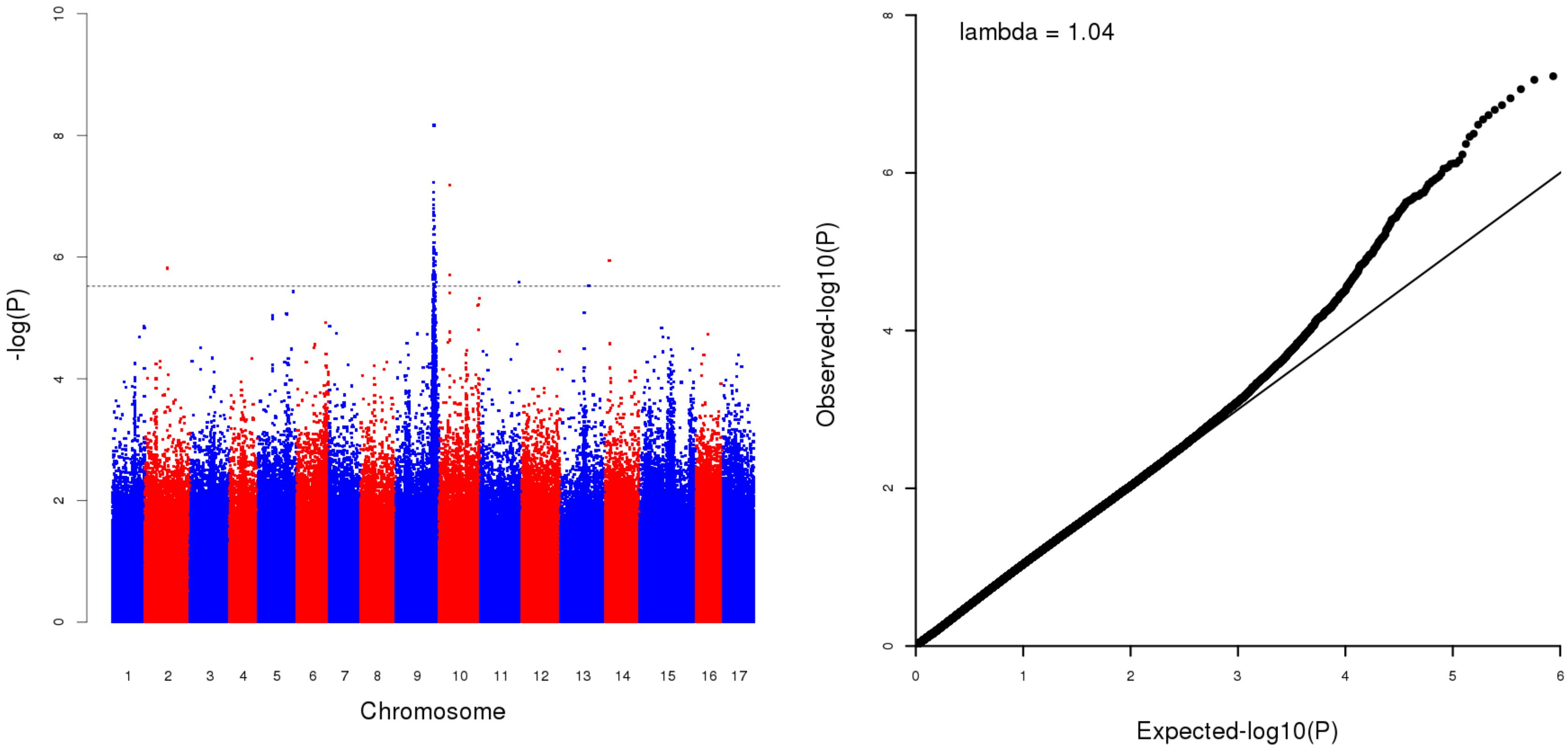

Supplementary Figure 6. GWAS of apple fruit skin color. (**a**) Manhattan plot of GWAS for fruit skin color. The dotted horizontal line indicates the threshold for genome-wide statistical significance (FDR = 0.1; P = 3.03×10^{-6}). (**b**) Q-Q plots of the fruit skin color GWAS results.

	Dom	Sie_K	Sie_X	Syl	Asi	Hup	Bac	Rob
Dom	0	0.11	0.22	0.13	0.14	0.48	0.38	0.16
Sie_K	0.11	0	0.1	0.21	0.11	0.5	0.41	0.16
Sie_X	0.22	0.1	0	0.32	0.16	0.57	0.48	0.21
Syl	0.13	0.21	0.32	0	0.23	0.47	0.38	0.21
Asi	0.14	0.11	0.16	0.23	0	0.45	0.35	0.13
Hup	0.48	0.5	0.57	0.47	0.45	0	0.27	0.37
Bac	0.38	0.41	0.48	0.38	0.35	0.27	0	0.24
Rob	0.16	0.16	0.21	0.21	0.13	0.37	0.24	0

Supplementary Figure 7. Matrix of pairwise F_{ST} values among eight species groups. Asi, *M. asiatica*; Bac, *M. baccata*; Dom, *M. domestica*; Hup, *M. hupehensis*; Rob, *M. robusta*; Sie_K, *M. sieversii* in Kazakhstan; Sie_X, *M. sieversii* in Xinjiang; Syl, *M. sylvestris*.

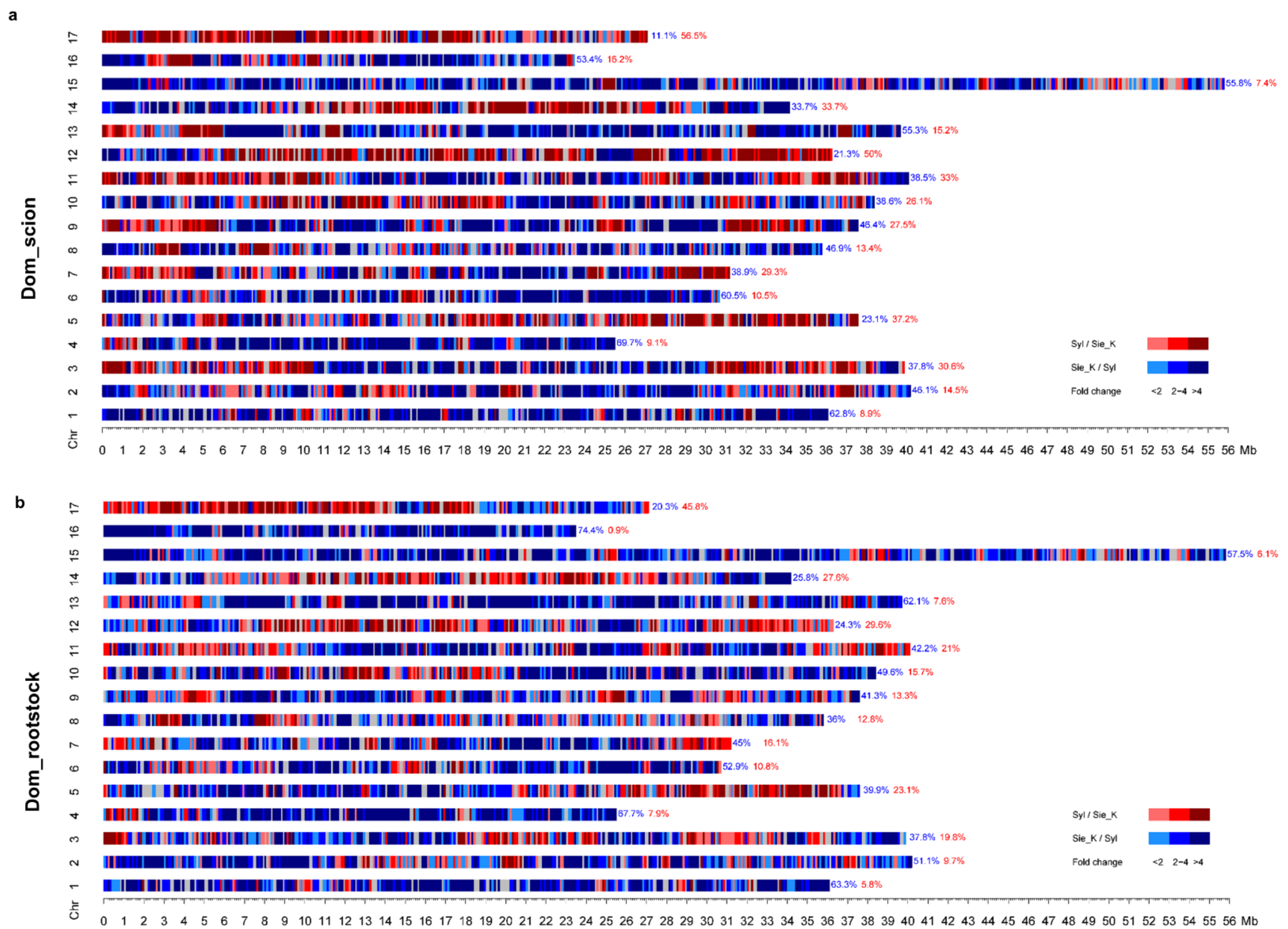

Supplementary Figure 8. Genome-wide distribution of major alleles in scion (**a**) and rootstock (**b**) cultivars of *M. domestica* derived from *M. sieversii* in Kazakhstan (Sie_K in blue) and *M. sylvestris* (Syl in red). For each 100 kb successive window, ratio of alleles derived from the two species was obtained and represented as fold change <2, 2-4 or >4. Gray bars represent undetermined regions. The derived allele percentages from Sie_K and Syl for each chromosome are listed in blue and in red numbers, respectively.

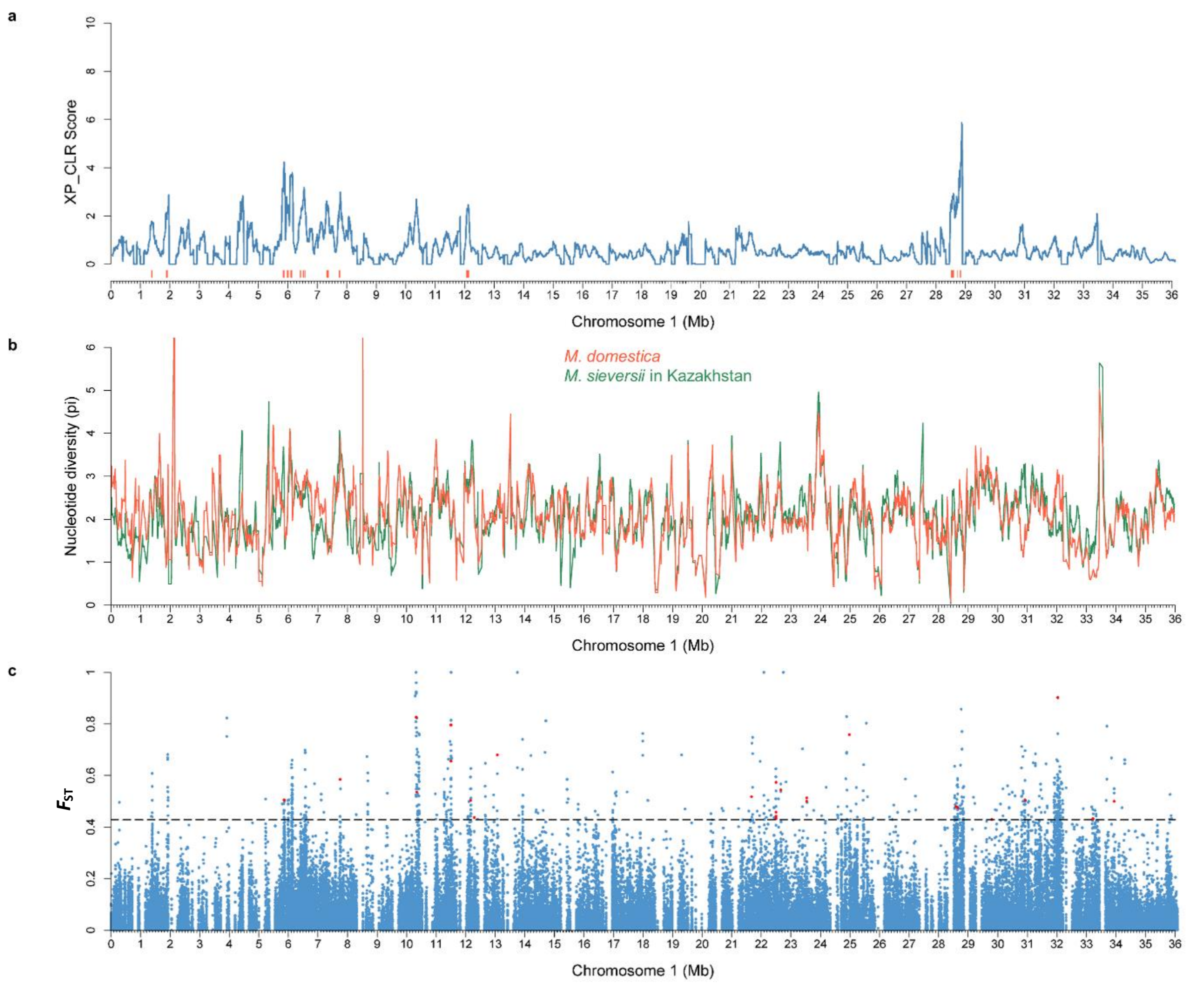

Supplementary Figure 9. Genome-wide distribution of selective sweeps in *M. domestica* from *M. sieversii* in Kazakhstan for each chromosome. (**a**) Distribution of XP_CLR scores for each chromosome with selected regions marked by red bars. (**b**) Distribution of nucleotide diversity (π) in *M. domestica* and *M. sieversii* in Kazakhstan for each chromosome. (**c**) Distribution of F_{ST} values between *M. domestica* and *M. sieversii* in Kazakhstan for each chromosome. Dotted line represents the top 1% F_{ST} value threshold. Nonsynonymous SNPs with top 1% F_{ST} values are marked in red.

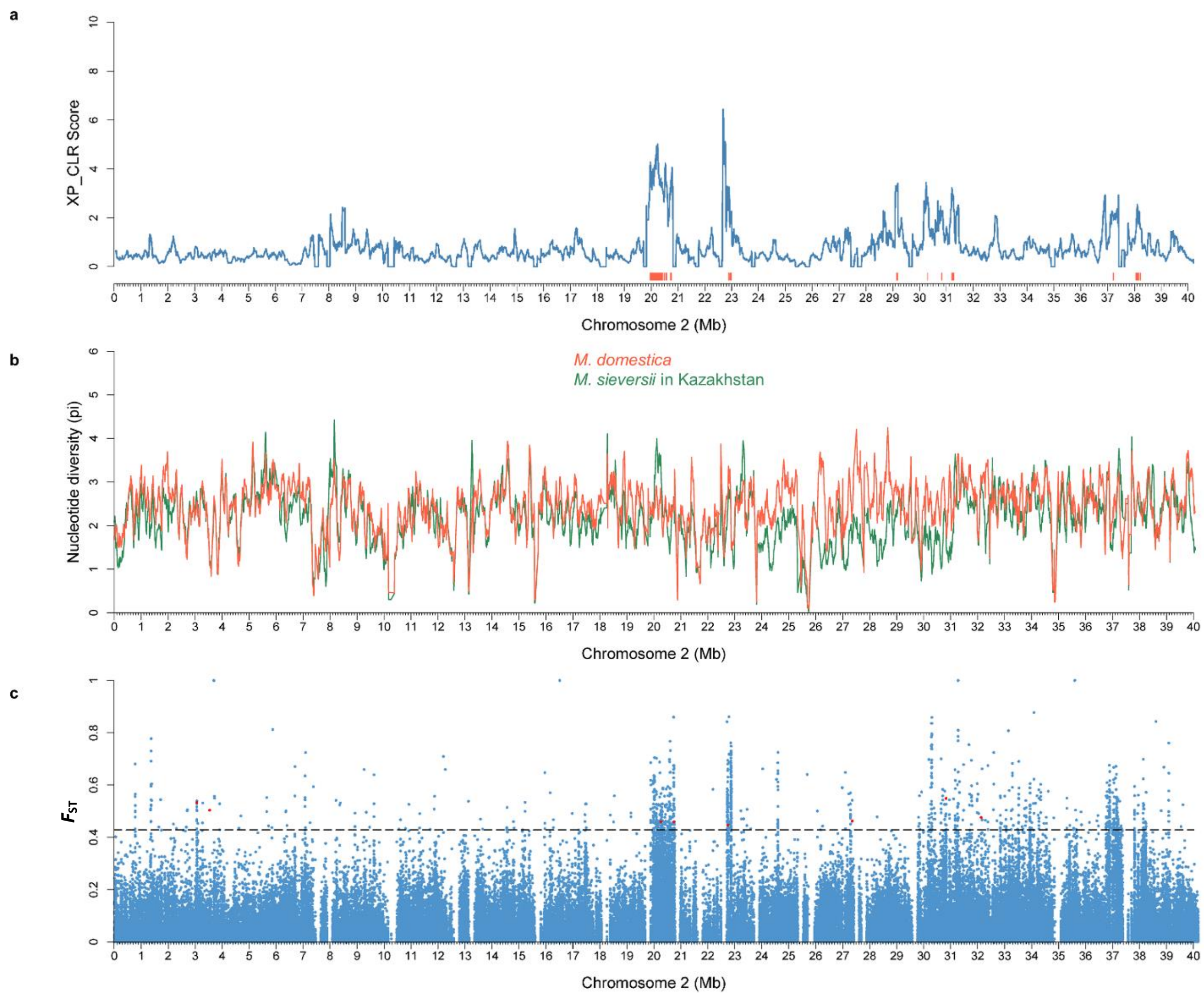

Supplementary Figure 9 (continued)

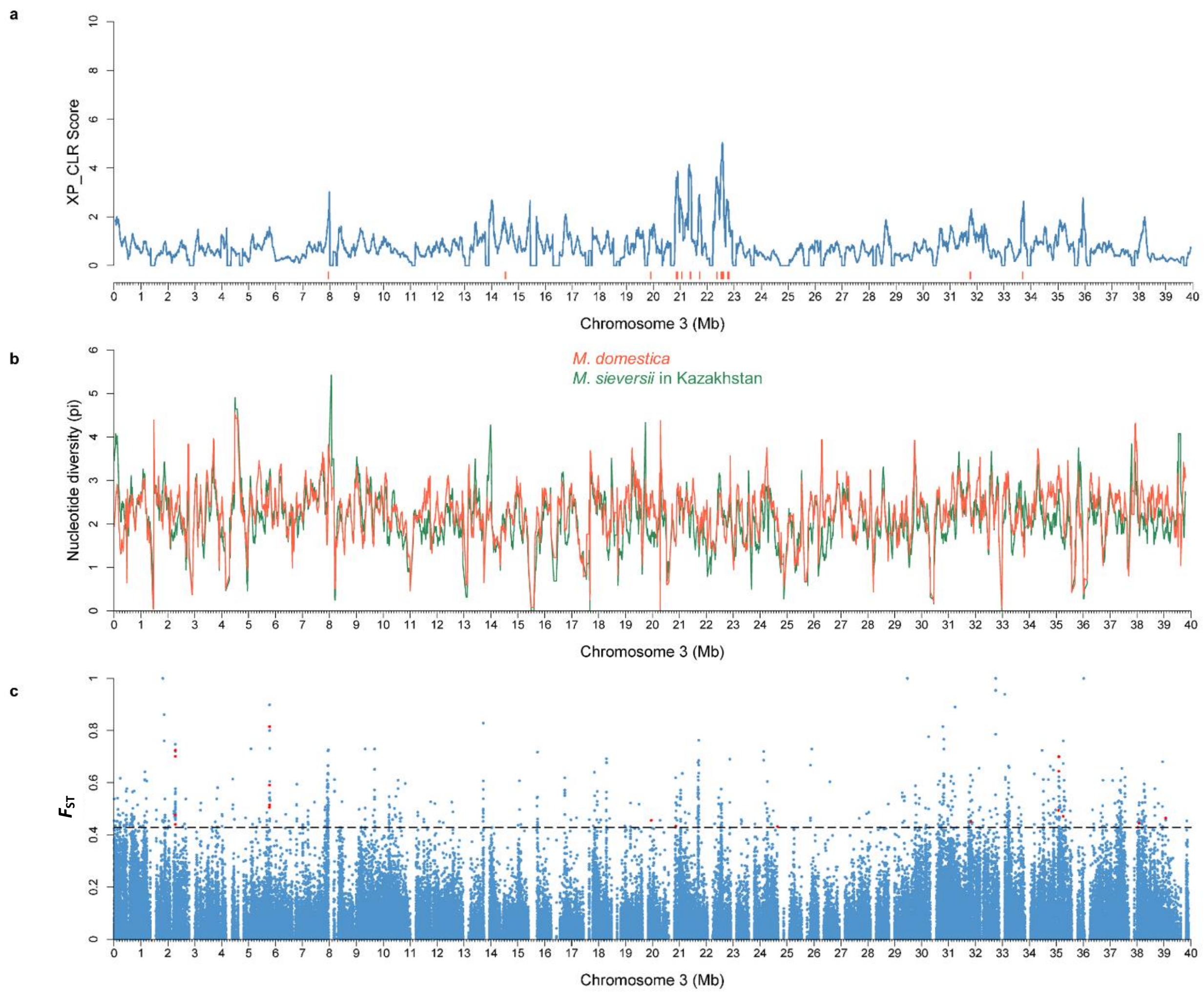

Supplementary Figure 9 (continued)

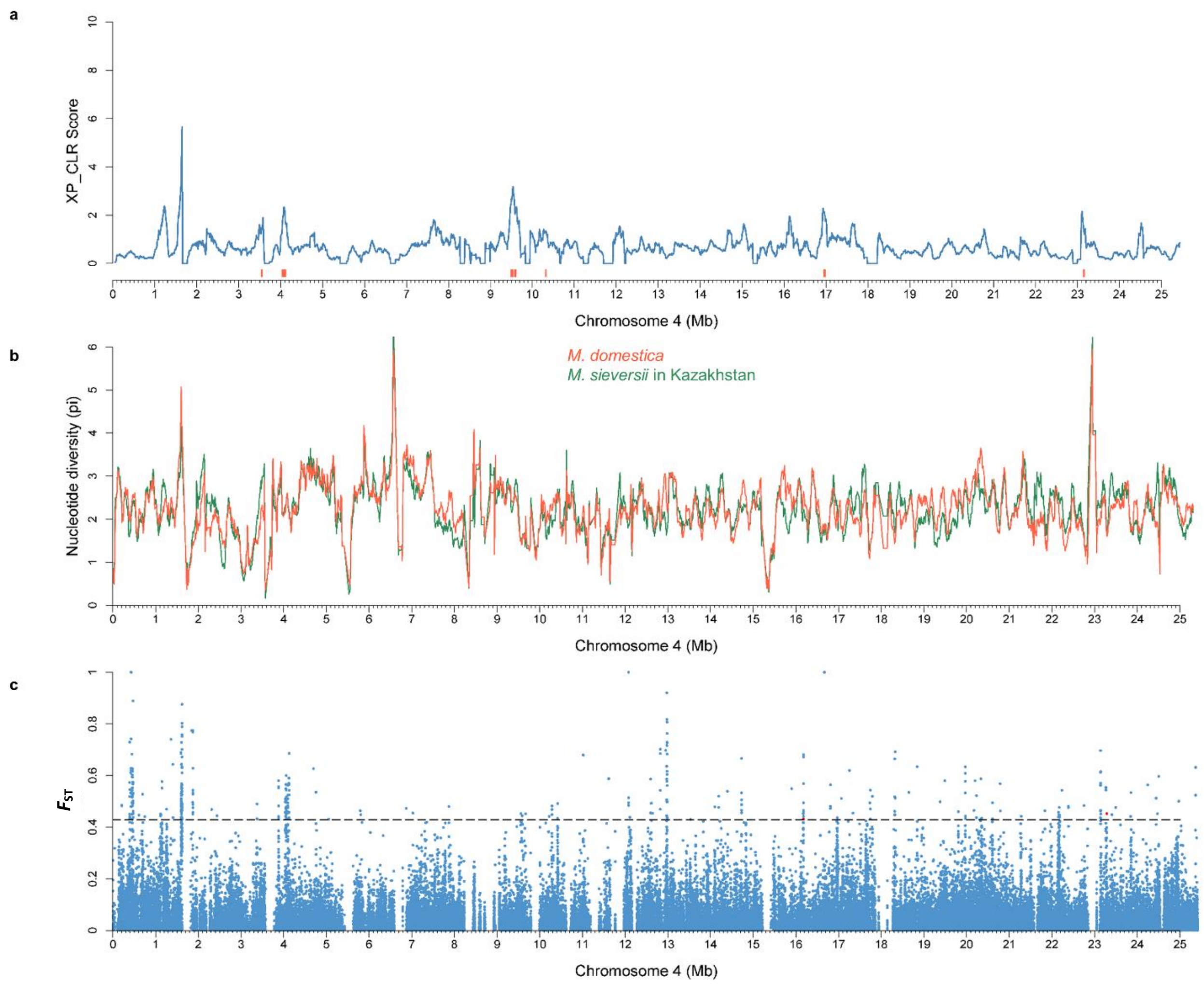

Supplementary Figure 9 (continued)

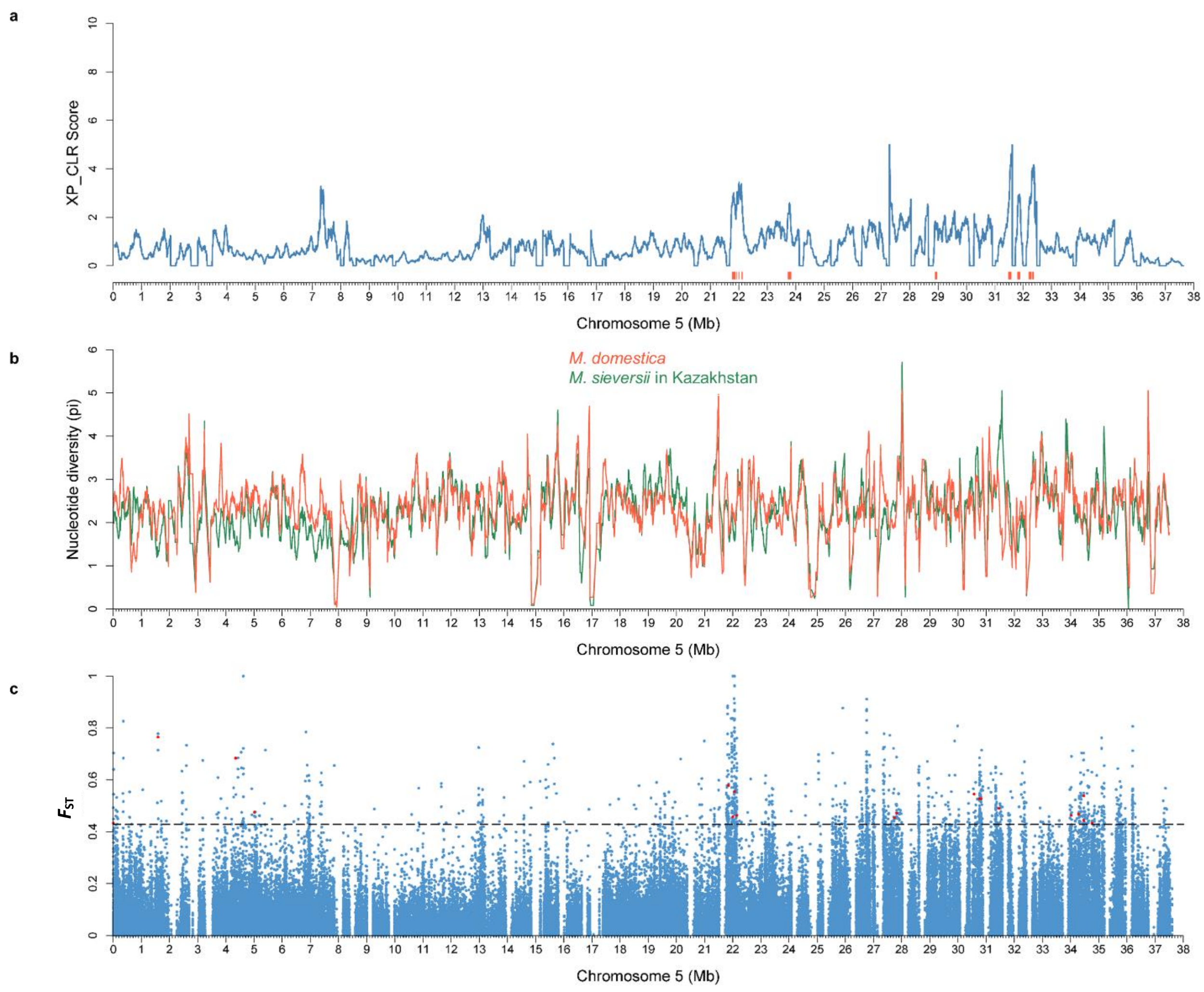

Supplementary Figure 9 (continued)

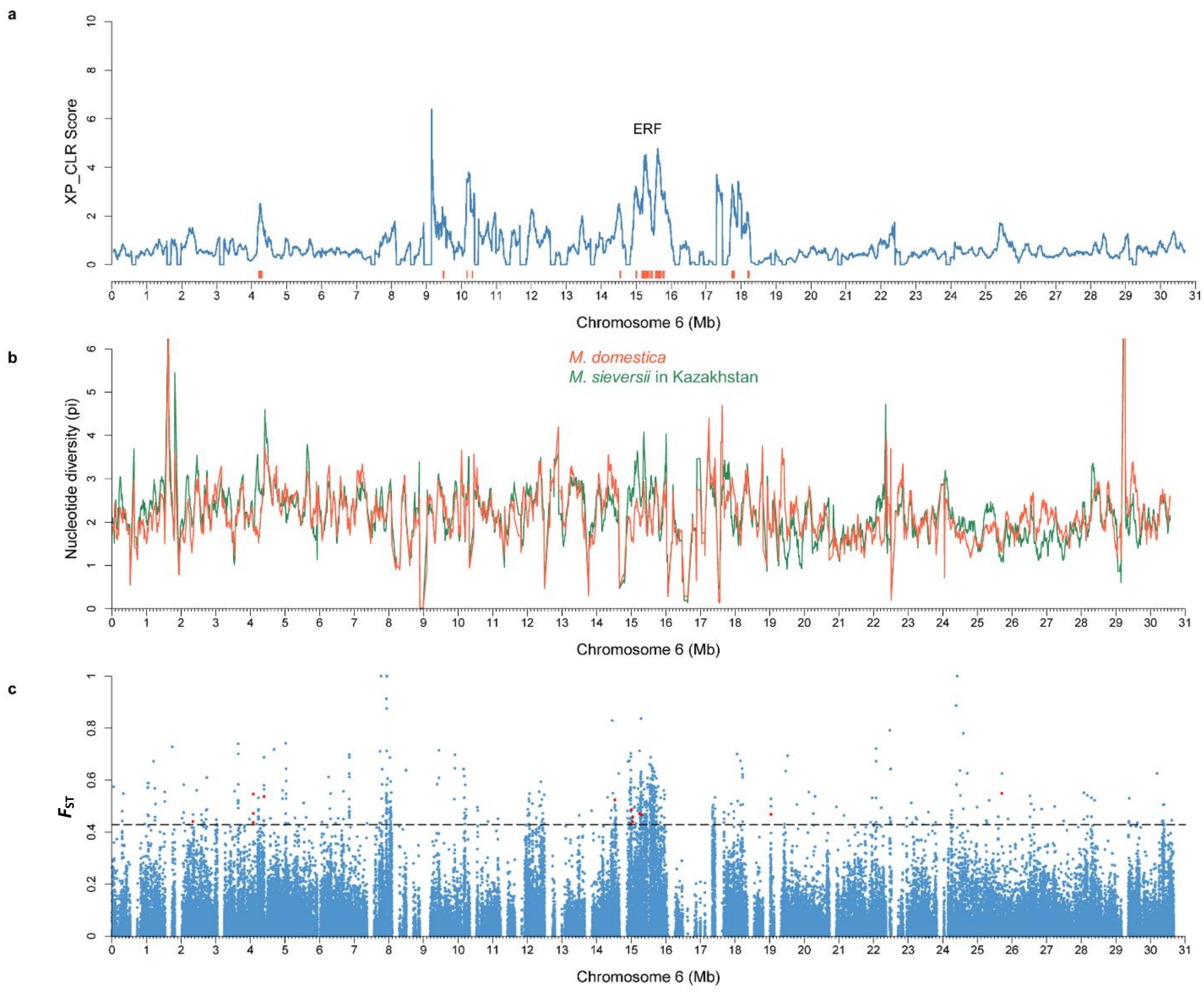

Supplementary Figure 9 (continued)

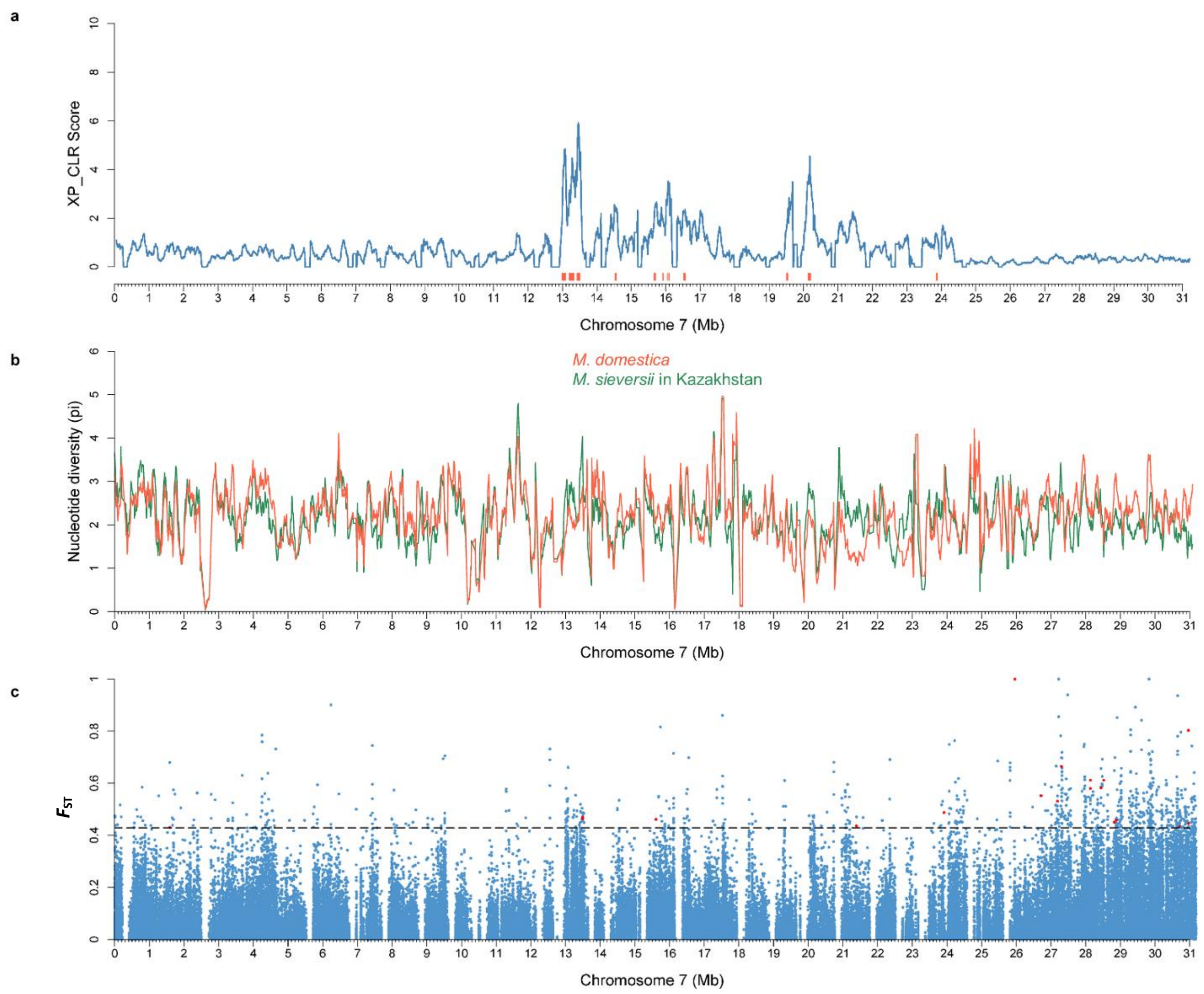

Supplementary Figure 9 (continued)

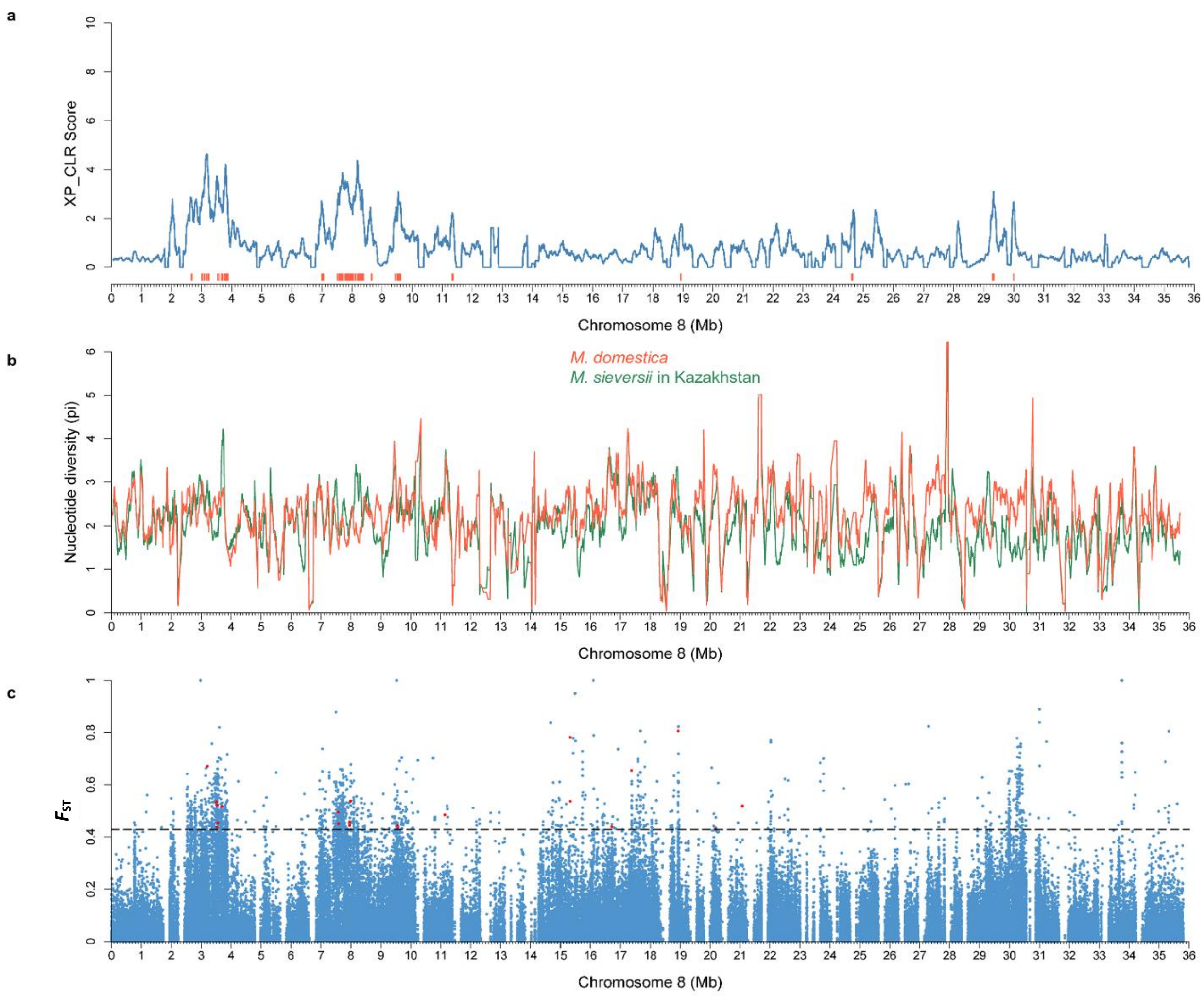

Supplementary Figure 9 (continued)

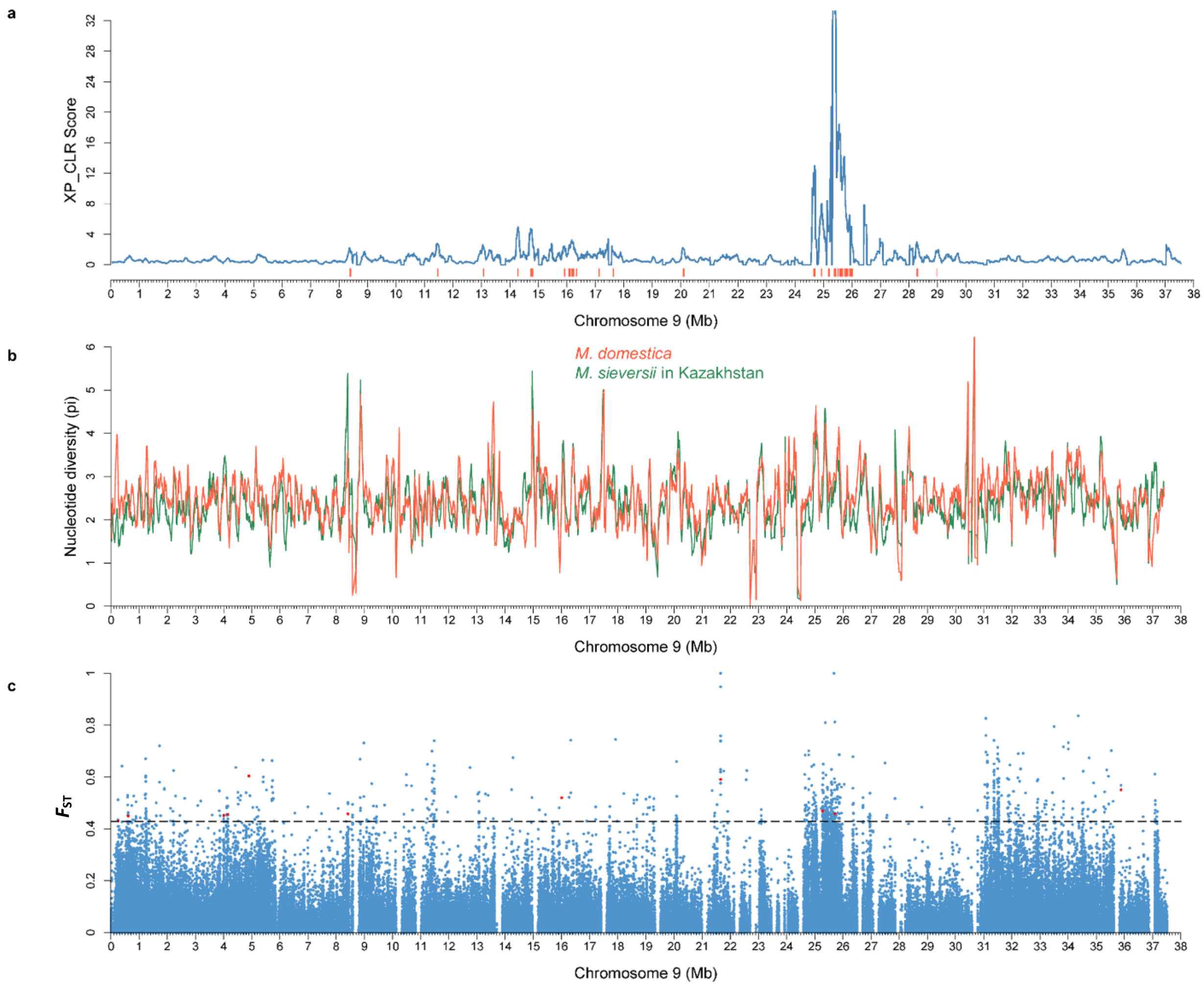

Supplementary Figure 9 (continued)

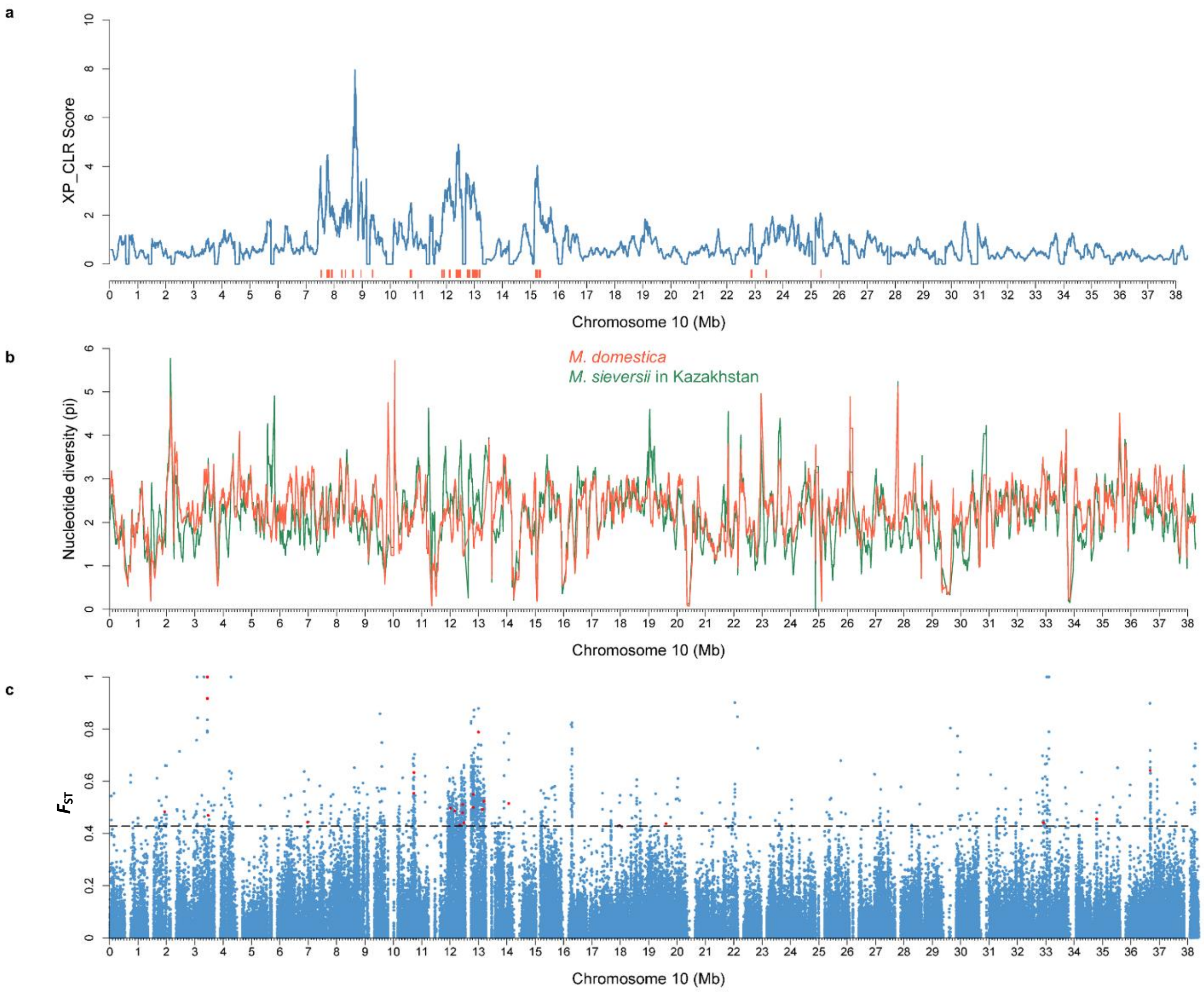

Supplementary Figure 9 (continued)

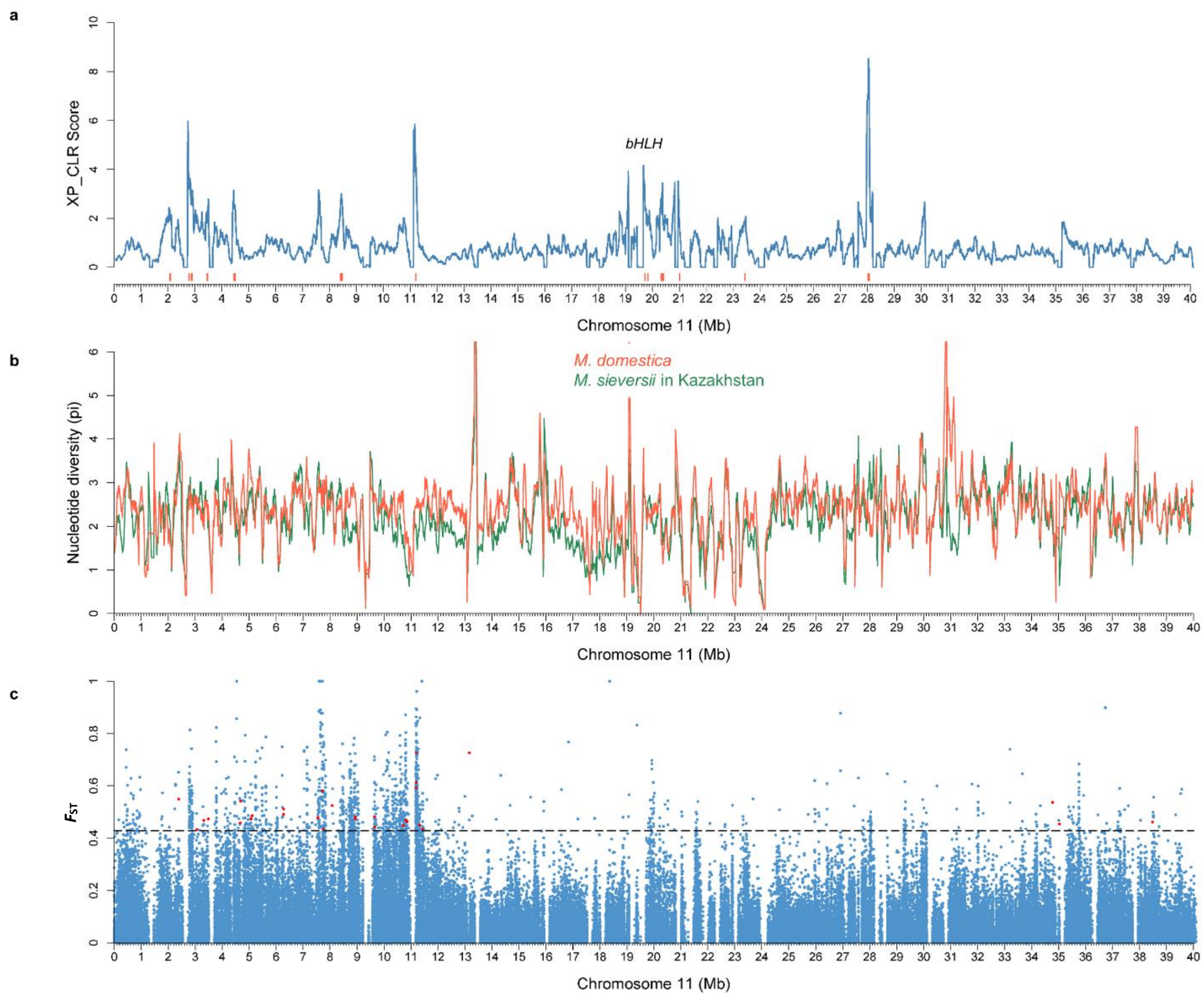

Supplementary Figure 9 (continued)

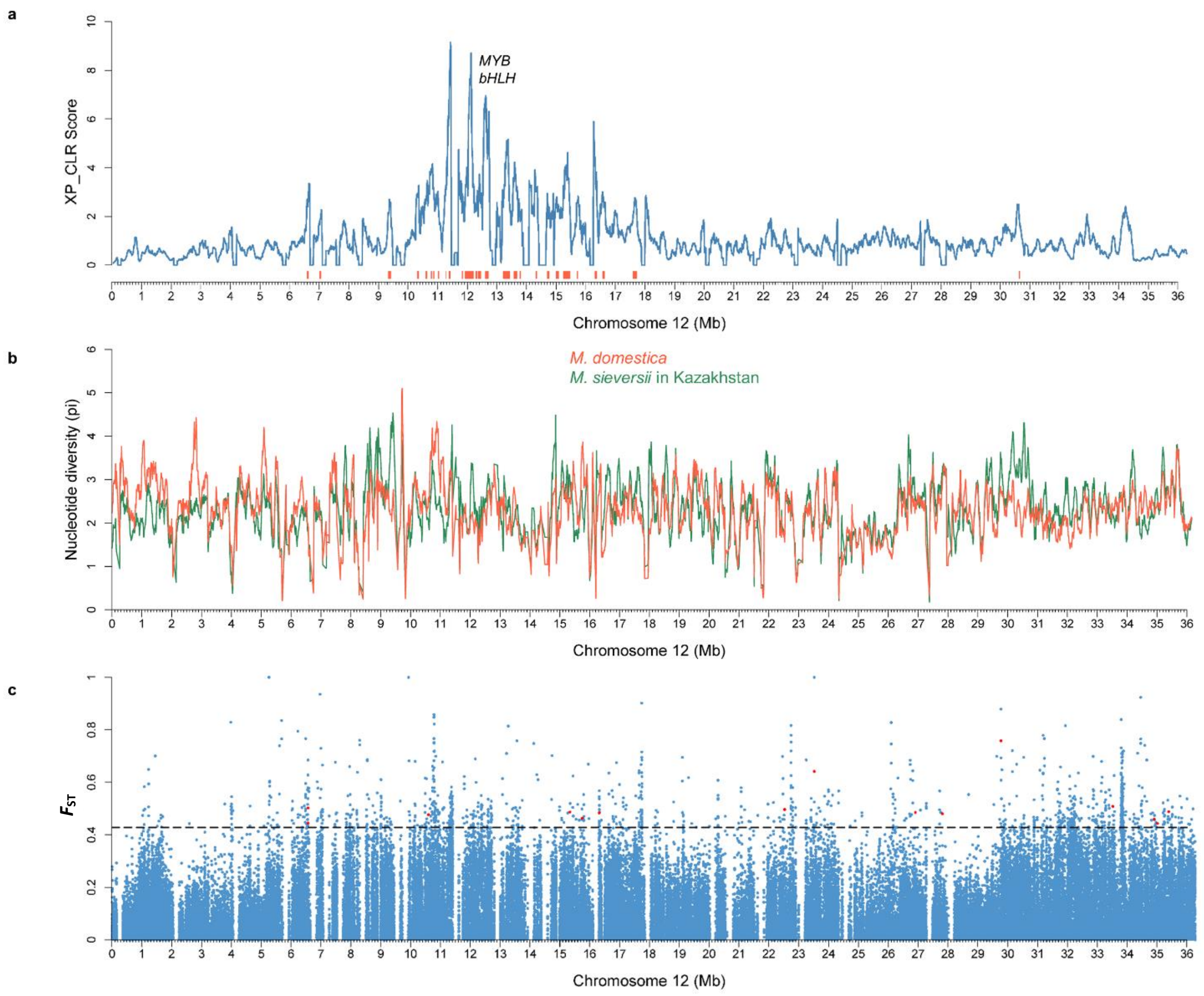

Supplementary Figure 9 (continued)

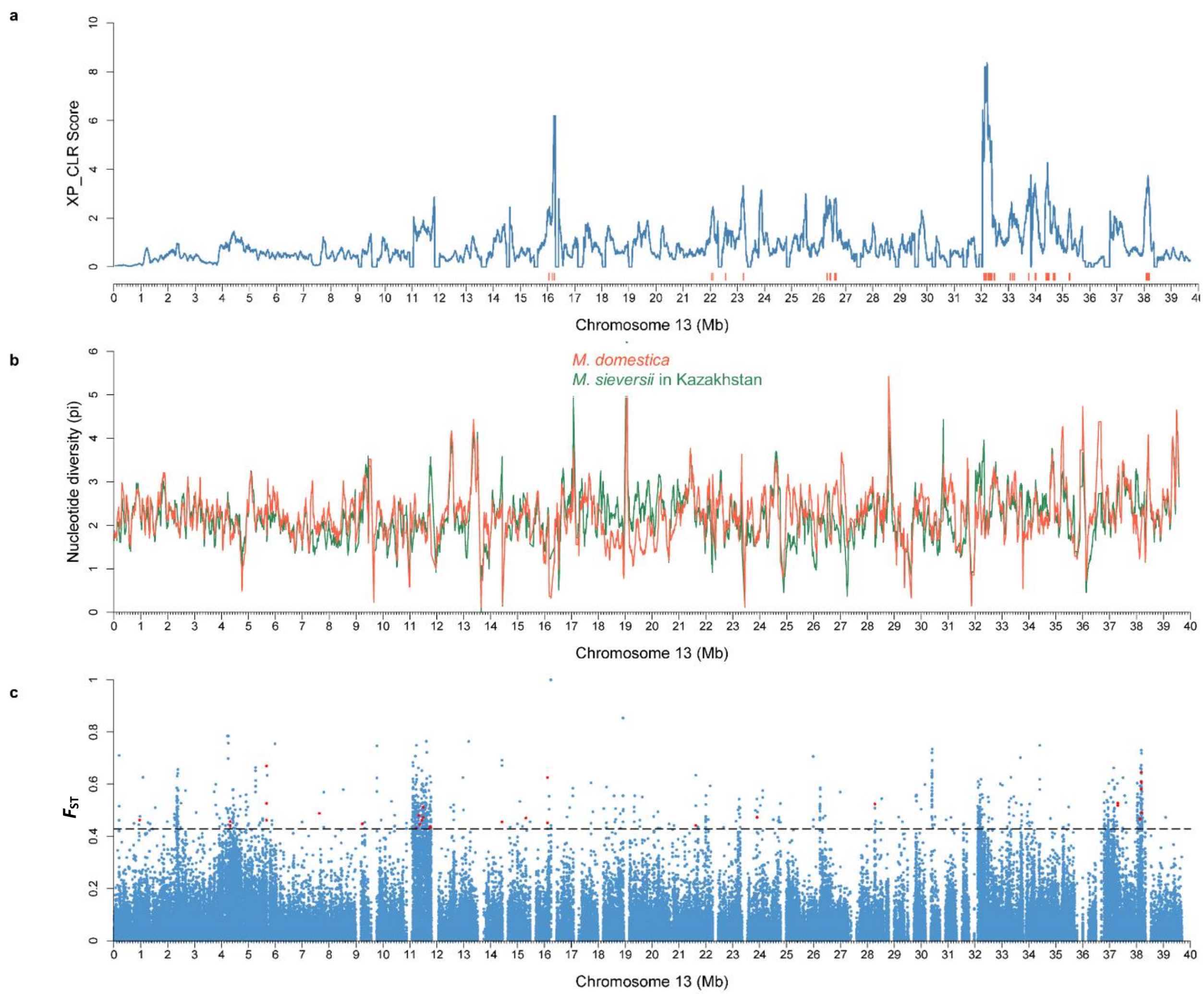

Supplementary Figure 9 (continued)

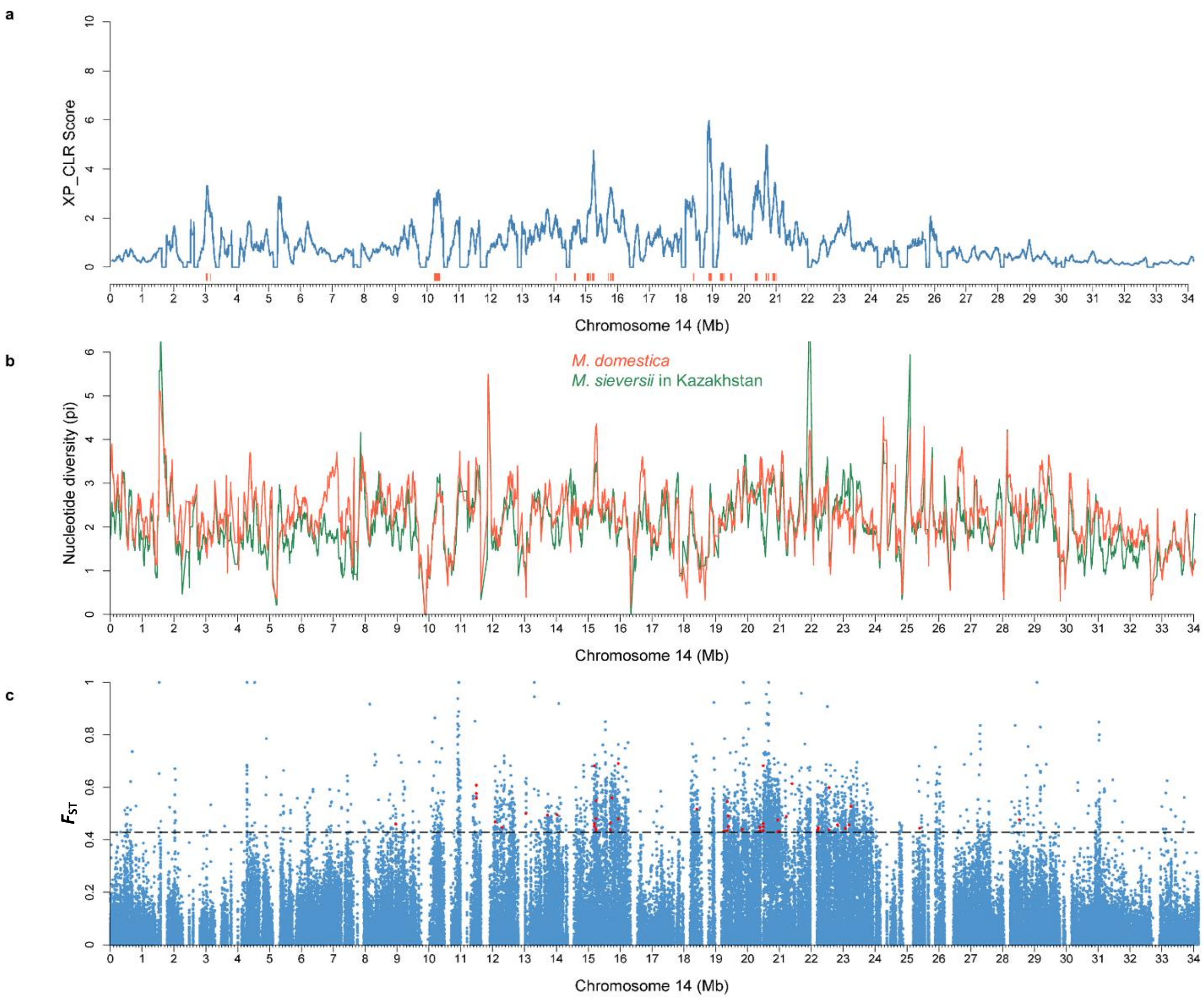

Supplementary Figure 9 (continued)

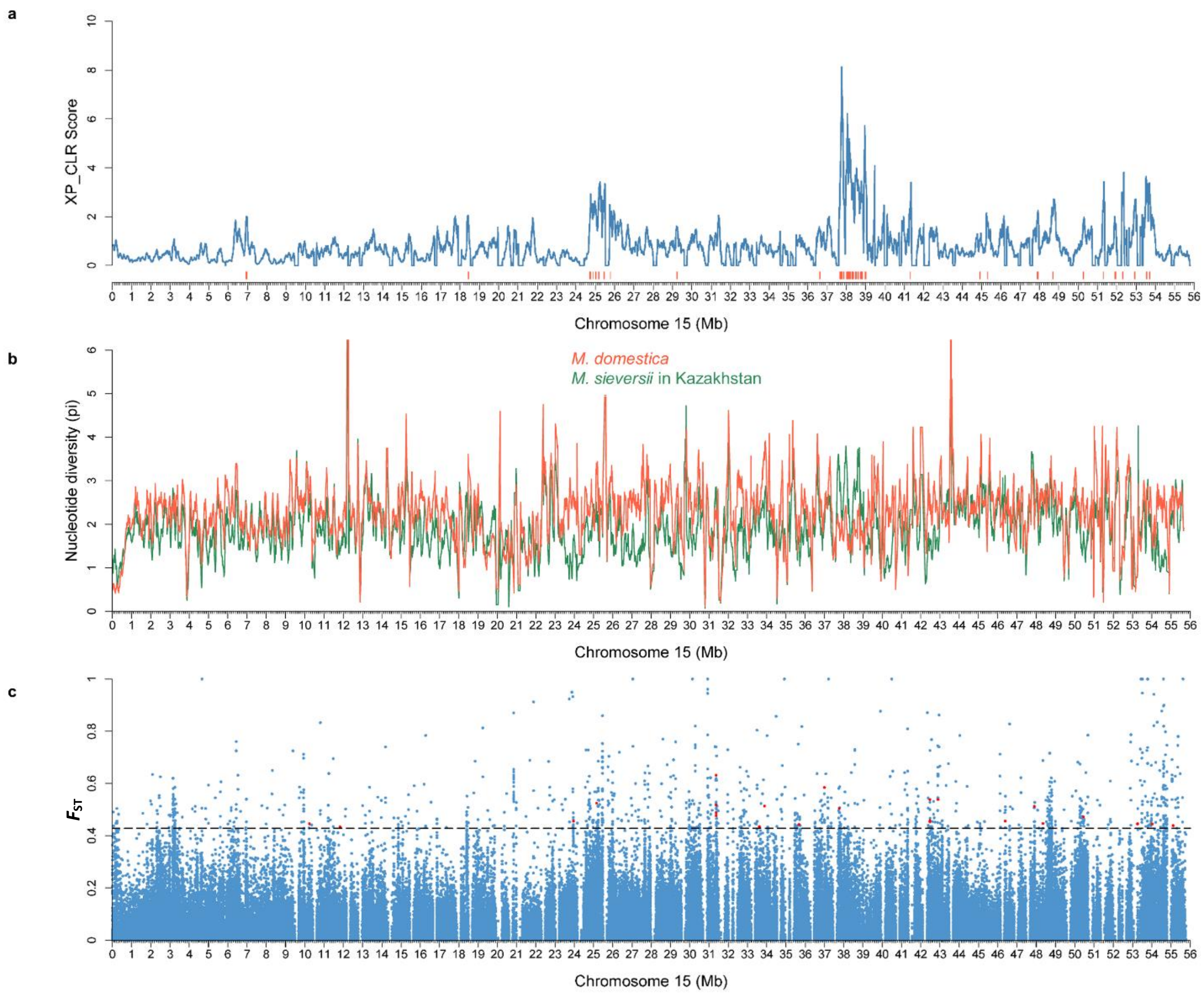

Supplementary Figure 9 (continued)

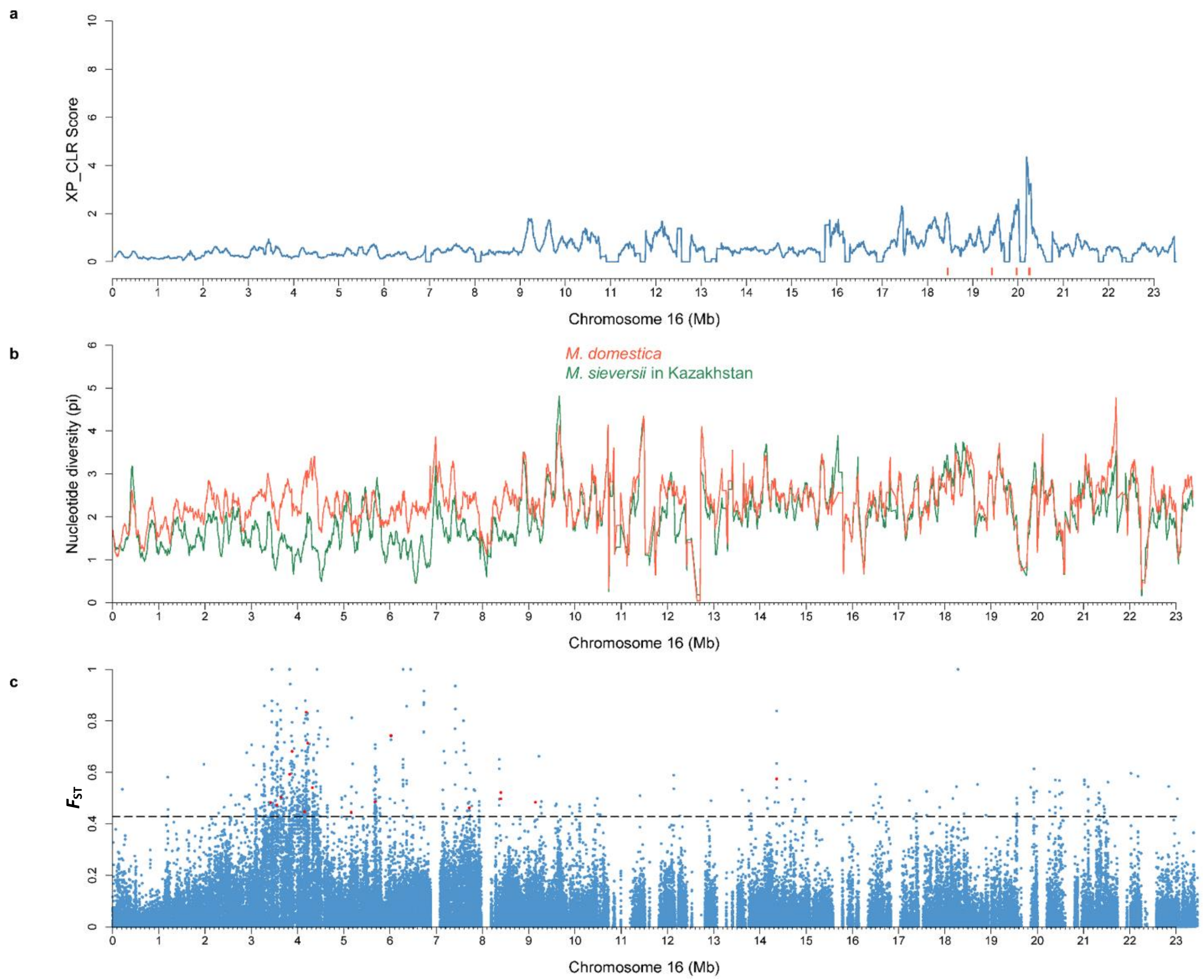

Supplementary Figure 9 (continued)

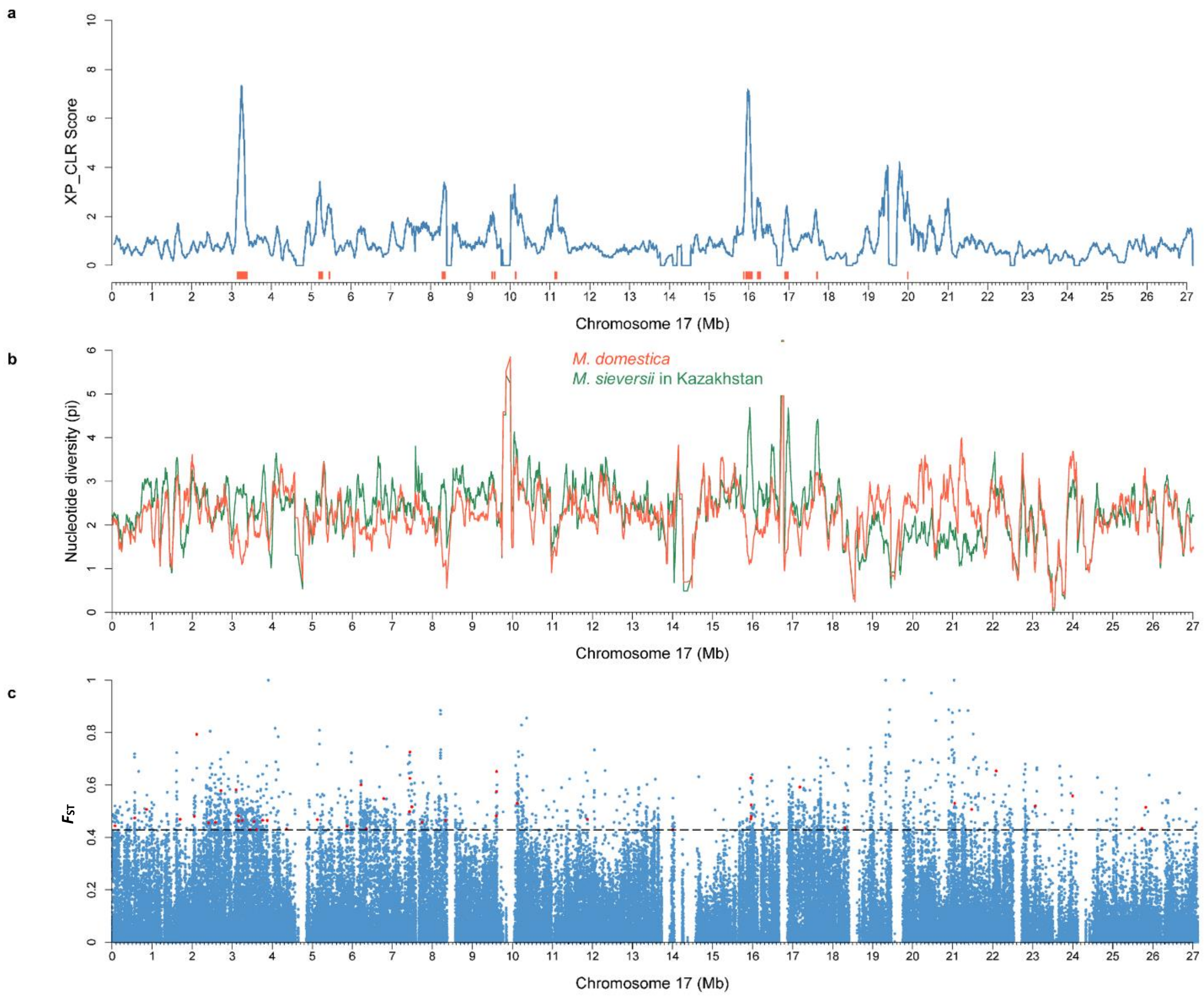

Supplementary Figure 9 (continued)

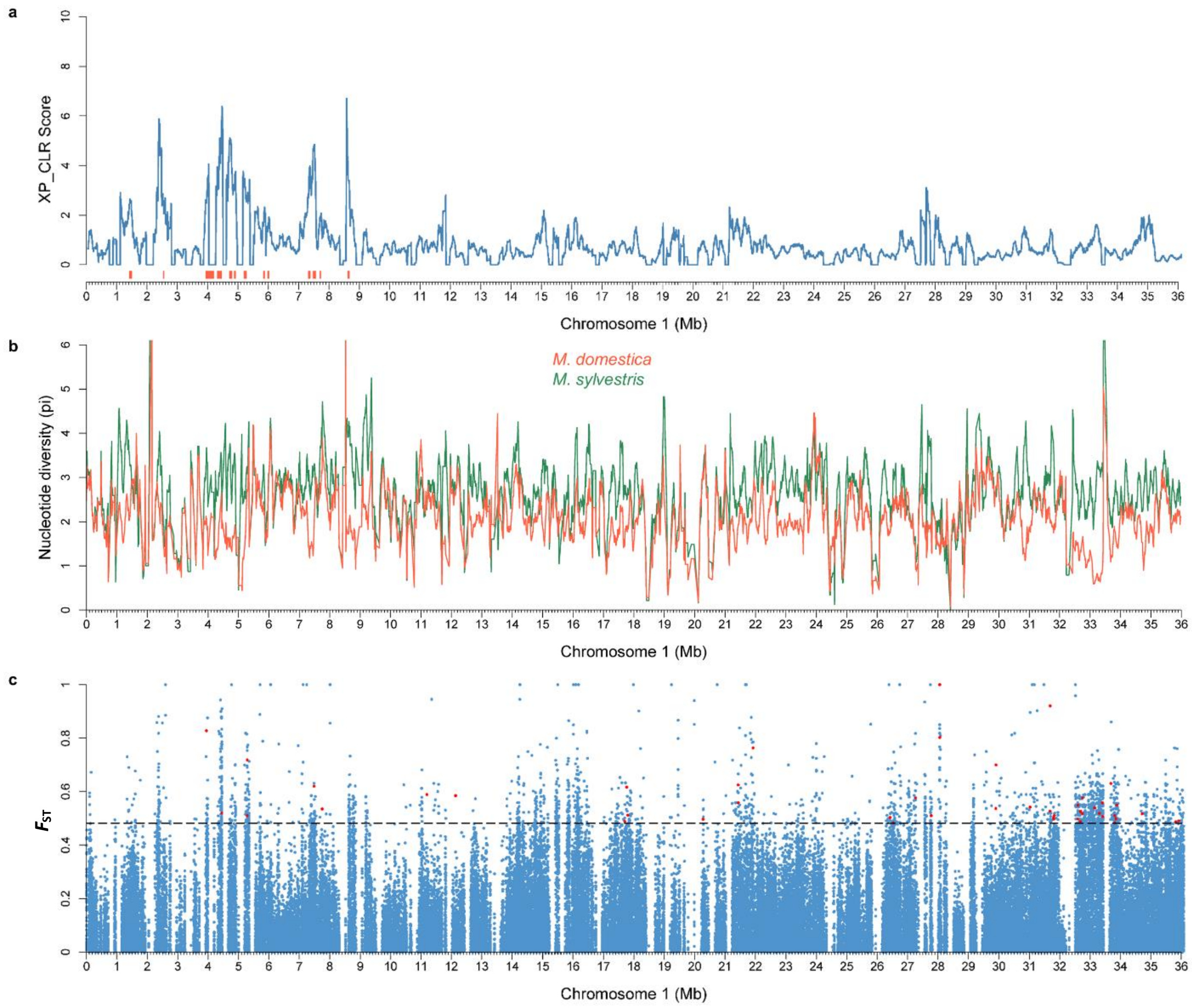

Supplementary Figure 10 Genome-wide distribution of selective sweeps in *M. domestica* from *M. sylvestris* for each chromosome. (**a**) Distribution of XP_CLR scores for each chromosome with selected regions marked by red bars. (**b**) Distribution of nucleotide diversity (π) in *M. domestica* and *M. sylvestris* for each chromosome. (**c**) Distribution of F_{ST} values between *M. domestica* and *M. sylvestris* for each chromosome. Dotted line represents the top 1% F_{ST} value threshold. Nonsynonymous SNPs with top 1% F_{ST} values are marked in red.

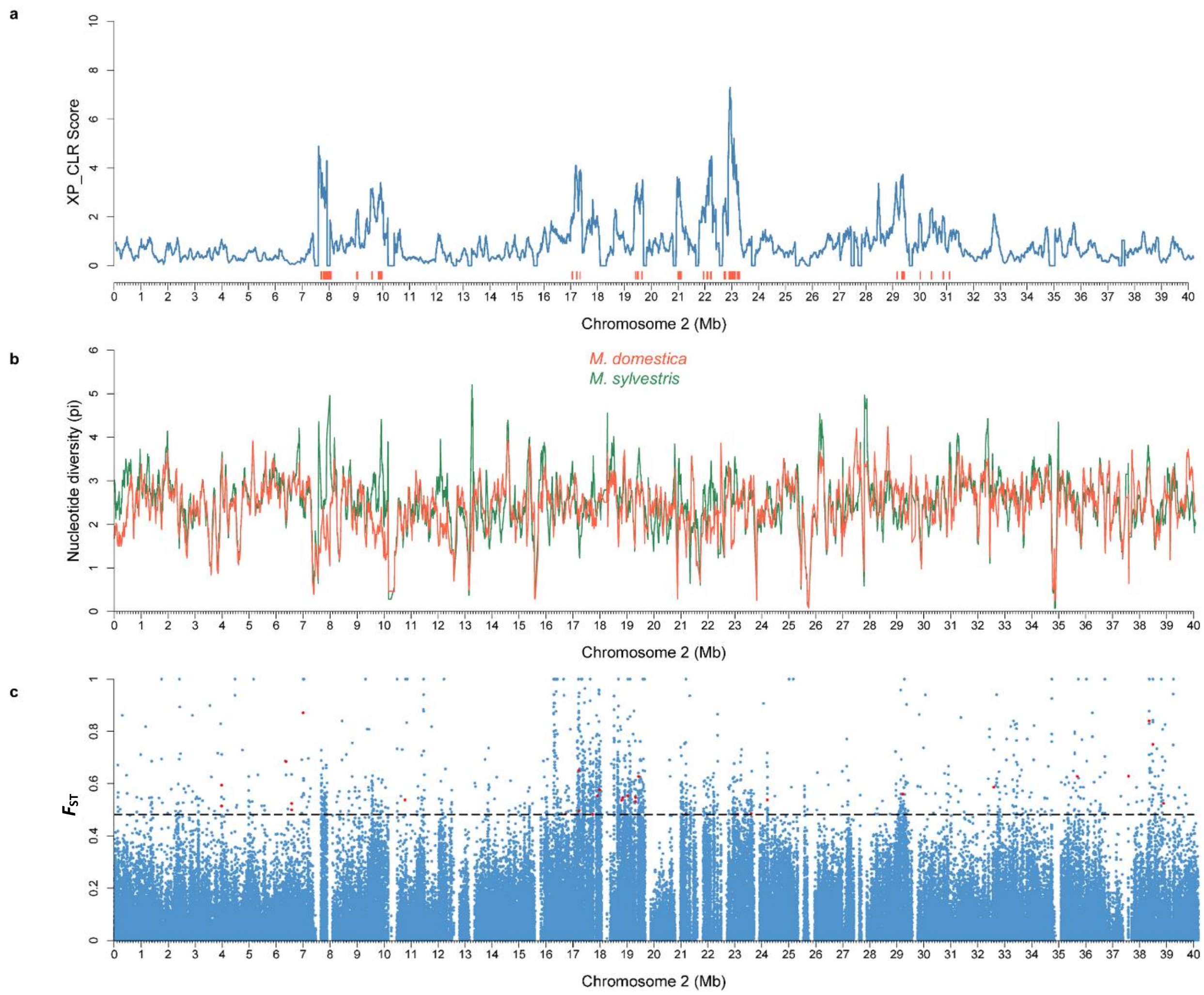

Supplementary Figure 10 (continued)

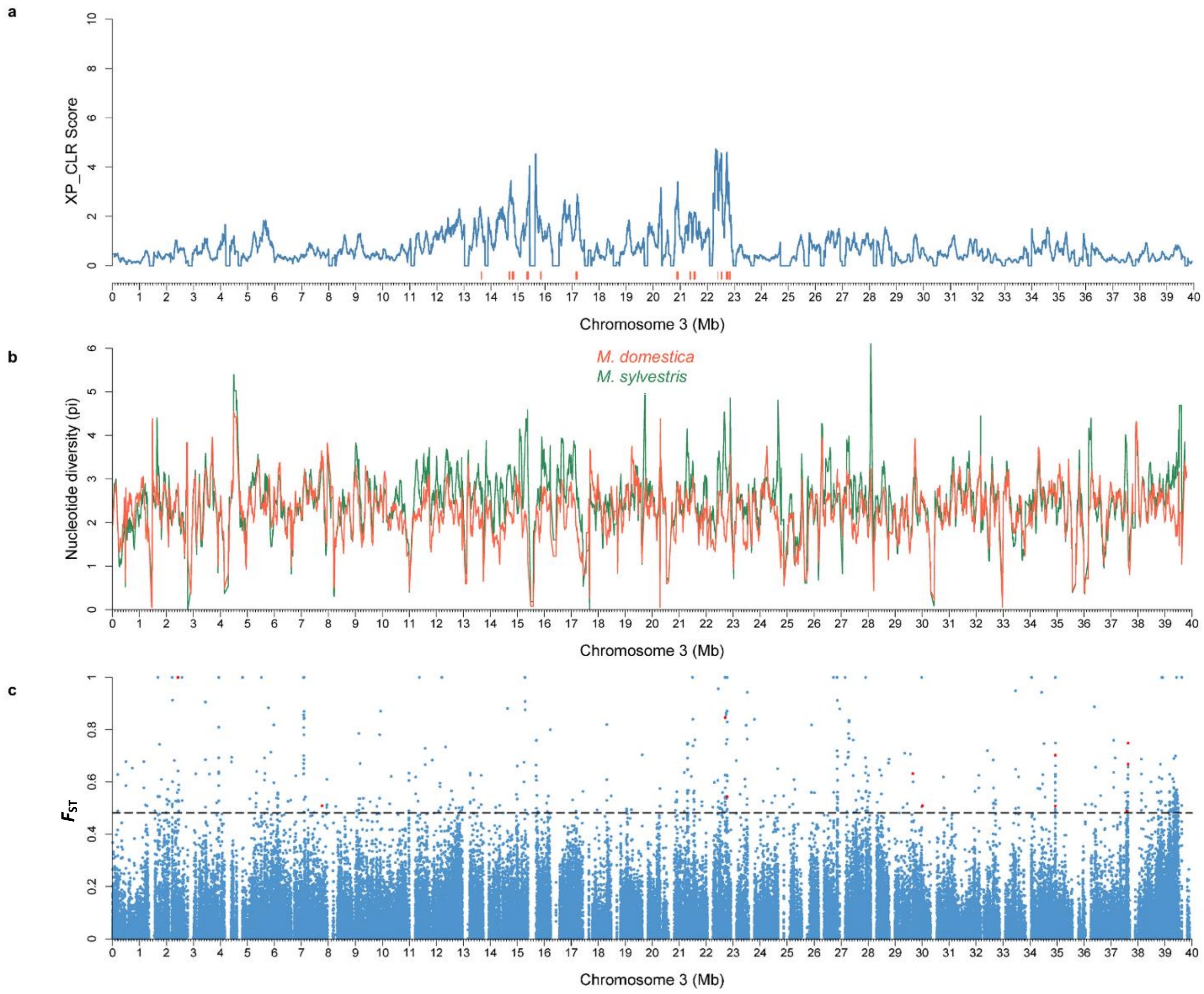

Supplementary Figure 10 (continued)

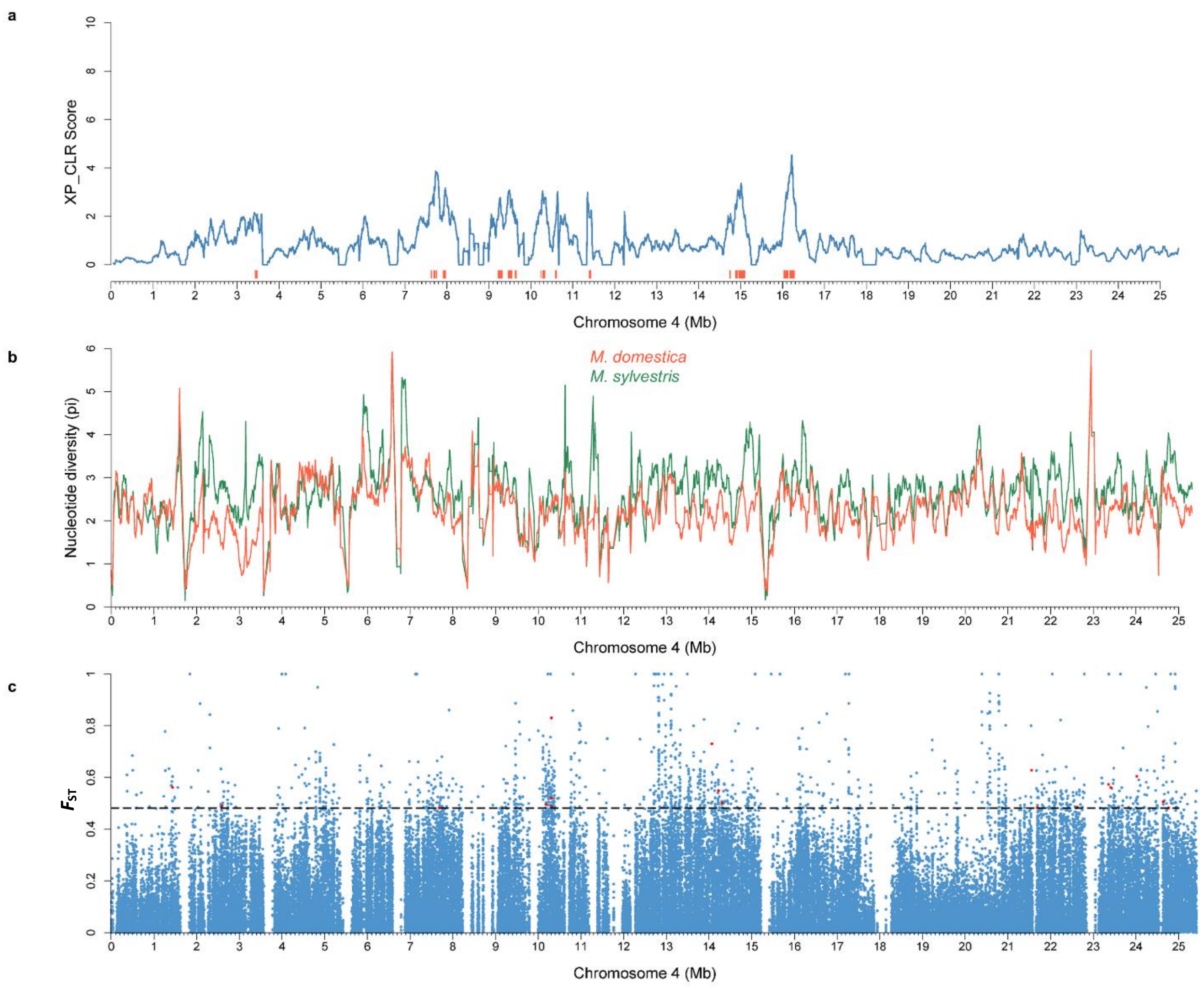

Supplementary Figure 10 (continued)

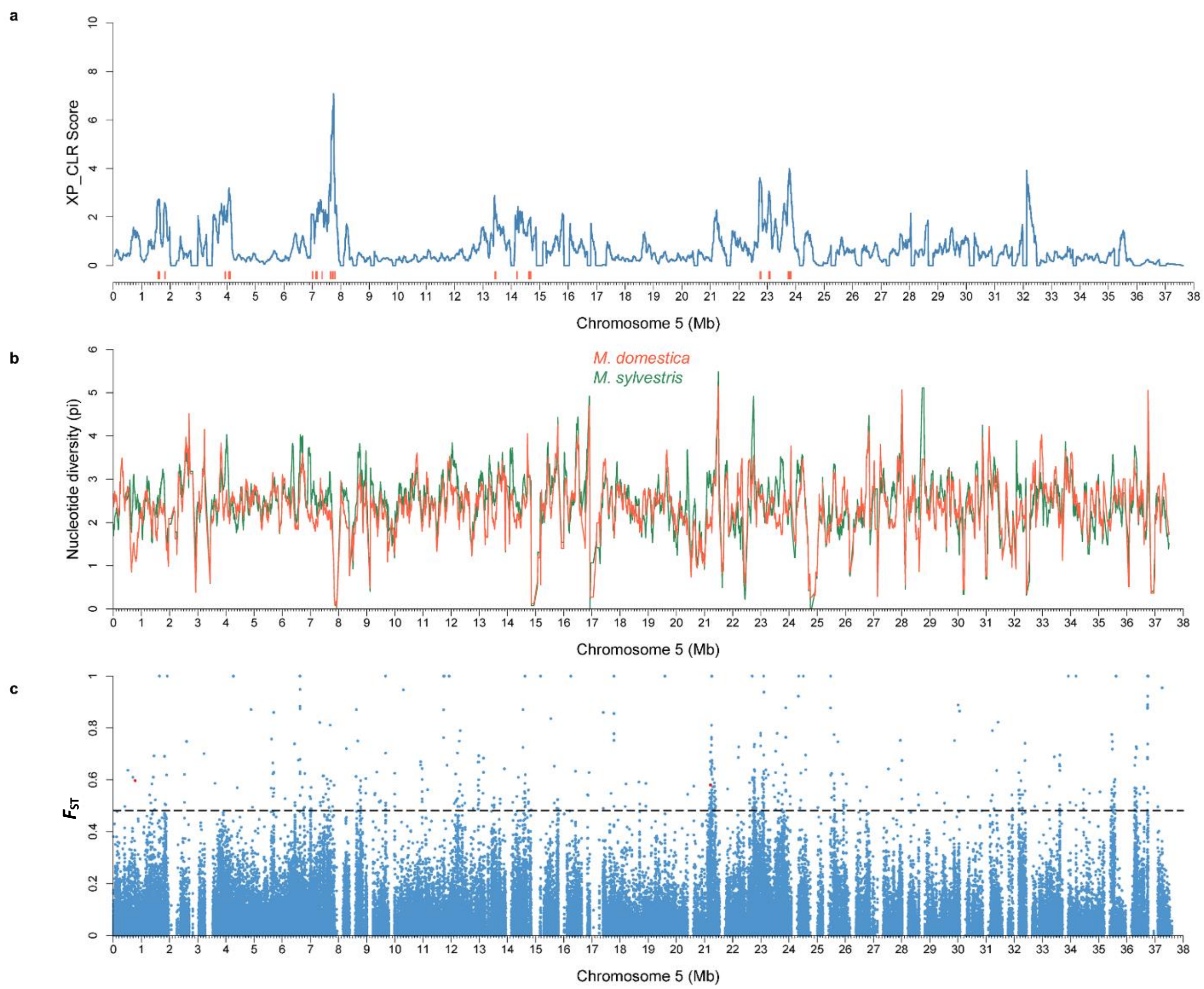

Supplementary Figure 10 (continued)

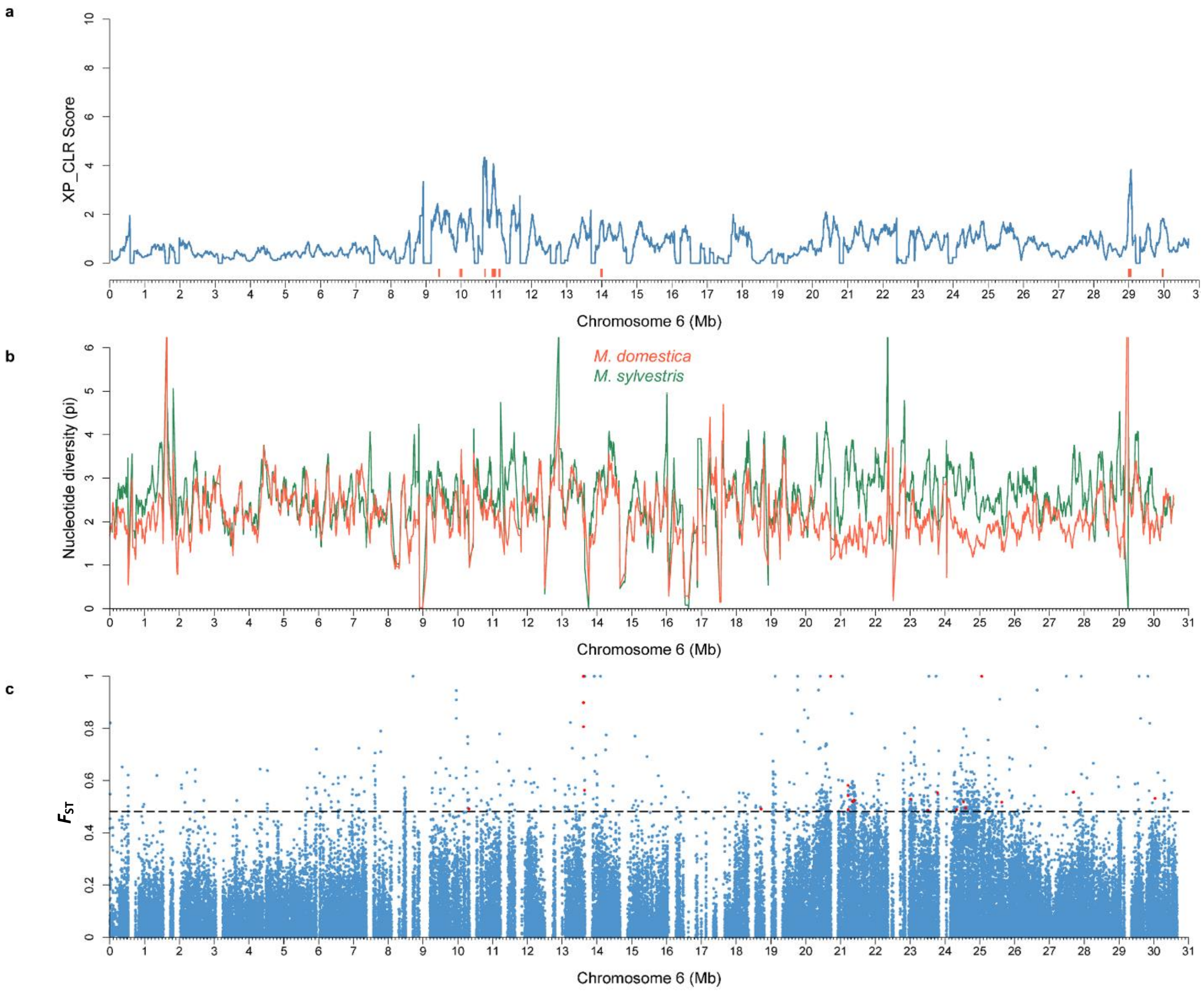

Supplementary Figure 10 (continued)

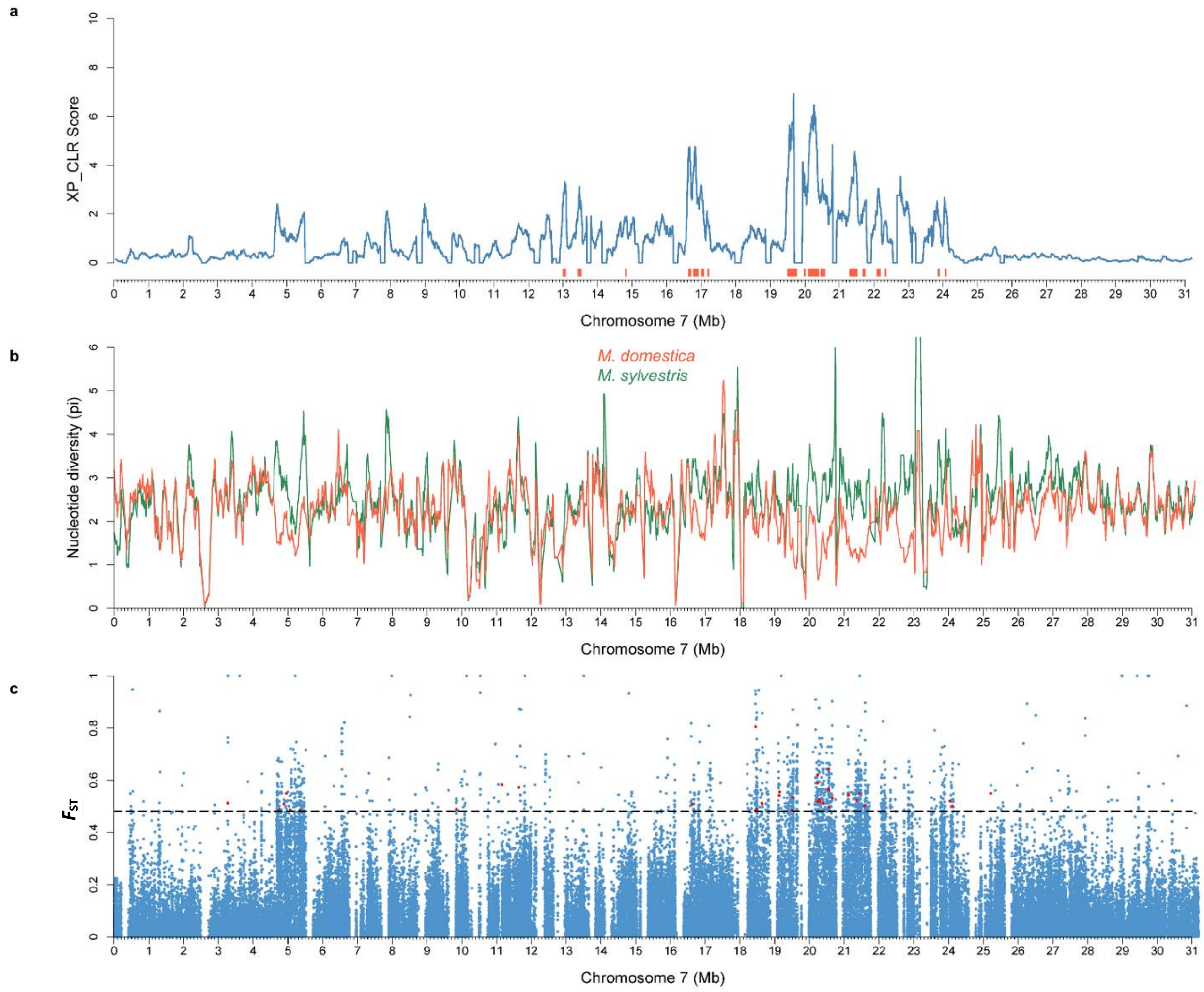

Supplementary Figure 10 (continued)

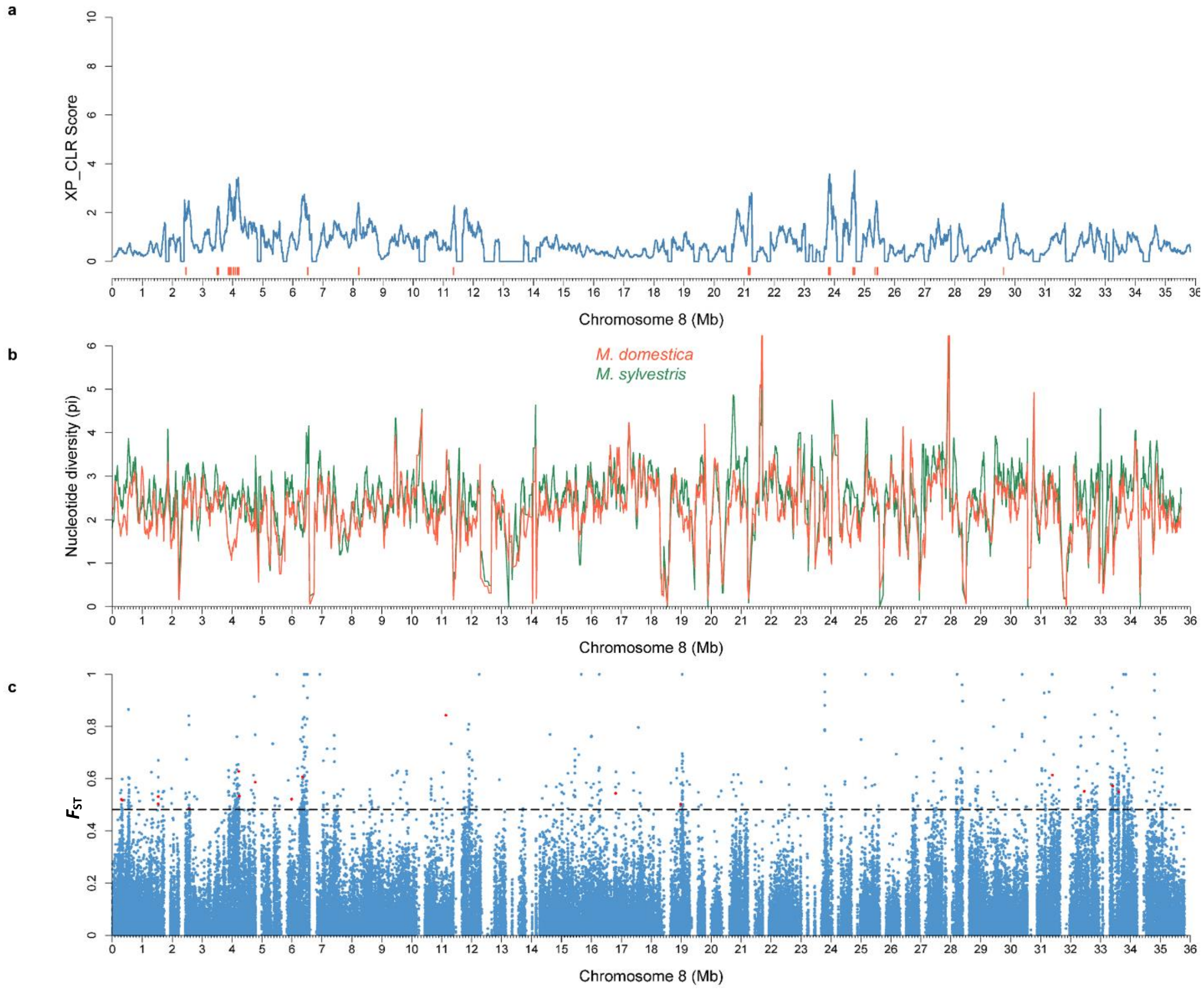

Supplementary Figure 10 (continued)

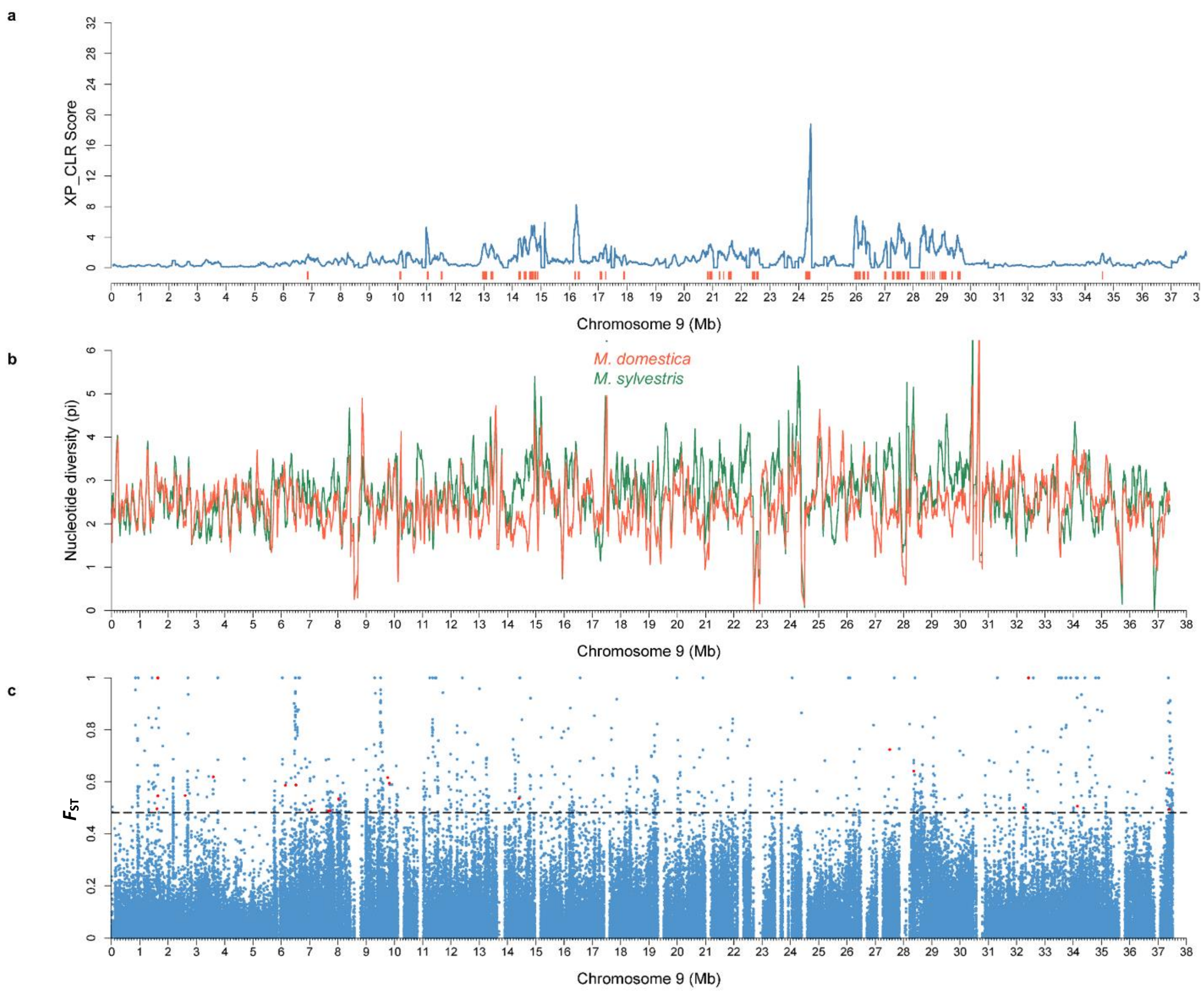

Supplementary Figure 10 (continued)

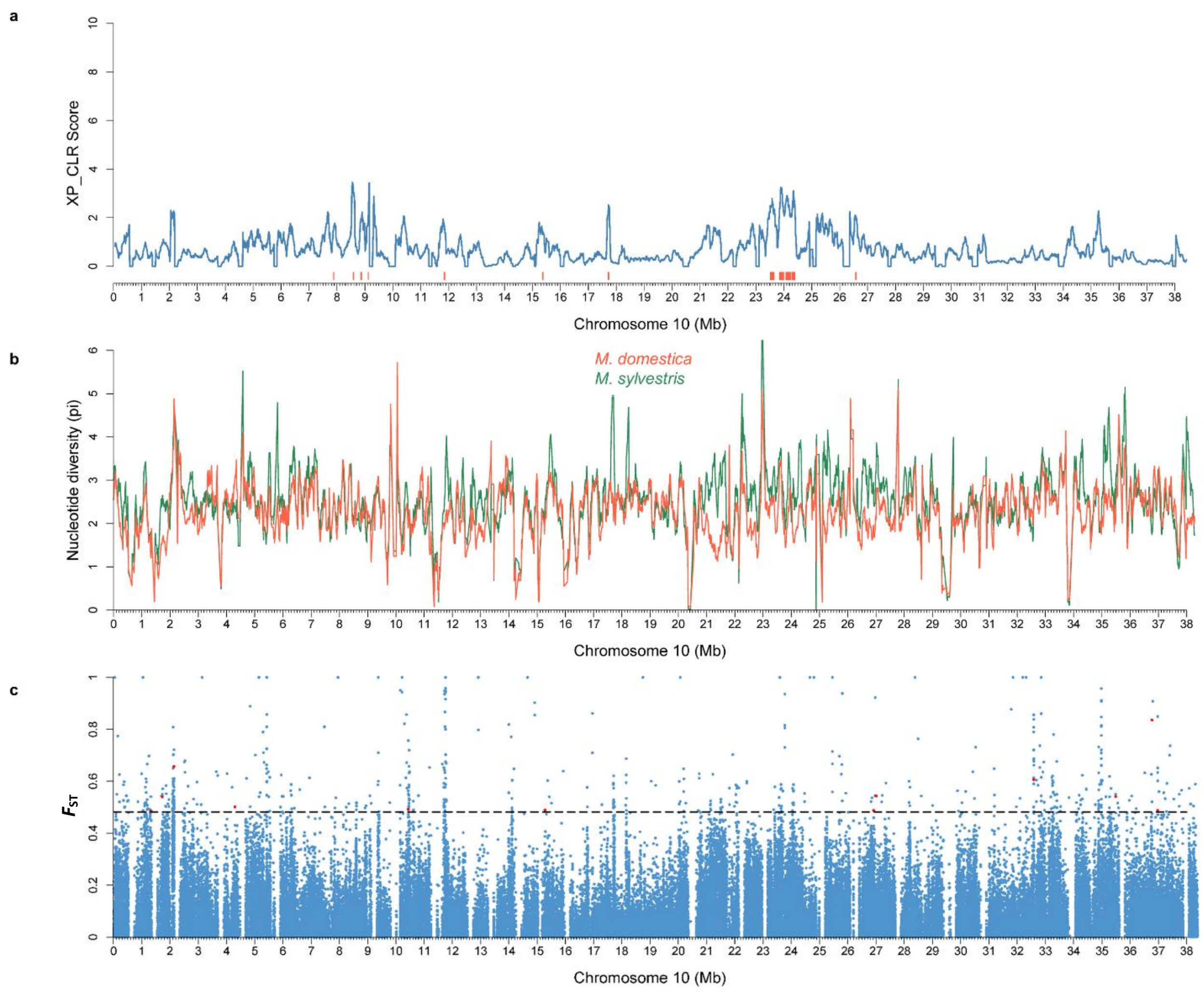

Supplementary Figure 10 (continued)

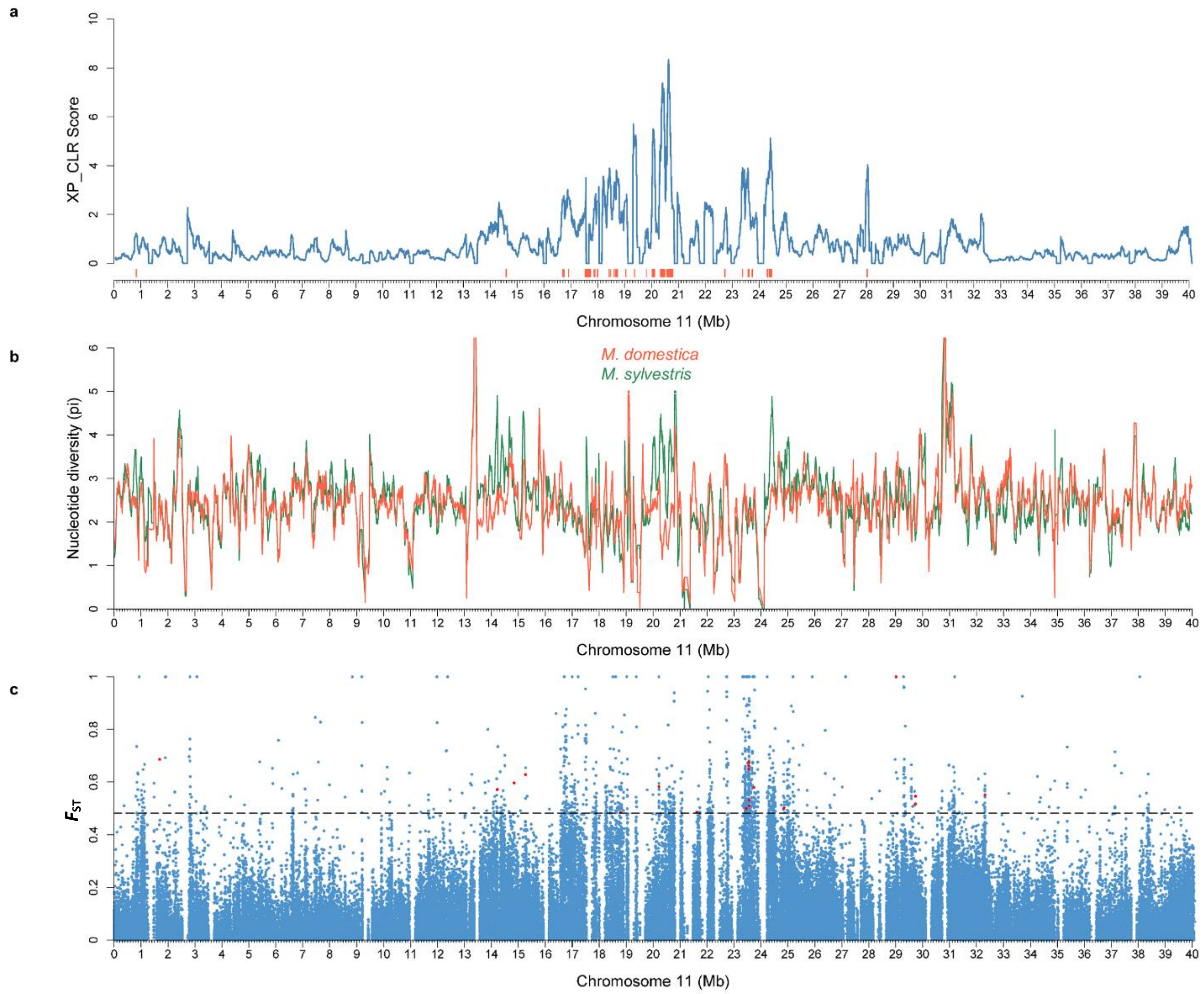

Supplementary Figure 10 (continued)

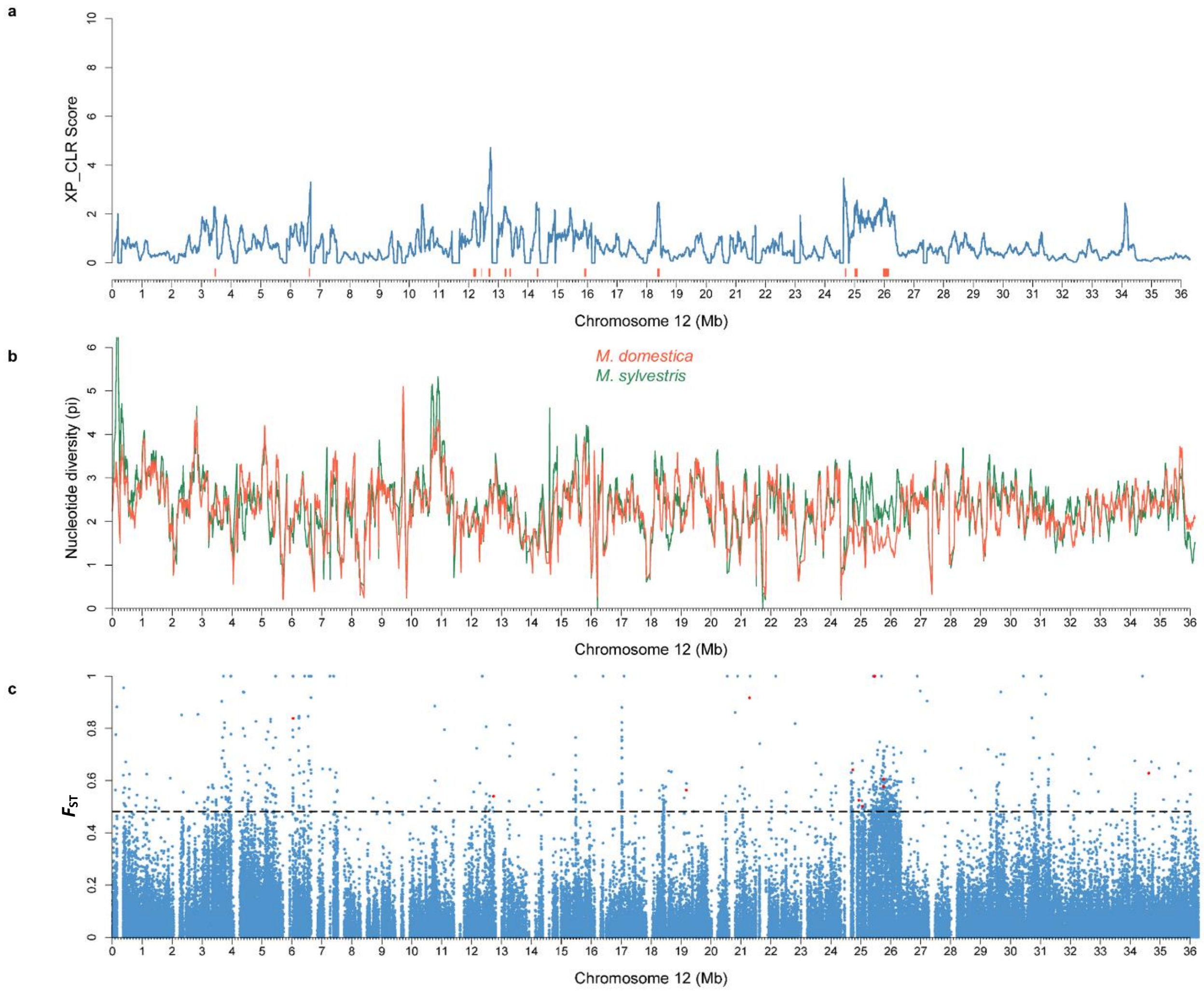

Supplementary Figure 10 (continued)

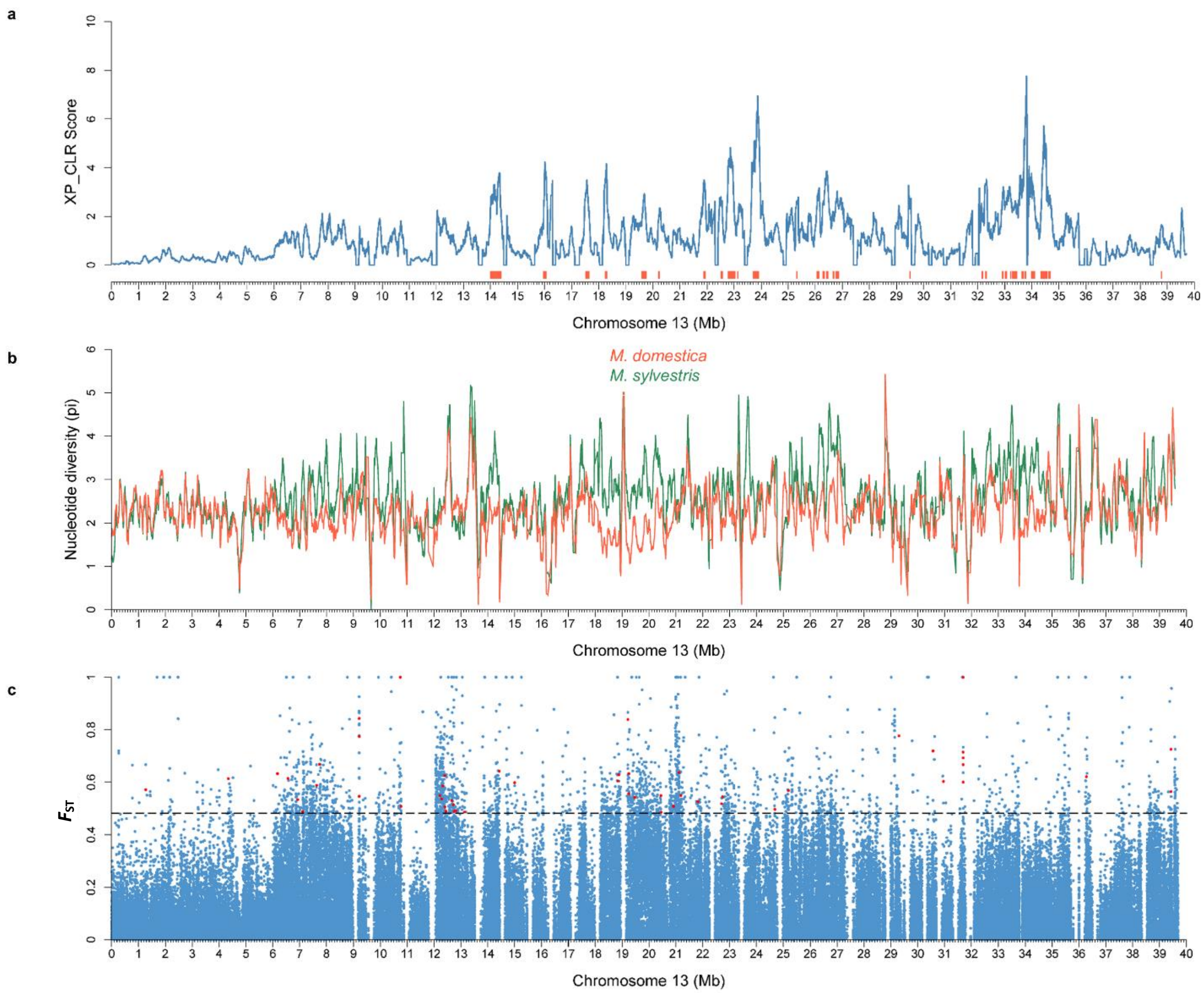

Supplementary Figure 10 (continued)

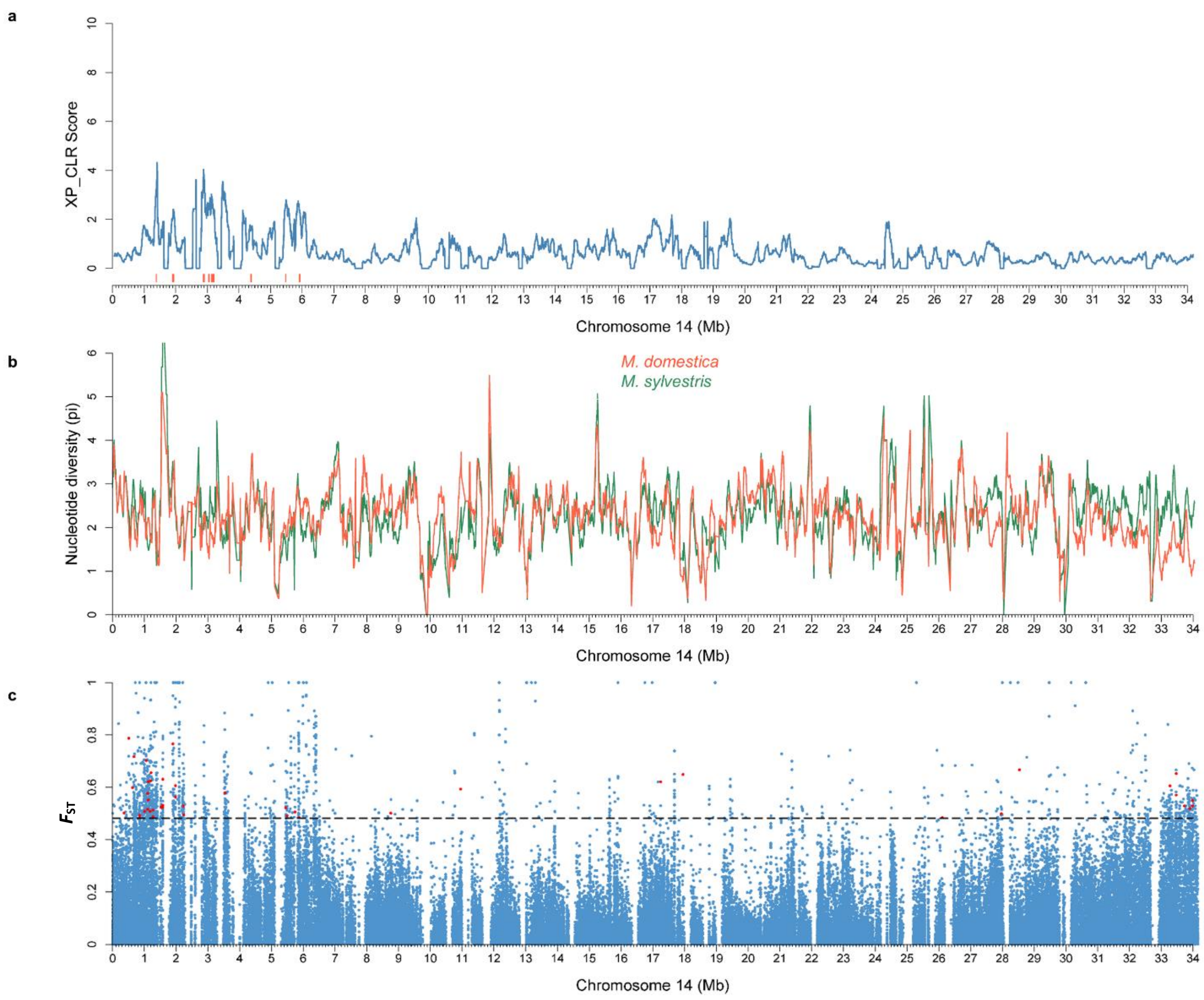

Supplementary Figure 10 (continued)

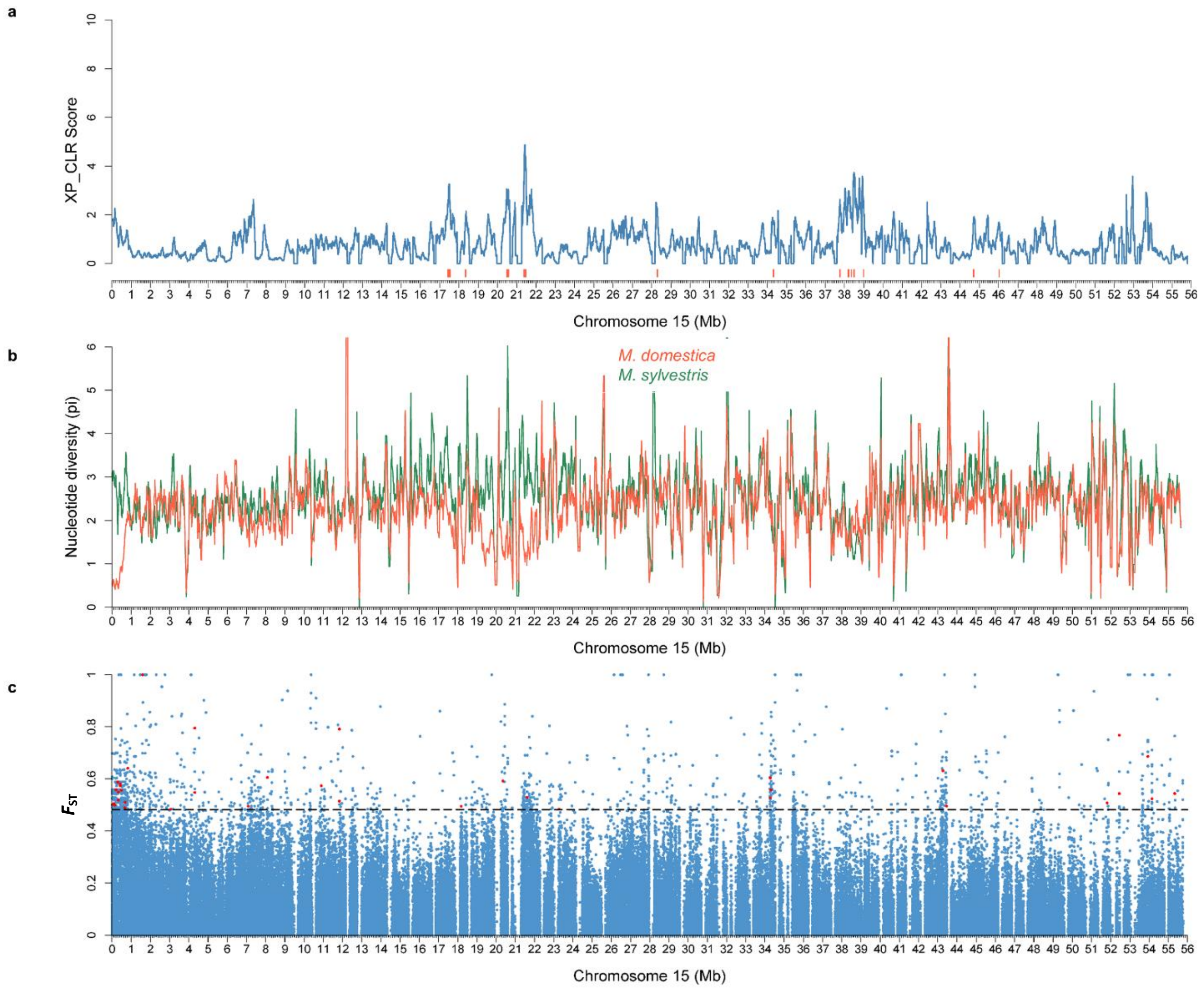

Supplementary Figure 10 (continued)

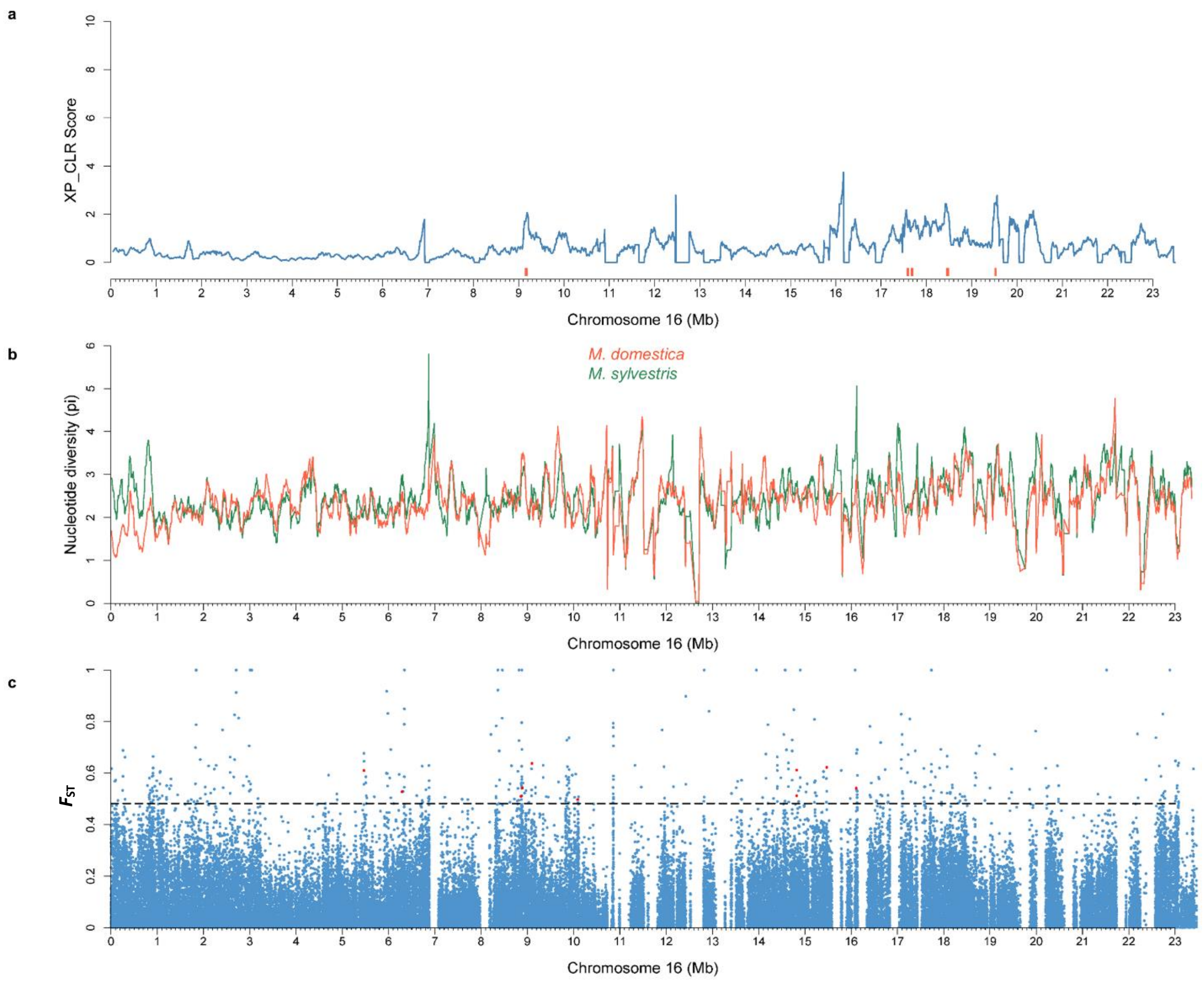

Supplementary Figure 10 (continued)

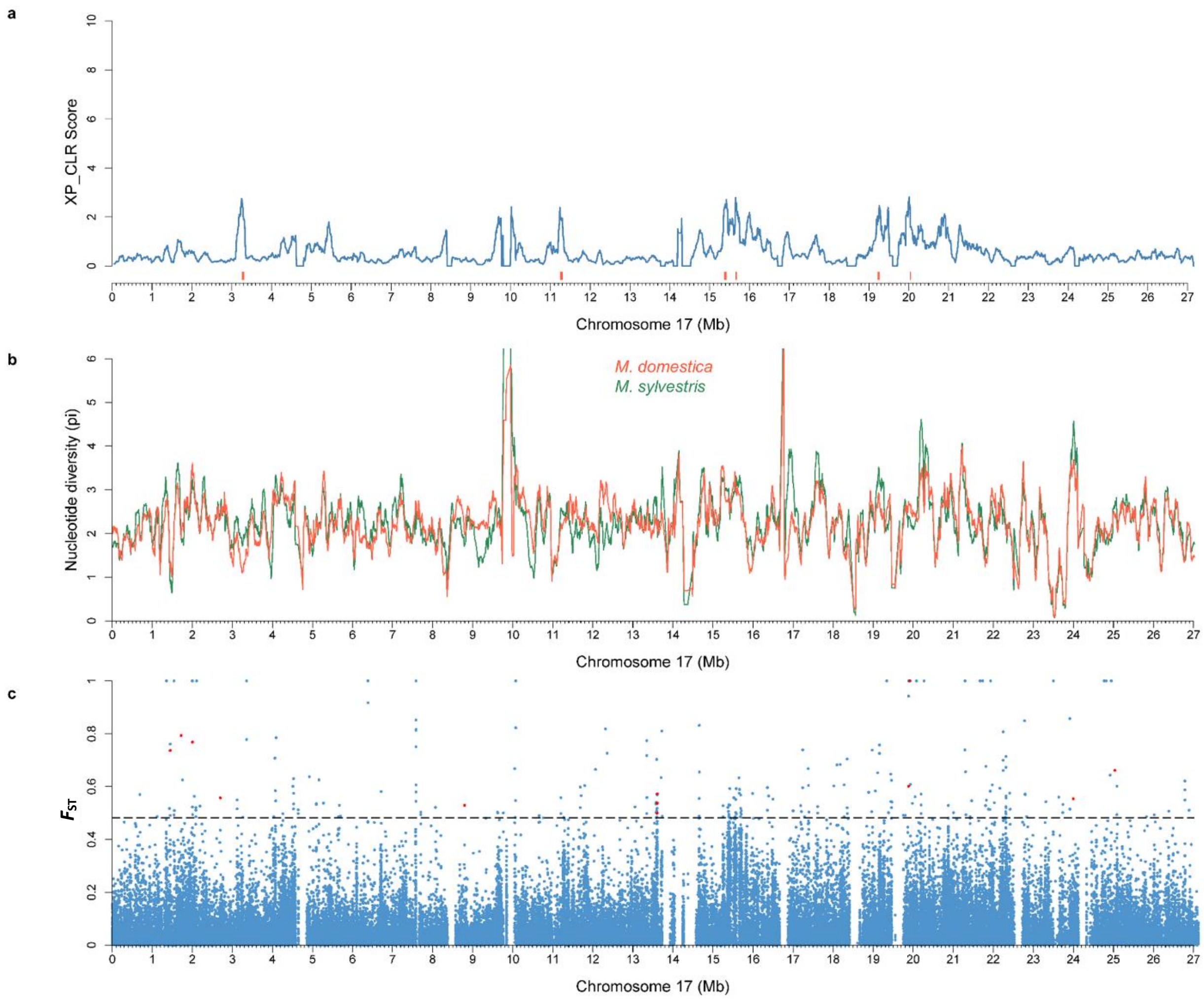

Supplementary Figure 10 (continued)

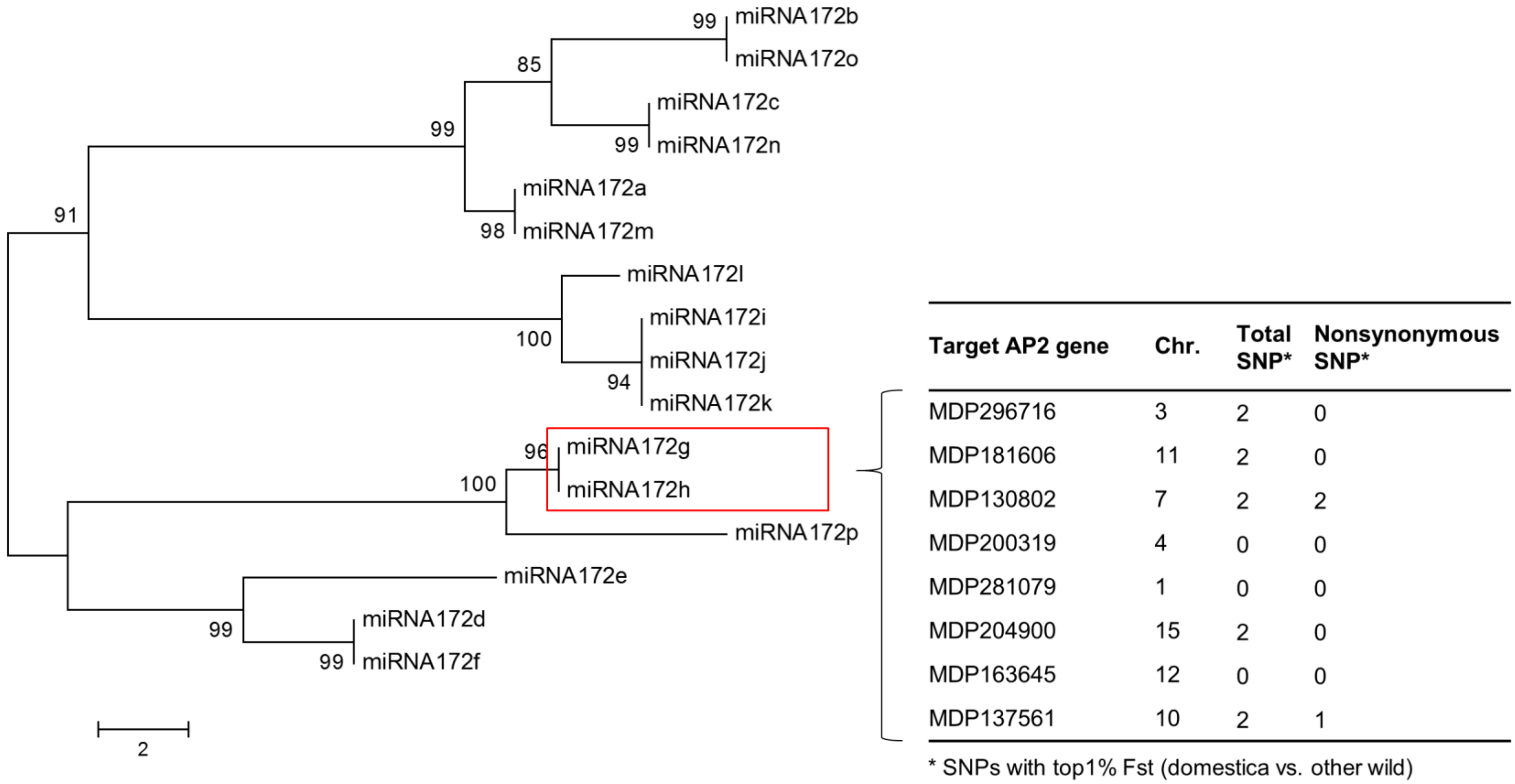

Target AP2 gene	Chr.	Total SNP*	Nonsynonymous SNP*
MDP296716	3	2	0
MDP181606	11	2	0
MDP130802	7	2	2
MDP200319	4	0	0
MDP281079	1	0	0
MDP204900	15	2	0
MDP163645	12	0	0
MDP137561	10	2	1

* SNPs with top1% Fst (domestica vs. other wild)

Supplementary Figure 11 Phylogenetic and target analyses of apple miRNA172 genes. Neighbor-joining phylogenetic tree based on precursor sequences of the 16 apple miRNA172 genes. *MiRNA172g* and *miRNA172h*, sharing the same precursor sequences, are the only two miRNA172 genes containing significantly differentiated SNPs between *M. domestica* and other wild species. The eight target genes of *miRNA172g* or *miRNA172h* are listed together with the numbers of highly divergent SNPs for each gene.

Supplementary Table 1. Summary of sequencing and variations identified in each species group

Groups	Group size	Raw bases (Gb)	Cleaned bases (Gb)	Cleaned mean depth per sample	No. SNPs	No. small indels
M. domestica	35	579	336	12.9	3,376,976	138,182
M. sieversii (Kazakhstan)	15	239	108	9.7	2,190,136	100,593
M.sieversii (Xinjiang)	14	211	159	15.3	1,165,236	76,945
M. sylvestris	10	158	73	9.8	2,694,391	103,486
other wild species	43	590	384	12.0	6,493,769	346,498
Total	117	1,776	1,060	12.2	7,218,060	431,597

Supplementary Table 2. Statistics of SNPs and genes classified by their physical locations in the apple genome.

Genomic locations	No. SNPs
**Total**	7,218,060
**Intergenic & UTRs**	**5,376,659**
Two kb upstream of ORF*	783,578
Two kb downstream of ORF	821,096
Other intergenic regions	3,771,985
**Intron**	**1,086,191**
Intron/exon border (2 bp)	4,543
Other intron regions	1,081,648
**CDS**	**755,210**
Nonsynonymous	308,841
Synonymous	303,136
Nonsense	8,670
Heterozygous base in genome	134,563

*ORF: open reading frame.

Supplementary Table 3. Phenotypic data of apple fruit skin color used for GWAS

Sample ID	Species	Accession ID	Fruit skin color*
Dom_01	*Malus domestica*	280400	5
Dom_02	*Malus domestica*	107196	0
Dom_03	*Malus domestica*	589596	3
Dom_04	*Malus domestica*	588806	3
Dom_05	*Malus domestica*	588853	2
Dom_06	*Malus domestica*	589841	4
Dom_07	*Malus domestica*	588785	4
Dom_08	*Malus domestica*	588844	4
Dom_09	*Malus domestica*	392303	4
Dom_10	*Malus domestica*	588880	0
Dom_11	*Malus domestica*	590185	4
Dom_12	*Malus domestica*	589053	3
Dom_13	*Malus domestica*	588943	4
Dom_14	*Malus domestica*	588872	4
Dom_15	*Malus domestica*	589478	4
Dom_16	*Malus domestica*	589520	0
Dom_17	*Malus domestica*	588850	5
Dom_18	*Malus domestica*	590186	4
Dom_19	*Malus domestica*	588859	1
Dom_20	*Malus domestica*	589687	3
Dom_21	*Malus domestica*	644174	3
Dom_22	*Malus domestica*	589673	0
Dom_23	*Malus domestica*	NA	1
Dom_33	*Malus domestica*	588873	0
Dom_28	*Malus domestica*	337319	2
Dom_31	*Malus domestica*	588816	1
Dom_24	*Malus domestica*	590184	1
Sie_K_01	*Malus sieversii*	632626	2

Sie_K_02	*Malus sieversii*	613951	0
Sie_K_03	*Malus sieversii*	613987	3
Sie_K_04	*Malus sieversii*	613988	2
Sie_K_05	*Malus sieversii*	613971	3
Sie_K_06	*Malus sieversii*	613976	4
Sie_K_07	*Malus sieversii*	613978	3
Sie_K_08	*Malus sieversii*	613979	3
Sie_K_09	*Malus sieversii*	657028	0
Sie_K_10	*Malus sieversii*	657067	0
Sie_K_11	*Malus sieversii*	657002	2
Sie_K_12	*Malus sieversii*	657017	0
Sie_K_13	*Malus sieversii*	GMAL4011	2
Sie_K_14	*Malus sieversii*	657094	0
Sie_K_15	*Malus sieversii*	657101	1
Syl_01	*Malus sylvestris*	588908	3
Syl_02	*Malus sylvestris*	633825	1
Syl_03	*Malus sylvestris*	633827	0
Syl_04	*Malus sylvestris*	633824	0
Syl_05	*Malus sylvestris*	633826	0
Syl_06	*Malus sylvestris*	369855	1
Syl_07	*Malus sylvestris*	589382	0
Syl_08	*Malus sylvestris*	619168	2
Syl_09	*Malus sylvestris*	101888	2
Syl_10	*Malus sylvestris*	392302	5
Ang_01	*Malus angustifolia*	589727	0
Bac_01	*Malus baccata*	322713	4
Cor_01	*Malus coronaria*	588927	4
Flo_01	*Malus florentina*	589385	1
Fus_01	*Malus fusca*	589933	4
Ioe_01	*Malus ioensis*	590009	0
Kir_01	*Malus kirghisorum*	590043	0
Ori_01	*Malus orientalis*	644252	1
Pru_02	*Malus prunifolia*	594102	5
Pum_01	*Malus pumila*	589225	5
Hyb_01	*Malus hybrid*	LNYS2	3
Asi_01	*Malus asiatica var. rinki*	LNYS3	5

Hyb_02	*Malus hybrid*	LNYS4	3
Rob_01	*Malus robusta*	LNYS5	4
Spe_01	*Malus spectabilis*	LNYS6	0
Mic_01	*Malus micromalus*	LNYS7	4
Pru_01	*Malus prunifolia*	LNYS9	5
Roc_01	*Malus rockii*	LNYS10	5
Mel_01	*Malus melliana*	LNYS11	3
Pla_01	*Malus platycarpa*	LNYS12	1
Cer_01	*Malus ceracifolia*	LNYS13	5
Hup_01	*Malus hupehensis*	LNYS14	5
Xia_01	*Malus xiaojinensis*	LNYS15	4

*Fruit skin colors were scored as: 0, green; 1, yellow; 2, light red (green base with pink-red area and/or stripes less than half of fruit); 3, moderate red (green-yellow base with blush more than half of fruit); 4, red (without green or yellow base); 5, dark red.

Supplementary Note 1. Apple cultivation in ancient China

Apple is an ancient fruit crop that has been cultivated in China for more than 2000 years. Apples in China, including domesticated cultivars that are collectively called 'Chinese soft apples', are mostly from local open-pollinated hybridizations and native wild species with desirable fruit or floral traits[1-3]. The native wild species cultivated as dessert apples in China mainly include *M. asiatica* and *M. prunifolia*, and those as ornamental species consist of *M. robusta*, *M. spectabilis* and *M. micromalus*. Based on pollen morphological, physiological and molecular marker analyses[4-7], as well as our population structure analyses, Chinese soft apples probably originated from *M. sieversii* in Kazakhstan, the west side of the Tian Shan Mountain, which were spread to central and north China via the Silk Road eastward and developed into many local cultivars through hybridizations with native wild species.

Supplementary Note 2. Apple SNPs

The final set contains 7,218,060 SNPs, which allowed to develop a total of 1,039,264 tag SNPs, representing a useful resource for apple breeding. Among the final set of 7,218,060 SNPs, 3,376,976 (46.8 %) are from the group of *M. domestica*, 2,190,136 (30.3%) from *M. sieversii* in Kazakhstan, 1,165,236 (16.1%) from *M. sieversii* in Xinjiang, 2,694,391 (37.3%) from *M. sylvestris* and 6,493,769 (90.0%) from other wild species (**Supplementary Table 1**). Accessions of *M. sieversii* in Xinjiang have the lowest number of SNPs which may be caused by its isolated geographic location compared to other widely spread species. The high number of SNPs and the emergence of unique SNPs in *M. domestica* may be caused by hybridizations with different local wild species on its way of spreading all over the world, as well as diverse breeding directions in modern apple improvement. The other wild species group, which contains 43 accessions,

harbored almost twice the number of SNPs in the *M. domestica* group that comprises 35 accessions. This demonstrates the highly diverse gene pool in wild apples, a valuable genetic resource for apple improvement.

In the final set of 7,218,060 SNPs, 755,210 (10.5%) were located in gene coding regions, 1,086,191 (15.0%) in intragenic regions, and 1,604,674 (22.2%) within 2 kb upstream or downstream of genes (**Supplementary Table 2**). Among SNPs in coding regions, 308,841 were nonsynonymous and 303,136 synonymous. The ratio of nonsynonymous to synonymous SNPs was 1.02, which is higher than that of Arabidopsis[8] (0.83) but lower than that of peach[9] (1.31), soybean[10] (1.37), and rice[11] (1.29), indicating apple might have a lower rate of genome variations leading to phenotypic modifications than other domesticated grain and fruit crops.

Supplementary Note 3. Pedigree evaluation for hybrid wild species

Except for seven *Malus* species with homogeneous genetic background, all other 17 wild species in this study appear to have evolved from an admixture of ancestral populations, demonstrating the heterogeneous nature of the genetic makeup of apple. Phylogenetic and bi-ancestral clustering analyses showed that *M. asiatica* and *M. prunifolia*, two native Chinese species bearing small-to-medium-sized fruits, were the closest wild relatives to *M. sieversii*, consistent with previous findings using a limited number of molecular markers and conserved sequences[4,6,7] (**Supplementary Fig. 4**). Meanwhile, Chinese soft apple cultivars (e.g., Hyb_01 ‘Pinpo’ and Hyb_02 ‘Xiango’) were clustered with wild apple species rather than with *M. domestica*, suggesting that these cultivars might be ancient hybrids between large-fruited *M. sieversii* and small-fruited local wild species. Structure analysis suggests that the two Chinese native species, *M. asiatica* and *M. prunifolia* and one hybrid soft apple cultivar (Hyb_01 ‘Pinpo’) may have originated from crosses between *M. sieversii* in

Kazakhstan and *M. baccata* (**Supplementary Fig. 5a**). The pedigree record of *M. asiatica* in the USDA-GRIN database is consistent with our findings (**Supplementary Data 2**). Our data are also in agreement with that *M. robusta* is likely from hybridizations between *M. baccata* and *M. prunifolia*, but oppose to the pedigree of *M. platycarpa* suggested in the GRIN database as derived from the cross between *M. domestica* and *M. coronaria* (**Supplementary Fig. 5b** and **Supplementary Data 2**). In addition, several rootstocks (G.41 and G.210) clearly inherited wild ancestry from their progenitor, *M. robusta* (**Supplementary Fig. 5c**), thus it was not unexpected that they clustered with wild accessions in the phylogenetic tree (**Fig. 1a**). Furthermore, our phylogenetic tree supported the reclassification of *M. kirghisorum* (Kir_01) to *M. sieversii* var. *kirghisorum*, as it tightly clustered with other *M. sieversii* accessions (**Fig. 1a** and **Supplementary Data 2**).

Supplementary Note 4. A global map of apple evolutionary history

Here we infer a global map of apple evolutionary history based on our population structure and phenotypic analyses of the 117 *Malus* accessions. Ancient wild species of genus *Malus* found in the southwestern and central parts of inland China were spread presumably by birds to what is now called the ranges of Tian Shan Mountains, where they evolved into a new species, *M. sieversii*, with medium-to-large sized fruits[12]. In Kazakhstan, the west side of the Tian Shan Mountains, *M. sieversii* were domesticated to be ancient cultivated apples and their fruits, seeds and even scion woods were carried westwards along the Silk Road from central Asia to Europe, and then underwent extensive introgressions from local wild species, such as European crabapple *M. sylvestris*, to become modern *M. domestica*. Later, *M. domestica* apples were introduced to America and Australia by European colonists. During apple domestication, its fruit quality

has been remarkably improved. Soft, mild sweet, medium-large sized *M. sieversii* apples have evolved into slightly firmer, sweeter and larger ancient domesticated apples. After hybridization with firm, sour, bitter-sweet, small-sized *M. sylvestris* apples, *M. domestica* apples have inherited advantageous traits from both parents and became ‘a perfect fruit’ with firm and crispy texture, large fruit size, and savory flavor and aroma.

In contrast to the firm, crispy modern *M. domestica* apples which were introduced to China in the 1870s[2], ancient Chinese soft apples are very likely to have originated from soft *M. sieversii* apples in Kazakhstan and spread into central and north China via the Silk Road or earlier trade routes[2,3]. During this eastward distribution, *M. sieversii* apples were hybridized with distinct native wild species including *M. baccata*, yielding different hybrid species, such as *M. asiatica*, *M. prunifolia* and *M. robusta*. Due to the significant advantage of fruit texture in modern domesticated apples over Chinese soft apples, modern *M. domestica* apples are now widely grown for fruit production in many parts of the world, including China.

Supplementary Note 5. Linkage disequilibrium patterns

Linkage disequilibrium (LD) levels may vary among populations due to several reasons, such as population size, mating system (self-pollinating and out-crossing), selective pressure and recombination rates[13,14]. We calculated r^2 between each pair of SNPs and the decay of r^2 with increasing physical distance between SNPs. The average distance over which LD decays to half of its maximum value in apple populations are all within 1 kb (161 bp in *M. domestica*, 256 bp in *M. sieversii* in Kazakhstan, 994 bp in *M. sieversii* in Xinjiang,

375 bp in *M. sylvestris* and 224 bp in other wild species) (**Fig. 2b**). In general, LD decays very rapidly in apple, similar to maize[15] but much faster than other crop species such as peach[9], tomato[16], rice[11], soybean[10] and cucumber[17].

Supplementary Note 6. Genome-wide association study of fruit skin color in apple

Apple domestication is a weak selection process characterized by the undetectable reduction in genome-wide genetic diversity and the fast LD decay in cultivated apples; therefore, a set of high-density markers should be critical in conducting trait association studies in apple. Here, using genome-wide apple SNPs as a demonstration, we performed GWAS of fruit skin color in 75 apple accessions. We detected a total of 54 SNPs that were significantly associated with fruit skin color, of which four were nonsynonymous (**Supplementary Data 3**). Forty-eight out of the 54 SNPs were located on chromosome 9 covering a continuous region from 32.13 Mb to 34.75 Mb (**Supplementary Fig. 6**). Within this region, there were 161 genes including three MYB transcription factors (*MDP0000259614*, *MYB1/A/10*; *MDP0000879675*, *MYB5*; and *MDP0000159011*, *MYB6*) and one flavonoid 3' hydroxylase (*MDP0000629122*) (**Supplementary Data 4**). *MYB1/A/10* has been reported to control apple fruit skin anthocyanin levels[18-20]. Our GWAS for fruit skin color successfully pinpointed a very narrow peak interval harboring *MYB1/A/10*, the key gene regulating the studied trait, even with a small set of phenotypic data, showing the power of high-density SNPs in enhancing the resolution in GWAS, especially for perennial crops like apple with fast LD decays.

Supplementary References

1. Li, Y. A primarily modern systematics of genus *Malus* Mill. in the world. *J. Fruit Sci.* **13**, 82-92 (1996).
2. Li, Y. An investigation and studies on the origin and evolution of *Malus domestica* Borkh. in the world. *Acta Hortic. Sinica* **26**, 213-220 (1999).
3. Luo, G. The cultivation history of apple in China. *J Beijing For. Univ.* **13**, 15-25 (2014).
4. Zhu, Y., Cao, M., Xu, Z., Wang, K. & Zhang, W. Polygenetic relationship between Xinjiang wild apple (*Malus sieversii* Roem.) and Chinese apple (*Malus* x *domestica* subsp. *chinesnsis*) based on ITS and *mat*K sequences. *Acta Hortic. Sinica* **41**, 227-239 (2014).
5. Li, X. *et al.* Ornamental crabapple pollen morphology and its taxonomic applications. *Acta Hortic. Sinica* **35**, 1175-1182 (2008).
6. Li, J. *et al.* The genetic relationship of 41 *Malus* germplasm resources revealed by AFLP analysis. *J. Fruit Sci.* **30**, 725-731 (2013).
7. Gao, Y., Liu, F., Yufen, C. & Wang, K. Analysis of genetic relationship for *Malus* germplasm resources by SSR markers. *J. Fruit Sci.* **24**, 129-134 (2007).
8. Clark, R.M. *et al.* Common sequence polymorphisms shaping genetic diversity in *Arabidopsis thaliana*. *Science* **317**, 338-342 (2007).
9. Cao, K. *et al.* Comparative population genomics reveals the domestication history of the peach, *Prunus persica*, and human influences on perennial fruit crops. *Genome Biol.* **15**, 415 (2014).
10. Lam, H.M. *et al.* Resequencing of 31 wild and cultivated soybean genomes identifies patterns of genetic diversity and selection. *Nat. Genet.* **42**, 1053-1059 (2010).
11. Xu, X. *et al.* Resequencing 50 accessions of cultivated and wild rice yields markers for identifying agronomically important genes. *Nat. Biotechnol.* **30**, 105-111 (2012).
12. Harris, S.A., Robinson, J.P. & Juniper, B.E. Genetic clues to the origin of the apple. *Trends Genet.* **18**, 426-430 (2002).
13. Gupta, P.K., Rustgi, S. & Kulwal, P.L. Linkage disequilibrium and association studies in higher plants: present status and future prospects. *Plant Mol. Biol.* **57**, 461-485 (2005).
14. Flint-Garcia, S.A., Thornsberry, J.M. & IV, B. Structure of linkage disequilibrium in plants. *Ann. Rev. Plant Biol.* **54**, 357-374 (2003).
15. Gore, M. A. et al. A first-generation haplotype map of maize. *Science* **326**, 1115–1117 (2009).
16. Lin, T. *et al.* Genomic analyses provide insights into the history of tomato breeding. *Nat. Genet.* **46**, 1220-1226 (2014).
17. Qi, J. *et al.* A genomic variation map provides insights into the genetic basis of cucumber domestication and diversity. *Nat. Genet.* **45**, 1510-1515 (2013).
18. Ban, Y. *et al.* Isolation and functional analysis of a *MYB* transcription factor gene that is a key regulator for the development of red coloration in apple skin. *Plant Cell Physiol.* **48**, 958-970 (2007).
19. Takos, A.M. *et al.* Light-induced expression of a *MYB* gene regulates anthocyanin biosynthesis in red apples. *Plant Physiol.* **142**, 1216-1232 (2006).
20. Espley, R.V. *et al.* Red colouration in apple fruit is due to the activity of the MYB transcription factor, *MdMYB10*. *Plant J.* **49**, 414-427 (2007).
